# Supplementary material for: Linking ATP and allosteric sites to achieve superadditive binding with bivalent EGFR kinase inhibitors
Source: Commun Chem. 2024 Feb 20;7:38. doi: 10.1038/s42004-024-01108-3 (PMC10879502; doi:10.1038/s42004-024-01108-3)
Supplement: Supplementary file 2 — Supplemental Information [file 42004_2024_1108_MOESM2_ESM.pdf]

**Supplementary Information**

# Linking ATP and allosteric sites to achieve superadditive binding with bivalent EGFR kinase inhibitors

Florian Wittlinger[1,#], Blessing C. Ogboo[2,#], Ekaterina Shevchenko[1,3,4], Tahereh Damghani[2], Calvin D. Pham[2], Ilse K. Schaeffner[5,6], Brandon T. Oligny[2], Surbhi P. Chitnis[2], Tyler S. Beyett[5,6,12], Alexander Rasch[1], Brian Buckley[7], Daniel A. Urul[8], Tatiana Shaurova[9], Earl W. May[8], Erik M. Schaefer[8], Michael J. Eck[5,6], Pamela A. Hershberger[9], Antti Poso[1,3,4,10], Stefan A. Laufer[1,3,4]*, David E. Heppner[2,9,11]*

1. Department of Pharmaceutical and Medicinal Chemistry, Institute of Pharmaceutical Sciences, Eberhard Karls Universität Tübingen, Auf der Morgenstelle 8, 72076 Tübingen, Germany

2. Department of Chemistry, University at Buffalo, The State University of New York, Buffalo, NY, 14260, USA

3. Cluster of Excellence iFIT (EXC 2180) "Image-Guided and Functionally Instructed Tumor Therapies" Eberhard Karls Universität Tübingen, 72076 Tübingen, Germany.

4. Tübingen Center for Academic Drug Discovery & Development (TüCAD2), 72076 Tübingen, Germany

5. Department of Cancer Biology, Dana-Farber Cancer Institute, Boston, MA, 02215 USA

6. Department of Biological Chemistry and Molecular Pharmacology, Harvard Medical School, Boston, MA, 02115 USA

7. Department of Cell Stress Biology, Roswell Park Comprehensive Cancer Center, Buffalo, NY,14203, USA

8. AssayQuant Technologies, Inc., Marlboro, MA, 01752, USA

9. Department of Pharmacology and Therapeutics, Roswell Park Comprehensive Cancer Center, Buffalo, NY,14203, USA

10. School of Pharmacy, University of Eastern Finland, 70210 Kuopio, Finland

11. Department of Structural Biology, University at Buffalo, The State University of New York, Buffalo, NY, 14260, USA

12. Present Address: Department of Pharmacology and Chemical Biology, Emory University School of Medicine, 5119 Rollins Research Center, 1510 Clifton Rd, Atlanta, GA 30322, USA

*These authors jointly supervised this work:

David E. Heppner (0000-0002-0722-5160) davidhep@buffalo.edu

Stefan A. Laufer (0000-0001-6952-1486) stefan.laufer@uni-tuebingen.de

[#]These authors contributed equally:

Florian Wittlinger, Blessing C. Ogboo

**Table of Contents**

1. Chemical structures of matched *C*-linked fragments and HTRF dose-response curves ……….S3

2. Linking Coefficients and electron denisity maps of *C*- and *N*-linked bivalent inhibitors ………S4

3. X-ray crystallographic images ………………………………..…………………………..S5

4. Geometry comparisons in compounds **1** and **2** from co-crystal structures ...…………………..…S6

5. Interaction patterns of compounds **1** and **2** ………………………………………………..…S7

6. RMSD of compound **1** and **2** and docking pose and superpositions of compound **4** …………...S8

7. Docking pose of **4** in complex with EGFR……………………………………………......S9

8. B-factors of compounds **1** and **2** ………………………………..……………………....S10

9. Prime MM-GBSA Δ*G* Energy components……………..………………………....….S11

10. Prime MM-GBSA Ligand Energy……………..……………...……………….….……S12

11. Hydrophobic interactions frequency ……………..……………...…………….……......S13

12. Thermodynamic Profile of Water in the binding pocket……………...….……………….....S14

13. Torsional profile of compound **1** and **2** ……………..……………...….…………….......S15

14. Full and uncropped Western blot images…………………….…………………………....S16

15. Cellular results ……………………………….………………………………………..S20

16. Metabolic stability and kinome selectivity screening of **4** ……………………………...……...S22

17. Supplementary Note 1 ……………..………………………………….……….……..…S40

18.Supplemenatary References ………………………..……………….………………..…S62

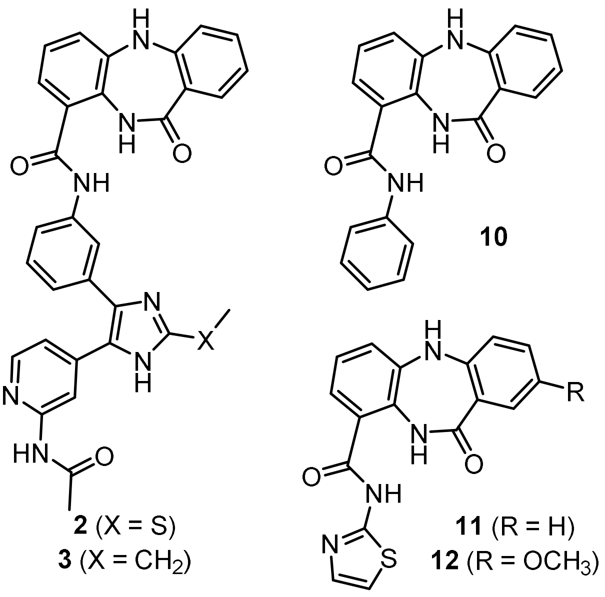


**Scheme S1:** Chemical structures of matched *C*-linked fragments **10-12** and potent *C*-linked bivalent ATP-allosteric inhibitors **2-3**. The thiazol-2-yl substituent of **11-12** was derived from allosteric inhibitor EAI045.[1]

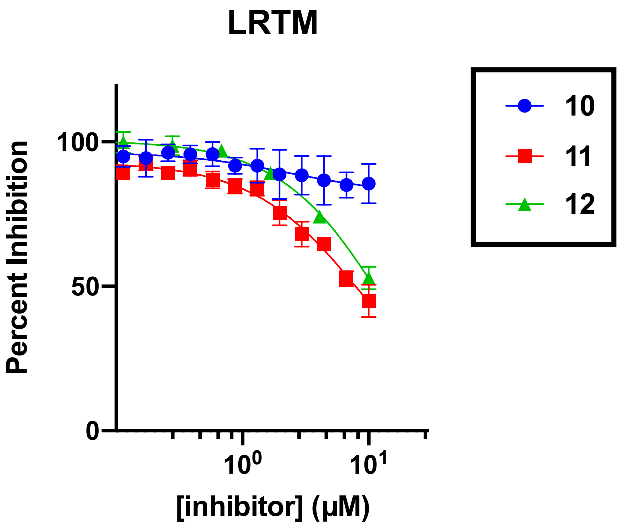


**Figure S1.** HTRF dose-dependent impact on LRTM activity of C-linked matched allosteric motifs **10-12**. Error bars represent standard deviation of three experimental replicates.

**Table S1. Calculation of Linking Coefficients for *C*- and *N*-linked bivalent inhibitors.** Linking Coefficients (*E*) of bivalent N-linked methylene (**1**) and *C*-linked amides (**2,3**) based on enzymatic $IC_{50}$ values of LRTM. $IC_{50}$ values for each molecule are in the white shaded cells and the corresponding calculated upper- or lower-limit *E* values are in the gray shaded central cell. All activity assay measurments performed with [ATP] = 100 μM. $IC_{50}$ values considered for LRTM EGFR biochemical activity assays (Table 1). Calculation performed as done previously, $E = IC_{50}(\text{bivalent}) / [IC_{50}(\text{ATP})^* IC_{50}(\text{Allosteric})])^2$.

| Bivalent | ATP | | Allosteric |
|---|---|---|---|
| | **5** (5.8 μM) | **6** (> 10 μM) | |
| **1** (> 10 μM) | > 4.4 × $10^7$ $M^{-1}$ | > 2.6 × $10^7$ $M^{-1}$ | **8** (39 nM) |
| **1** (> 10 μM) | > 3.4 × $10^7$ $M^{-1}$ | > 2.0 × $10^7$ $M^{-1}$ | **9** (52 nM) |
| **2** (59 pM) | < 1.0 $M^{-1}$ | < 0.59 $M^{-1}$ | **10** (> 10 μM) |
| **3** (51 pM) | < 0.88 $M^{-1}$ | < 0.51 $M^{-1}$ | **10** (> 10 μM) |

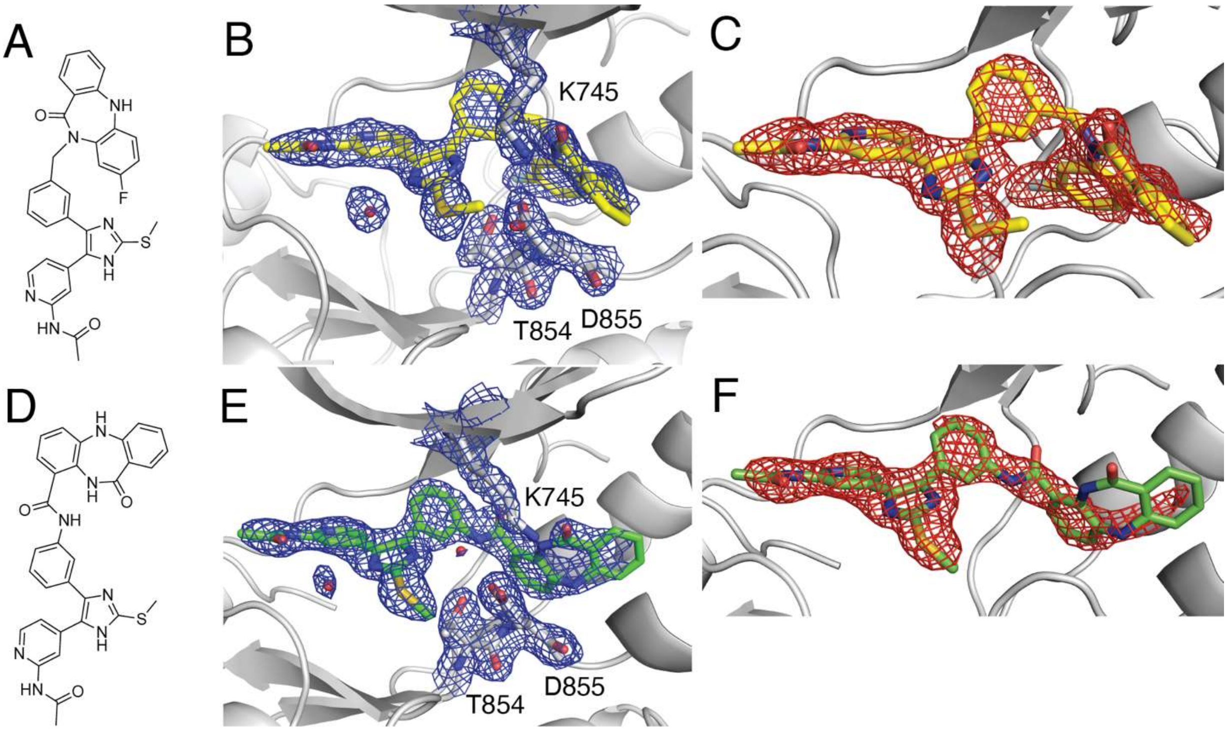


**Figure S2**: **Electron density maps for co-crystal structures of 1 and 2 in complex with EGFR(T790M/V948R).** Crystal structure electron densities of **1** (PDB ID 8FV3) and **2** (PDB ID 8FV4) in complex with EGFR(T790M/V948R). a) Chemical structure of **1**. b) $2F_o$-$F_c$ electron density map of **1** (blue, concoured to 1.5σ) and surrounding side chains and solvent waters. c) $F_o$-$F_c$ simulated annealing omit map of **1** (red, contoured to 3σ). d) Chemical structure of **2**. e) $2F_o$-$F_c$ electron density map of **2** (blue, concoured to 1.5σ) and surrounding side chains and solvent waters. f) $F_o$-$F_c$ simulated annealing omit map of **2** (red, contoured to 3σ).

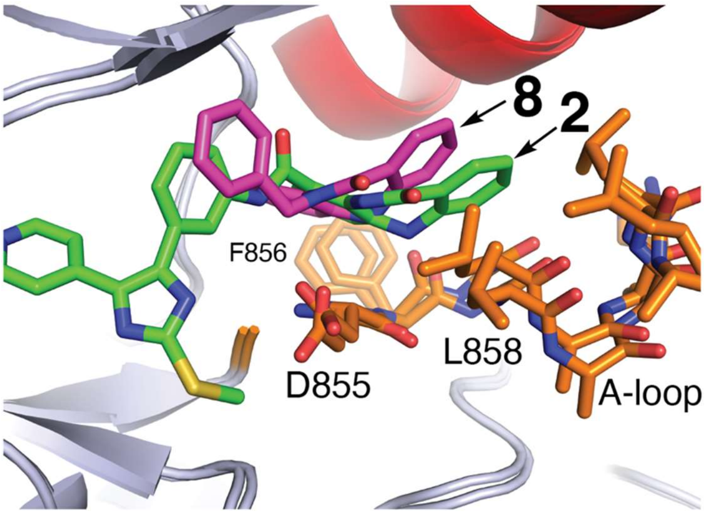


**Figure S3**: **Overlay of the bivalent inhibitor 2 with the allosteric inhibitor 8 (DDC4002).** An overlay of **2** (green, PDB ID 8FV4) with DDC4002 (magenta, PDB ID 6P1D) shows that the activation loop (A-loop, orange in both structures) conformation in both structures is practically identical.

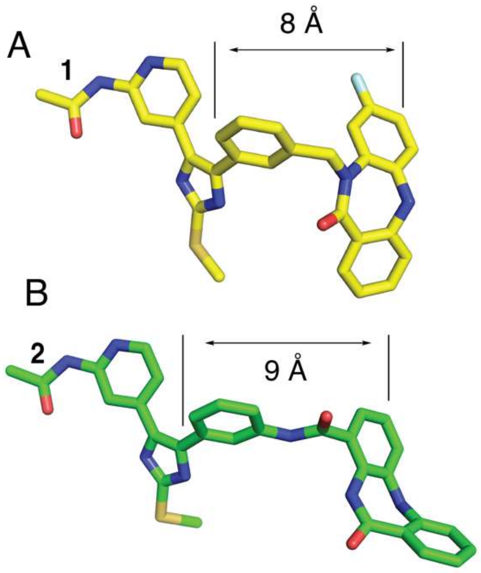


**Figure S4**: Selected comparisons of the geometries in **1** and **2** from co-crystal structures (See Figure 5 main text). a) and b) show similar distances from the imidazole at the ATP site and the benzo phenyl ring anchored in the hydrophobic pocket.

| EGFR residue | Kinase subunit | LN4503 (cmp. 1) (141) | LN5993 (cmp. 2) (140) |
|---|---|---|---|
| L718 | β-I | 8% WB BB | 9% WB BB |
| K745 | catalytic Lysine | 7% Pi-cation<br>25% HB SC<br>37% WB SC<br>17% WB SC | 33% Pi-cation<br>22% HB SC<br>44% WB SC<br>22% WB SC |
| M793 | Hinge | 98% HB BB<br>80% HB BB | 97% HB BB<br>65% HB BB |
| C797 | Linker | 10% WB BB | 24% WB BB<br>11% WB BB |
| D800 | αD-helix | 5% WB SC | |
| T854 | xDFG | - | 39% HB SC |
| D855 | DFG | 29% WB SC<br>45% WB SC<br>29% WB SC | 75% WB SC<br>10% HB BB |
| F856 | DFG | 62% Pi-Pi stacking<br>84% HB BB | 90% Pi-Pi stacking<br>83% HB BB |
| K860 | a.l | 6% Pi-cation | - |
| L862 | a.l | 6% WB SC | - |

**Table S2**: **Interaction patterns of compound 1 and compound 2** derived from molecular dynamics simulations. The percentages shown indicate the fraction of simulation time during which the ligand is in contact with specific protein residue. Molecular dynamics timescale for each replica is 1µs. For each compound, a total of 10 replicas were run, resulting in a timescale of 10µs for each compound. The minimum contact strength displayed in the table is 5%. xDFG – the preceding amino acid residue of the DFG-motif, a.l. – activation loop, β-I – beta sheet I. HB – hydrogen bond, WB – water bridge, BB – backbone, SC – side chain. (i.e., HB SC indicates a hydrogen bond between the ligand and the side chain of a protein residue).

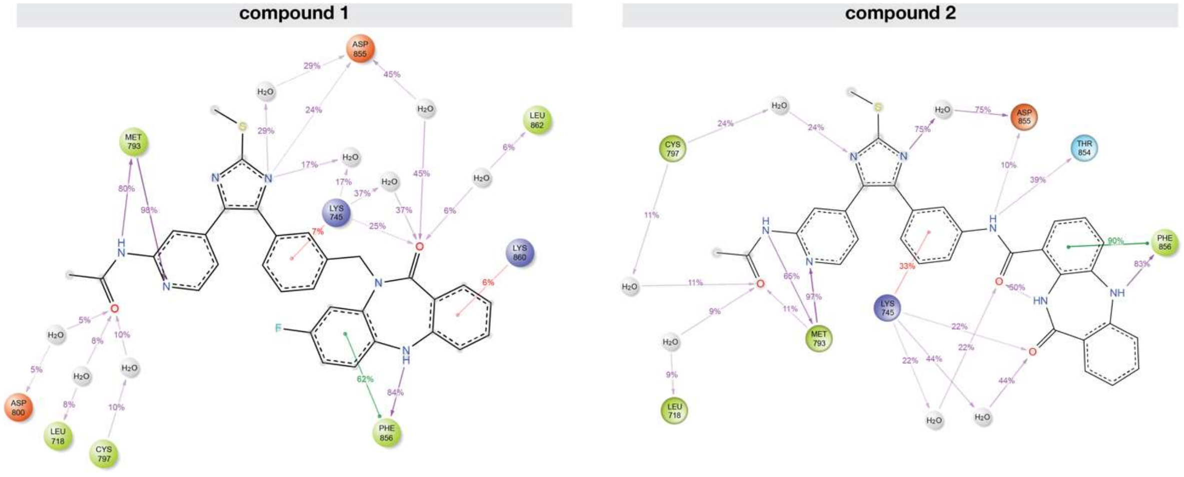


**Figure S5**: **Interaction patterns of compound 1 and compound 2 derived from molecular dynamics simulations**. Polar residues are represented in blue, hydrophobic residues in green, and charged residues in orange. Green lines represent π–π stacking interactions, red lines represent π-cation interactions, and purple lines represent hydrogen bonds. a dashed line is used to indicate side-chain interactions, while a straight line represents backbone interactions. The strength of each interaction is indicated by the percentage value displayed along the line, reflecting its occurrence during the simulation time. Only interactions that persist for more than 5% of the simulation time are shown in the figure.

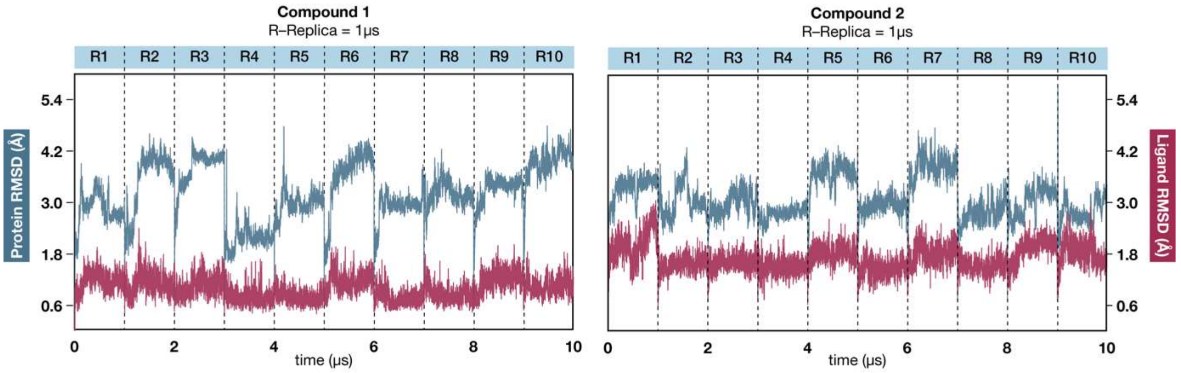


**Figure S6: RMSD of compound 1 and compound 2** derived from molecular dynamics simulations. Protein RMSD was calculated for Ca–atoms, displayed in blue colour. Ligand RMSD was calculated as aligned on the protein structure, measuring the fluctuations of the ligand with respect to the protein. The ligand RMSD is depicted in red colour. The RMSD values are divided by replicas: R1–replica 1, R2 – replica 2, etc. Each replica corresponds to a 1000ns (1μs) simulation time.

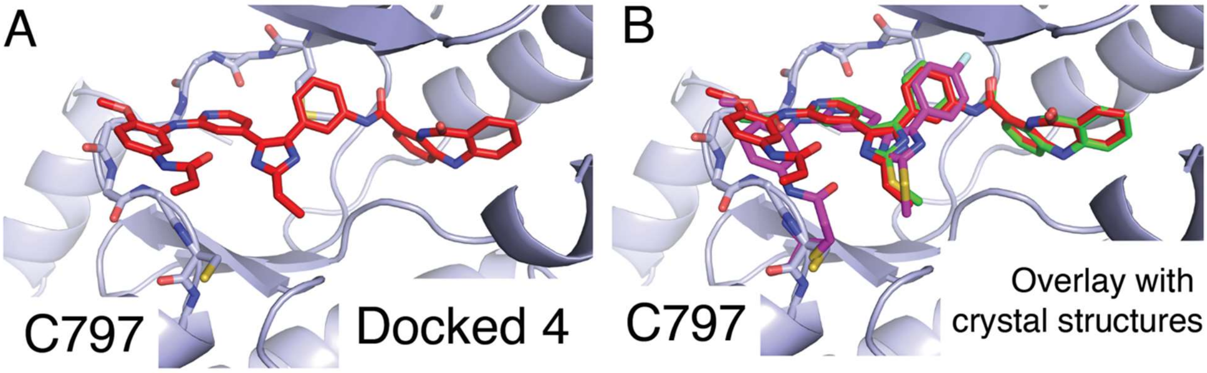


**Figure S7**: a) Compound **4** (red) was docked into the EGFR(T790M/V948R) kinase domain created from the co-crystal structure with **2** (PDB ID 8FV4) using Schrodinger Glide software (Glide Score = -18.29, Docking = -16.96). The docking pose shows the expected binding pose and positioning of the acrylamide warhead in proximity to the C797 residue. b) Superposition of the docking pose of compound **4** (red) and EGFR(T790M/V948R) co-crystal structures of the bivalent **2** (green, PDB ID 8FV4) and ATP-site covalent inhibitor **7** (magenta, PDB ID 6V6K) indicating that **4** fully spans the orthosteric and allosteric pocket.

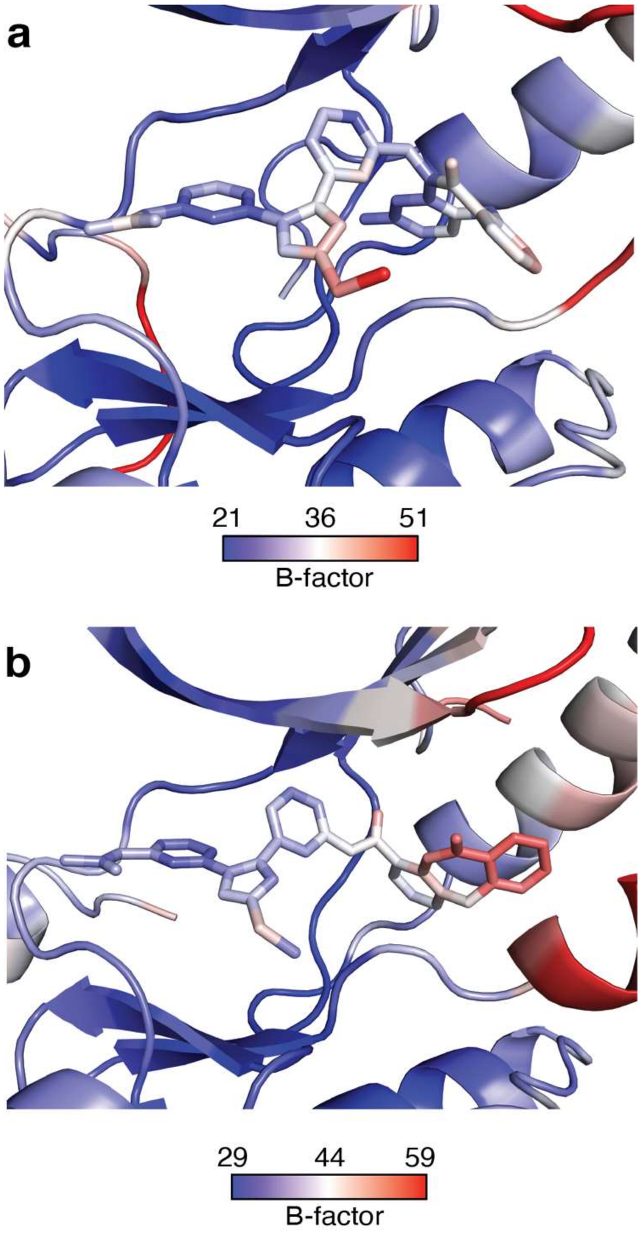


**Figure S8.** Frontfacing images of the binding modes of a) **1** and b) **2** with blue-to-red gradient showing variable B-factors (units of Å$^2$) with protein structure ribbon indicating the relative B-factors of the protein backbone. The overall average B-factors are 36 Å$^2$ for **1** (PDB ID 8FV3) and 44 Å$^2$ for **2** (PDB ID 8FV4).

**Table S3**: **Prime MM-GBSA ΔG Energy components derived from Molecular Dynamics simulations.** Δ*G* Binding Free Energy and its components: hydrogen-bonding, π–π packing, and Van der Waals. Average and standard deviation is calculated for each simulation replica with 1µs simulation time (Replica 1…Replica 10), as well as for the merged MD trajectories (10µs/compound). SD – standard deviation

| | **ΔG kcal/mol** | | **Hydrogen-bonding kcal/mol** | | **π–π packing kcal/mol** | | **Van der Waals kcal/mol** | | **Ligand Energy kcal/mol** | |
|---|---|---|---|---|---|---|---|---|---|---|
| Average SD | cmp 1 | cmp 2 | cmp 1 | cmp 2 | cmp 1 | cmp 2 | cmp 1 | cmp 2 | cmp 1 | cmp 2 |
| Replica 1 | -121.6 6.9 | -128.9 4.7 | -2.2 0.5 | -2.1 0.2 | -1.4 0.5 | -0.7 0.1 | -93.6 3.8 | -96.7 3.2 | -14.5 0.09 | -51.2 |
| Replica 2 | -114.1 4.8 | -124.7 4.7 | -1.8 0.3 | -2.0 0.2 | -0.6 0.2 | -0.7 0.1 | -91.1 3.2 | -93.5 2.9 | -14.6 0.01 | -51.2 1.0 |
| Replica 3 | -117.8 5.0 | -126.2 5.0 | -2.0 0.5 | -2.1 0.2 | -0.6 0.1 | -0.7 0.1 | -90.3 3.1 | -95.1 3.1 | -14.6 0.01 | -51.2 1.0 |
| Replica 4 | -116.8 4.9 | -125.1 4.6 | -1.7 0.3 | -2.1 0.2 | -0.6 0.1 | -0.7 0.1 | -90.7 2.9 | -93.8 2.9 | -14.6 0.01 | -51.3 0.9 |
| Replica 5 | -116.1 4.8 | -124.9 4.7 | -1.9 0.4 | -2.1 0.2 | -0.7 0.2 | -0.7 0.1 | -91.8 3.2 | -94.2 2.8 | -14.7 0.01 | -51.3 1.0 |
| Replica 6 | -113.1 5.1 | -127.1 6.0 | -1.7 0.3 | -2.4 0.3 | -0.7 0.2 | -0.9 0.2 | -89.3 2.9 | -95.2 3.3 | -14.7 0.01 | -51.3 0.9 |
| Replica 7 | -116.4 5.3 | -122.8 4.8 | -1.9 0.3 | -2.2 0.2 | -0.7 0.1 | -0.8 0.2 | -91.3 3.6 | -93.7 3.7 | -14.7 0.01 | -51.3 0.9 |
| Replica 8 | -110.6 5.8 | -128.7 4.7 | -1.6 0.4 | -2.1 0.3 | -0.6 0.2 | -0.8 0.1 | -89.0 3.6 | -96.0 2.7 | -14.7 0.01 | -51.34 0.9 |
| Replica 9 | -118.5 5.0 | -125.1 5.2 | -1.7 0.2 | -1.9 0.2 | -0.7 0.1 | -0.7 0.2 | -91.5 2.6 | -92.8 3.0 | -14.8 0.01 | -51.4 0.9 |
| Replica 10 | -118.3 5.9 | -125.4 5.0 | -1.9 0.4 | -2.2 0.3 | -0.6 0.1 | -0.8 0.1 | -92.5 3.1 | -92.3 3.3 | -14.9 0.05 | -51.4 0.9 |
| **Merged trajectory values (10µs/compound)** | | | | | | | | | | |
| Average SD | -116.4 6.2 | -125.9 5.3 | -1.8 0.4 | -2.1 0.2 | -0.7 0.3 | -0.8 0.2 | -91.1 3.5 | -94.3 3.4 | -14.7 0.1 | -51.3 0.9 |

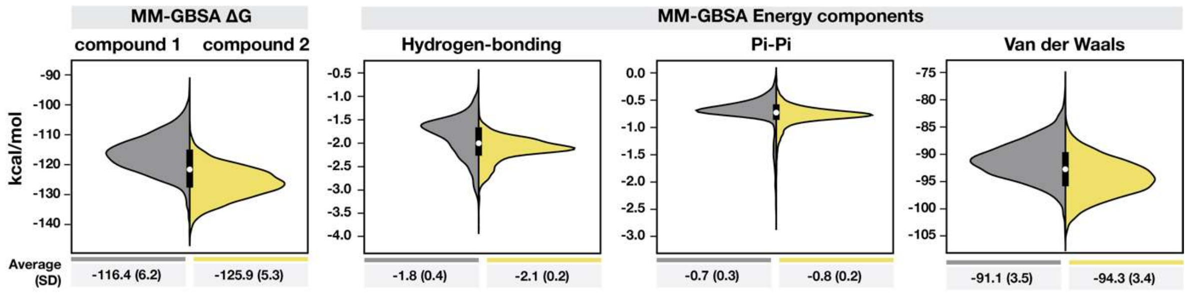


**Figure S9**: **Prime MM-GBSA ΔG Energy components derived from Molecular Dynamics simulations.** Violin plot representation of Δ*G* Binding Free Energy. The white circle represents the median. The thick black bar in the center represents the interquartile range (IQR), and the thin grey line represents the rest of the data within 1.5 times of the IQR. On each side of the grey line is a kernel density estimation to show the distribution shape of data. Wider sections represent a higher probability that members of the population will take on the given value. SD – standard deviation. Average and standard deviation is shown for the merged MD trajectories (10μs/compound).

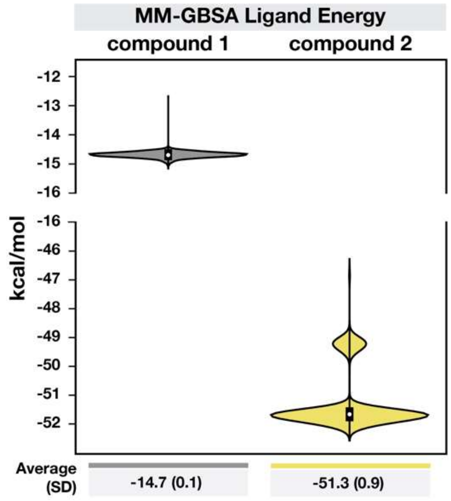


**Figure S10**. **Prime MM-GBSA Ligand Energy derived from Molecular Dynamics simulations.**. Violin plot representation of MM-GBSA Ligand Energy derived from Molecular Dynamics simulations. The white circle represents the median. The thick black bar in the center represents the interquartile range (IQR), and the thin grey line represents the rest of the data within 1.5 times of the IQR. On each side of the grey line is a kernel density estimation to show the distribution shape of data. Wider sections represent a higher probability that members of the population will take on the given value. Average and standard deviation is shown for the merged MD trajectories (10μs/compound). SD – standard deviation.

**Table S4**: **Hydrophobic interaction frequency** derived from molecular dynamics simulations**.** The total amount indicates the amount of ligand–EGFR hydrophobic contacts detected in all MD frames for a specific system (Amount of frames for each system 10000, timescale 10μs). I–VIII = β-sheets I–VIII; g.l. = G-rich loop, b.l. = loop connecting αC-helix to IV; GK = gatekeeper; linker = loop connecting the hinge to αD-helix; c.l. = catalytic loop; xDFG = DFG-motif plus one preceding amino acid residue; a.l. = activation loop.

| **Res. in contact** | **EGFR binding site region** | **Compound 1** | **Compound 2** |
|---|---|---|---|
| **Total amount of hydrophobic interactions** | | **36397** | **39317** |
| **L718** | β-I | 2769 | 2610 |
| **A722** | g.l | 181 | - |
| **F723** | g.l | 659 | 1966 |
| **V726** | β-II | 1002 | 2000 |
| **A743** | β-III | 8327 | 8492 |
| **L747** | β-III | 49 | 61 |
| **I759** | αC | - | 188 |
| **A763** | αC | - | 292 |
| **A764** | αC | 4 | - |
| **V765** | αC | 2 | - |
| **M766** | αC | 1888 | 3000 |
| **A767** | αC | - | 8 |
| **C775** | b.l | 13 | 53 |
| **L777** | β-IV | 1609 | 3701 |
| **L788** | β-V | 2655 | 897 |
| **M790** | GK | 8578 | 5473 |
| **L792** | Hinge | 46 | 84 |
| **M793** | Hinge | 4 | 27 |
| **C797** | linker | 118 | 85 |
| **L844** | β-VII | 5588 | 4574 |
| **F856** | DFG | 171 | 147 |
| **L858** | a.l | - | 3712 |
| **L861** | a.l | 1194 | 1900 |
| **L862** | a.l | 1542 | 42 |

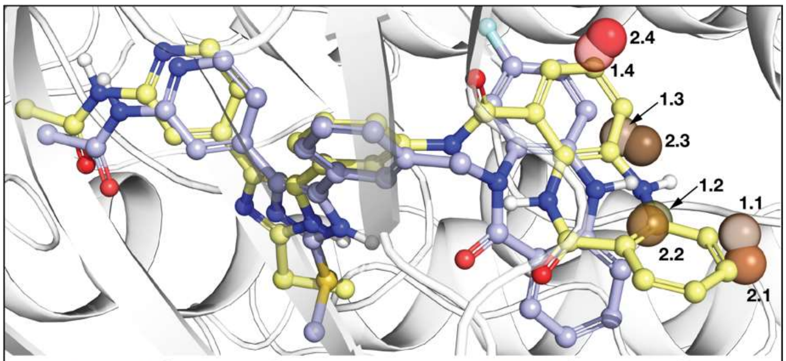


| Compound 1 | | | | Compound 2 | | | |
|---|---|---|---|---|---|---|---|
| **№** | **ΔG** | **ΔH** | **-TΔS** | **№** | **ΔG** | **ΔH** | **-TΔS** |
| **1.1** | 4.8 | 3.9 | 0.9 | **2.1** | 4.1 | 2.6 | 1.6 |
| **1.2** | 3.9 | 2.4 | 1.5 | **2.2** | 2.5 | 1.1 | 1.4 |
| **1.3** | 4.7 | 2.6 | 2.1 | **2.3** | 4.0 | 1.6 | 2.4 |
| **1.4** | 7.2 | 5.4 | 1.9 | **2.4** | 5.9 | 3.1 | 2.8 |

**Figure S11: Thermodynamic Profile of Water in the binding pocket of compound 1 and compound 2.** The thermodynamic properties presented in this table were derived from WaterMap simulations of the binding pocket of compound 1 and compound 2 in the unbound state of EGFR. Thermodynamic properties are interpreted as following: ***ΔG*** justifies the average interaction free energy between a water molecule in a binding site and the rest of the system compared to bulk water. A higher Δ*G* value indicates that the water molecule is less stable and content in the binding site. ***ΔH*** represents the average interaction enthalpy between a water molecule at the site and the rest of the system in relation to bulk water. Positive Δ*H* values indicate that the water is less stable in the protein binding site compared to bulk water, while negative values indicate that the water is more stable in the protein binding site compared to bulk water. **-TΔS** Excess entropy of the water molecules associated with the water site compared to that of bulk water molecules. Larger values correspond to water molecules that have more significant entropic penalties. High-energy water molecules can contribute to conformational entropy in the binding site.

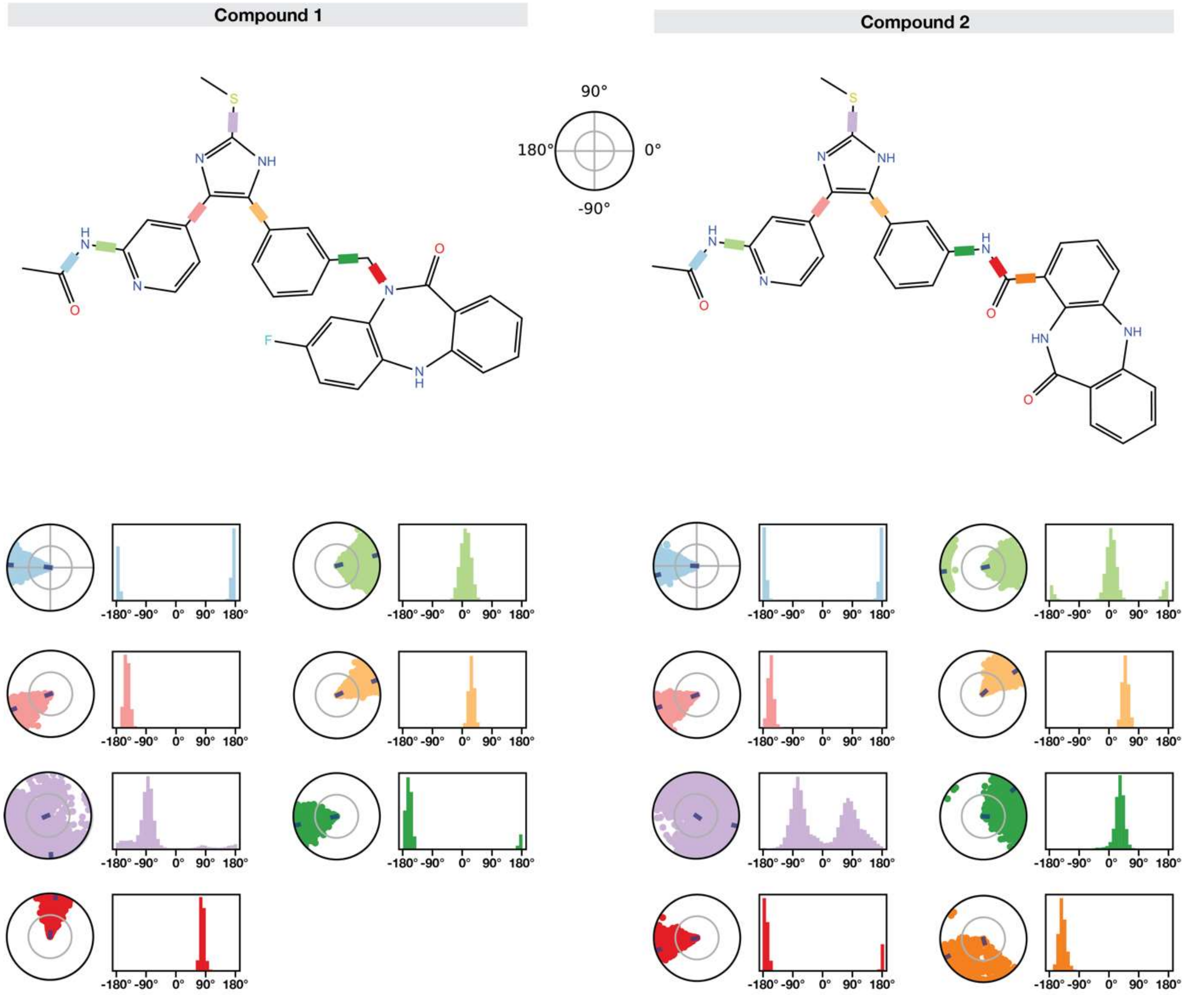


**Figure S12**: **Torsional profile of compound 1 and 2 derived from molecular dynamics simulations for compound 1 and 2.** The polar plots show the conformation of the as a function of time, where the radial coordinate is the simulation time, and the angular coordinate the torsional angle. The bar charts show the probability of the torsions as a function of angle. They represent the angle over the simulation time. The colour of the plot matches the colour coding of the rotatable bond on the compounds' structure. Torsional profile is shown for the merged MD trajectories (10μs/compound).

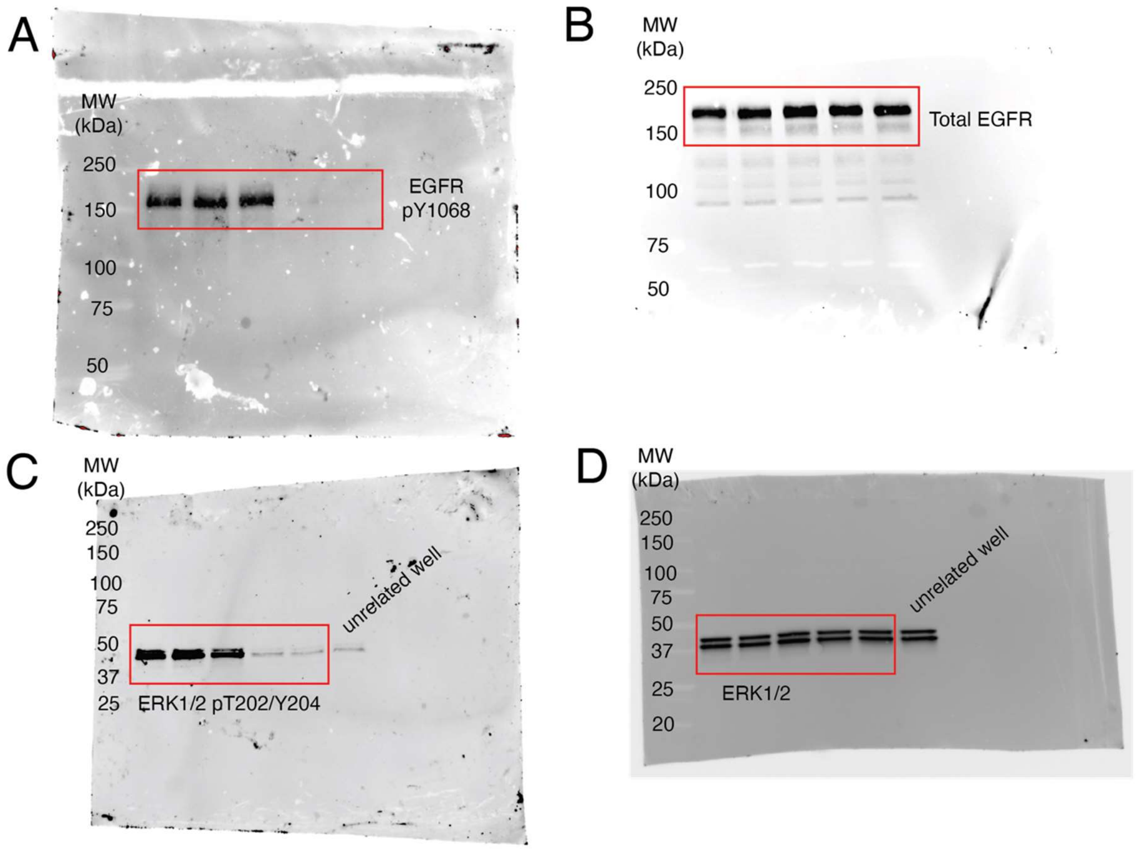


**Figure S13. Full uncropped Western blots of EGFR pY1068, total EGFR, ERK1/2 pY202/Y204, and total ERK 1/2 featured in Figure 7a.**

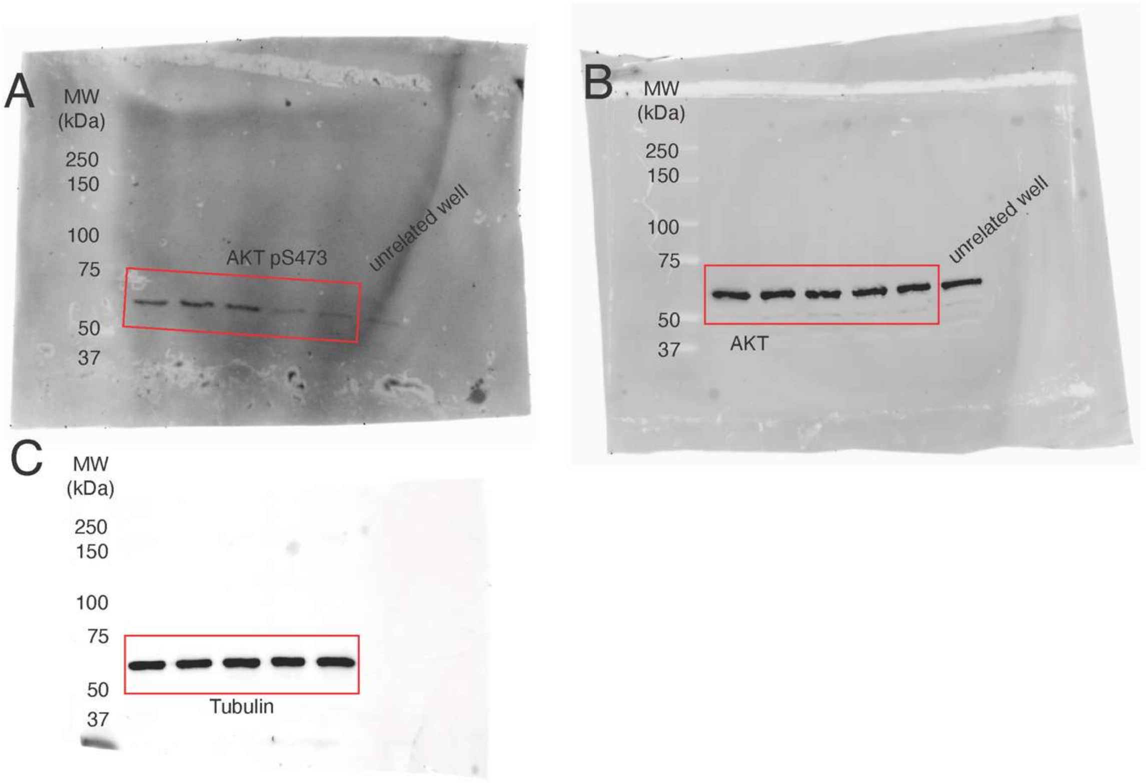


**Figure S14. Full uncropped Western blots of AKT pS473, total AKT, and Tubulin featured in Figure 7a.**

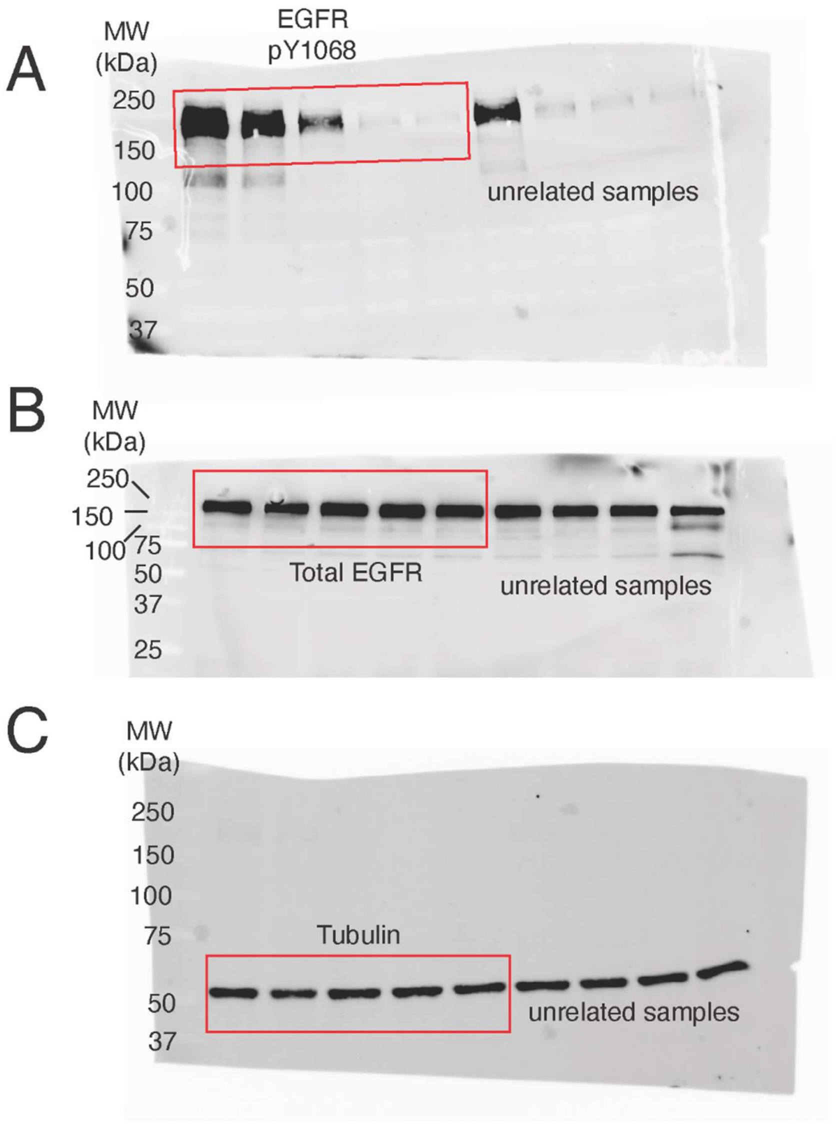


**Figure S15. Full uncropped Western blots featured in Figure 7b.**

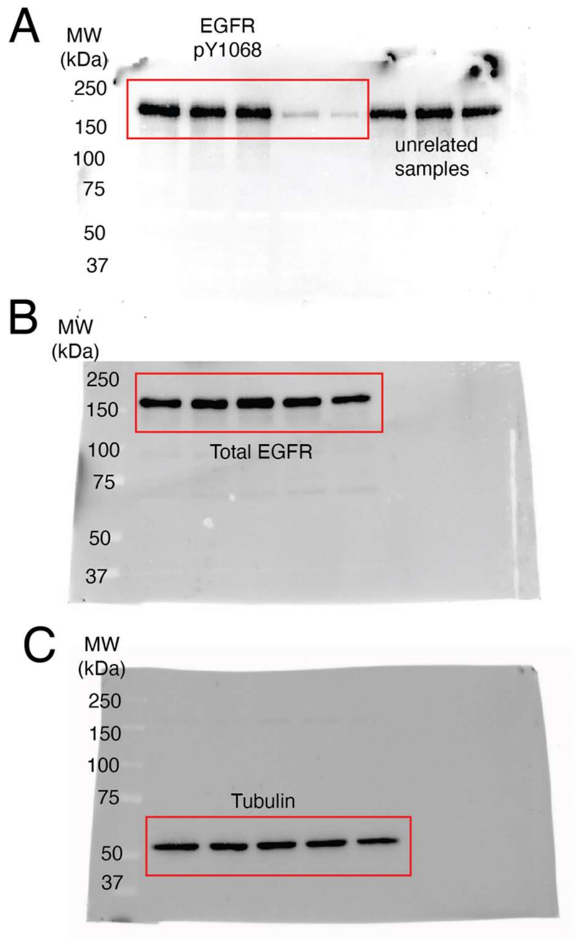


**Figure S16. Full uncropped Western blots corresponding to Figure 7c.**

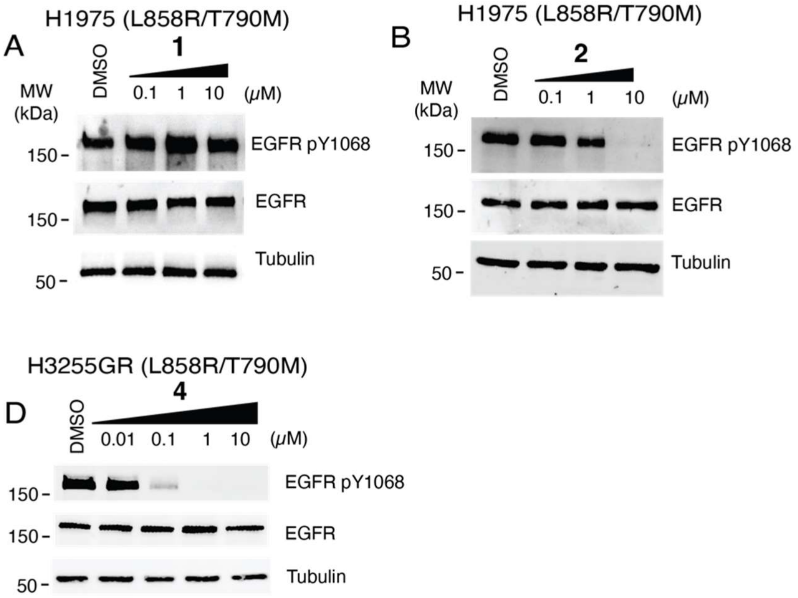


**Figure S17**. Variable impacts to EGFR phosphorylation in H1975 cells for reversible-binding compounds a) **1**, b) **2**, and c) **3**. d) Impact of compound **4** on EGFR phosphorylation in H3255GR cells. All cell line models were dosed with inhibitors for 6 hours. Western blots are representative of *N=3* independent experiments.

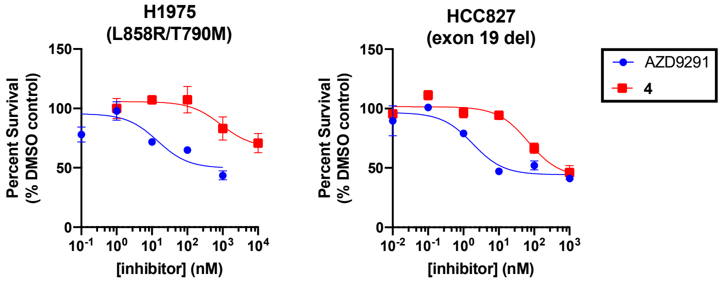


**Figure S18.** Antiproliferative activity of **4** against human derived cancer cell lines H1975 and HCC827 that harbor the LRTM and the exon19del mutations, respectively. Data shown are averages (error bars represent standard deviations) representative of *N=3* independent experiments each performed in triplicate.

**Table S5.** Cellular $EC_{50}$ values of **4** in Ba/F3 and human cancer cell line models.

| | H1975 (LR/TM) | HCC827 (exon19del) |
|---|---|---|
| **4** | 580 ± 300 | 140 ± 80 |
| AZD9291 | 23 ± 11 | 2.3 ± 1 |

Antiproliferative effects measured at 72 hours dosing with MTT reagent. Data represents the mean ± standard deviation of at least *N=3* independent experiments.

**Table S6:** Anti-proliferative activities ($EC_{50}$ values) of selected inhibitors on the proliferation of Ba/F3 cell lines of wild-type and mutant EGFR of at least *N=3* independent experiments.

| **EGFR $EC_{50}$ ± standard deviation (nM)** | | | | |
|---|---|---|---|---|
| **Compound** | **wt** | **LR** | **LR/TM** | **LR/TM/CS** |
| **2** | 5100 ± 2000 | 5300 ± 900 | > 10000 | 6500 ± 4000 |
| **4** | 950 ± 200 | 220 ± 80 | 770 ± 390 | > 10000 |
| **DDC4002**[a] | > 10,000 (9700 ± 500) [b] | > 10,000 (> 10,000) [b] | > 10,000 (1500 ± 400) [b] | > 10,000 (1200 ± 300) [b] |
| **LN2057** | 20 ± 6 [c] | <1[c,d] | 22 ± 1 [c] | 1600 ± 200[c] |
| **AZD9291** | 110 ± 40 [c] | 3.3 ± 0.6 [c] | 8 ± 0 [c] | 1200 ± 130[c] |

[a]Data from De Clercq and Heppner.[3] [b]Antiproliferative activity Ba/F3 + cetuximab. [c]Data from Wittlinger and Heppner.[4] [d]Values below the resolution limit of the assay.

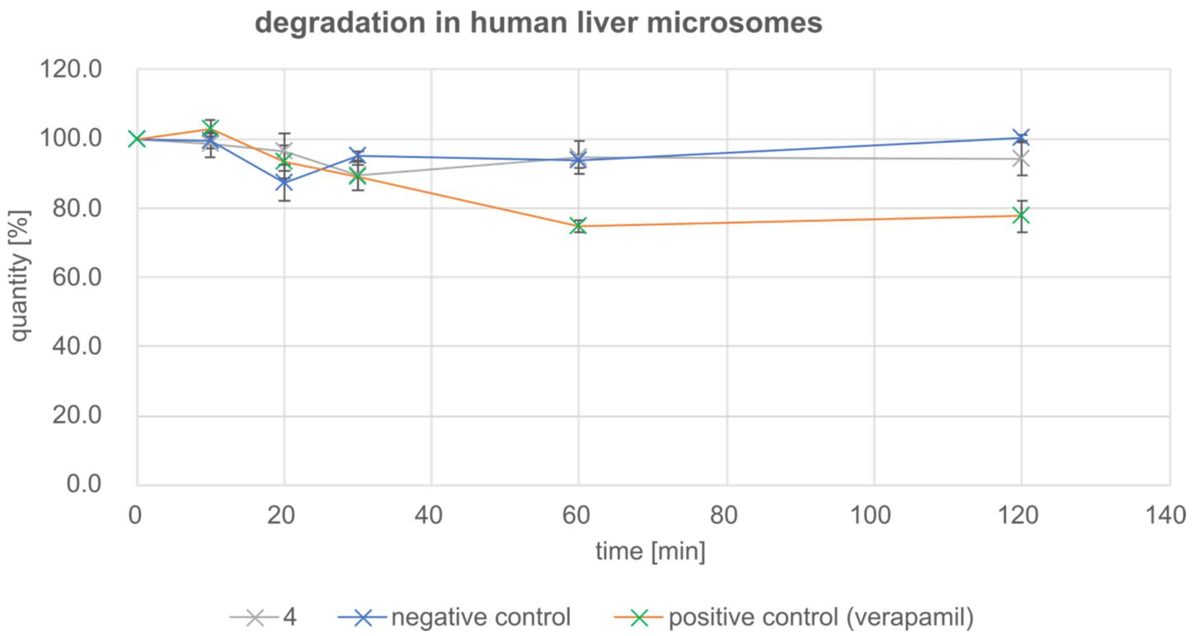


**Figure S19**: Metabolic stability determination of **4** in human liver microsomes, compared to a negative control containing bovine serum albumin instead of liver microsomes and a positive control using verapamil instead of compound.

**Table S7**: Data was obtained from KINOMEscan™ using 1 µM of compound **4** against 468 kinases. Eurofins DiscoverX Corporation (San Diego), CA 92121, USA. Results stated as percent of control (POC), where lower values are associated with lower Kd values.

| **Target** | **POC** |
|---|---|
| AAK1 | 100 |
| ABL1(E255K)-phosphorylated | 65 |
| ABL1(F317I)-nonphosphorylated | 40 |
| ABL1(F317I)-phosphorylated | 84 |
| ABL1(F317L)-nonphosphorylated | 37 |
| ABL1(F317L)-phosphorylated | 85 |
| ABL1(H396P)-nonphosphorylated | 97 |
| ABL1(H396P)-phosphorylated | 74 |
| ABL1(M351T)-phosphorylated | 67 |
| ABL1(Q252H)-nonphosphorylated | 100 |

| | |
|---|---|
| ABL1(Q252H)-phosphorylated | 90 |
| ABL1(T315I)-nonphosphorylated | 98 |
| ABL1(T315I)-phosphorylated | 100 |
| ABL1(Y253F)-phosphorylated | 72 |
| ABL1-nonphosphorylated | 100 |
| ABL1-phosphorylated | 80 |
| ABL2 | 92 |
| ACVR1 | 96 |
| ACVR1B | 85 |
| ACVR2A | 77 |
| ACVR2B | 72 |
| ACVRL1 | 75 |
| ADCK3 | 100 |
| ADCK4 | 85 |
| AKT1 | 89 |
| AKT2 | 91 |
| AKT3 | 100 |
| ALK | 100 |
| ALK(C1156Y) | 87 |
| ALK(L1196M) | 89 |
| AMPK-alpha1 | 81 |
| AMPK-alpha2 | 56 |
| ANKK1 | 87 |
| ARK5 | 100 |
| ASK1 | 100 |
| ASK2 | 100 |
| AURKA | 90 |
| AURKB | 100 |

| | |
|---|---|
| AURKC | 100 |
| AXL | 98 |
| BIKE | 100 |
| BLK | 1.7 |
| BMPR1A | 85 |
| BMPR1B | 100 |
| BMPR2 | 88 |
| BMX | 66 |
| BRAF | 90 |
| BRAF(V600E) | 100 |
| BRK | 96 |
| BRSK1 | 100 |
| BRSK2 | 93 |
| BTK | 54 |
| BUB1 | 100 |
| CAMK1 | 81 |
| CAMK1B | 94 |
| CAMK1D | 100 |
| CAMK1G | 100 |
| CAMK2A | 65 |
| CAMK2B | 72 |
| CAMK2D | 88 |
| CAMK2G | 100 |
| CAMK4 | 99 |
| CAMKK1 | 85 |
| CAMKK2 | 95 |
| CASK | 97 |
| CDC2L1 | 100 |

| | |
|---|---|
| CDC2L2 | 91 |
| CDC2L5 | 74 |
| CDK11 | 100 |
| CDK2 | 95 |
| CDK3 | 100 |
| CDK4 | 100 |
| CDK4-cyclinD1 | 94 |
| CDK4-cyclinD3 | 49 |
| CDK5 | 100 |
| CDK7 | 49 |
| CDK8 | 81 |
| CDK9 | 100 |
| CDKL1 | 100 |
| CDKL2 | 100 |
| CDKL3 | 100 |
| CDKL5 | 98 |
| CHEK1 | 100 |
| CHEK2 | 87 |
| CIT | 6.3 |
| CLK1 | 100 |
| CLK2 | 63 |
| CLK3 | 74 |
| CLK4 | 100 |
| CSF1R | 100 |
| CSF1R-autoinhibited | 69 |
| CSK | 14 |
| CSNK1A1 | 56 |
| CSNK1A1L | 91 |

| | |
|---|---|
| CSNK1D | 87 |
| CSNK1E | 88 |
| CSNK1G1 | 93 |
| CSNK1G2 | 91 |
| CSNK1G3 | 98 |
| CSNK2A1 | 100 |
| CSNK2A2 | 58 |
| CTK | 54 |
| DAPK1 | 86 |
| DAPK2 | 100 |
| DAPK3 | 62 |
| DCAMKL1 | 100 |
| DCAMKL2 | 100 |
| DCAMKL3 | 86 |
| DDR1 | 90 |
| DDR2 | 100 |
| DLK | 100 |
| DMPK | 45 |
| DMPK2 | 0 |
| DRAK1 | 84 |
| DRAK2 | 94 |
| DYRK1A | 100 |
| DYRK1B | 88 |
| DYRK2 | 75 |
| EGFR | 17 |
| EGFR(E746-A750del) | 13 |
| EGFR(G719C) | 6.5 |
| EGFR(G719S) | 13 |

| | |
|---|---|
| "EGFR(L747-E749del+A750P)" | 8 |
| "EGFR(L747-S752del+P753S)" | 16 |
| "EGFR(L747-T751del+Sins)" | 7.6 |
| EGFR(L858R) | 5 |
| "EGFR(L858R+T790M)" | 8.3 |
| EGFR(L861Q) | 2.7 |
| EGFR(S752-I759del) | 6 |
| EGFR(T790M) | 12 |
| EIF2AK1 | 75 |
| EPHA1 | 100 |
| EPHA2 | 100 |
| EPHA3 | 81 |
| EPHA4 | 95 |
| EPHA5 | 100 |
| EPHA6 | 90 |
| EPHA7 | 100 |
| EPHA8 | 98 |
| EPHB1 | 92 |
| EPHB2 | 100 |
| EPHB3 | 100 |
| EPHB4 | 100 |
| EPHB6 | 100 |
| ERBB2 | 18 |
| ERBB3 | 84 |
| ERBB4 | 4.9 |
| ERK1 | 99 |
| ERK2 | 100 |
| ERK3 | 100 |

| | |
|---|---|
| ERK4 | 100 |
| ERK5 | 100 |
| ERK8 | 100 |
| ERN1 | 74 |
| FAK | 99 |
| FER | 36 |
| FES | 28 |
| FGFR1 | 86 |
| FGFR2 | 100 |
| FGFR3 | 94 |
| FGFR3(G697C) | 100 |
| FGFR4 | 100 |
| FGR | 19 |
| FLT1 | 100 |
| FLT3 | 100 |
| FLT3(D835H) | 92 |
| FLT3(D835V) | 100 |
| FLT3(D835Y) | 96 |
| FLT3(ITD) | 100 |
| "FLT3(ITD+D835V)" | 100 |
| "FLT3(ITD+F691L)" | 79 |
| FLT3(K663Q) | 87 |
| FLT3(N841I) | 97 |
| FLT3(R834Q) | 100 |
| FLT3-autoinhibited | 100 |
| FLT4 | 100 |
| FRK | 20 |
| FYN | 64 |

| | |
|---|---|
| GAK | 16 |
| "GCN2(Kin.Dom.2+S808G)" | 100 |
| GRK1 | 94 |
| GRK2 | 100 |
| GRK3 | 100 |
| GRK4 | 100 |
| GRK7 | 99 |
| GSK3A | 65 |
| GSK3B | 100 |
| HASPIN | 100 |
| HCK | 14 |
| HIPK1 | 51 |
| HIPK2 | 100 |
| HIPK3 | 100 |
| HIPK4 | 92 |
| HPK1 | 45 |
| HUNK | 32 |
| ICK | 100 |
| IGF1R | 90 |
| IKK-alpha | 100 |
| IKK-beta | 88 |
| IKK-epsilon | 100 |
| INSR | 97 |
| INSRR | 87 |
| IRAK1 | 85 |
| IRAK3 | 39 |
| IRAK4 | 43 |
| ITK | 87 |

| | |
|---|---|
| JAK1(JH1domain-catalytic) | 91 |
| JAK1(JH2domain-pseudokinase) | 83 |
| JAK2(JH1domain-catalytic) | 86 |
| JAK3(JH1domain-catalytic) | 100 |
| JNK1 | 85 |
| JNK2 | 78 |
| JNK3 | 53 |
| KIT | 100 |
| KIT(A829P) | 56 |
| KIT(D816H) | 39 |
| KIT(D816V) | 94 |
| KIT(L576P) | 84 |
| KIT(V559D) | 100 |
| KIT(V559D+T670I) | 95 |
| KIT(V559D+V654A) | 93 |
| KIT-autoinhibited | 100 |
| LATS1 | 100 |
| LATS2 | 62 |
| LCK | 7.7 |
| LIMK1 | 79 |
| LIMK2 | 93 |
| LKB1 | 100 |
| LOK | 49 |
| LRRK2 | 93 |
| LRRK2(G2019S) | 100 |
| LTK | 48 |
| LYN | 22 |
| LZK | 90 |

| | |
|---|---|
| MAK | 89 |
| MAP3K1 | 100 |
| MAP3K15 | 74 |
| MAP3K2 | 100 |
| MAP3K3 | 51 |
| MAP3K4 | 100 |
| MAP4K2 | 36 |
| MAP4K3 | 9.5 |
| MAP4K4 | 6.1 |
| MAP4K5 | 2.5 |
| MAPKAPK2 | 100 |
| MAPKAPK5 | 100 |
| MARK1 | 92 |
| MARK2 | 85 |
| MARK3 | 100 |
| MARK4 | 92 |
| MAST1 | 100 |
| MEK1 | 100 |
| MEK2 | 100 |
| MEK3 | 100 |
| MEK4 | 100 |
| MEK5 | 41 |
| MEK6 | 73 |
| MELK | 100 |
| MERTK | 83 |
| MET | 69 |
| MET(M1250T) | 89 |
| MET(Y1235D) | 97 |

| | |
|---|---|
| MINK | 10 |
| MKK7 | 31 |
| MKNK1 | 100 |
| MKNK2 | 100 |
| MLCK | 86 |
| MLK1 | 86 |
| MLK2 | 97 |
| MLK3 | 97 |
| MRCKA | 0 |
| MRCKB | 1.7 |
| MST1 | 6 |
| MST1R | 85 |
| MST2 | 14 |
| MST3 | 73 |
| MST4 | 94 |
| MTOR | 100 |
| MUSK | 100 |
| MYLK | 84 |
| MYLK2 | 84 |
| MYLK4 | 95 |
| MYO3A | 14 |
| MYO3B | 31 |
| NDR1 | 73 |
| NDR2 | 77 |
| NEK1 | 90 |
| NEK10 | 86 |
| NEK11 | 100 |
| NEK2 | 98 |

| | |
|---|---|
| NEK3 | 100 |
| NEK4 | 100 |
| NEK5 | 100 |
| NEK6 | 100 |
| NEK7 | 86 |
| NEK9 | 83 |
| NIK | 100 |
| NIM1 | 78 |
| NLK | 48 |
| OSR1 | 89 |
| p38-alpha | 46 |
| p38-beta | 59 |
| p38-delta | 100 |
| p38-gamma | 100 |
| PAK1 | 100 |
| PAK2 | 100 |
| PAK3 | 70 |
| PAK4 | 89 |
| PAK6 | 93 |
| PAK7 | 77 |
| PCTK1 | 100 |
| PCTK2 | 100 |
| PCTK3 | 93 |
| PDGFRA | 69 |
| PDGFRB | 100 |
| PDPK1 | 84 |
| PFCDPK1(P.falciparum) | 87 |
| PFPK5(P.falciparum) | 100 |

| | |
|---|---|
| PFTAIRE2 | 84 |
| PFTK1 | 90 |
| PHKG1 | 96 |
| PHKG2 | 73 |
| PIK3C2B | 74 |
| PIK3C2G | 50 |
| PIK3CA | 70 |
| PIK3CA(C420R) | 66 |
| PIK3CA(E542K) | 100 |
| PIK3CA(E545A) | 100 |
| PIK3CA(E545K) | 100 |
| PIK3CA(H1047L) | 100 |
| PIK3CA(H1047Y) | 66 |
| PIK3CA(I800L) | 100 |
| PIK3CA(M1043I) | 55 |
| PIK3CA(Q546K) | 100 |
| PIK3CB | 90 |
| PIK3CD | 71 |
| PIK3CG | 65 |
| PIK4CB | 93 |
| PIKFYVE | 94 |
| PIM1 | 94 |
| PIM2 | 88 |
| PIM3 | 100 |
| PIP5K1A | 75 |
| PIP5K1C | 78 |
| PIP5K2B | 100 |
| PIP5K2C | 66 |

| | |
|---|---|
| PKAC-alpha | 100 |
| PKAC-beta | 98 |
| PKMYT1 | 99 |
| PKN1 | 88 |
| PKN2 | 100 |
| PKNB(M.tuberculosis) | 97 |
| PLK1 | 50 |
| PLK2 | 100 |
| PLK3 | 87 |
| PLK4 | 71 |
| PRKCD | 55 |
| PRKCE | 100 |
| PRKCH | 69 |
| PRKCI | 100 |
| PRKCQ | 18 |
| PRKD1 | 42 |
| PRKD2 | 62 |
| PRKD3 | 67 |
| PRKG1 | 100 |
| PRKG2 | 62 |
| PRKR | 87 |
| PRKX | 100 |
| PRP4 | 46 |
| PYK2 | 100 |
| QSK | 100 |
| RAF1 | 73 |
| RET | 99 |
| RET(M918T) | 99 |

| | |
|---|---|
| RET(V804L) | 99 |
| RET(V804M) | 91 |
| RIOK1 | 100 |
| RIOK2 | 83 |
| RIOK3 | 100 |
| RIPK1 | 100 |
| RIPK2 | 62 |
| RIPK4 | 100 |
| RIPK5 | 79 |
| ROCK1 | 100 |
| ROCK2 | 94 |
| ROS1 | 80 |
| RPS6KA4(Kin.Dom.1-N-terminal) | 96 |
| RPS6KA4(Kin.Dom.2-C-terminal) | 75 |
| RPS6KA5(Kin.Dom.1-N-terminal) | 83 |
| RPS6KA5(Kin.Dom.2-C-terminal) | 100 |
| RSK1(Kin.Dom.1-N-terminal) | 4.7 |
| RSK1(Kin.Dom.2-C-terminal) | 75 |
| RSK2(Kin.Dom.1-N-terminal) | 100 |
| RSK2(Kin.Dom.2-C-terminal) | 100 |
| RSK3(Kin.Dom.1-N-terminal) | 97 |
| RSK3(Kin.Dom.2-C-terminal) | 80 |
| RSK4(Kin.Dom.1-N-terminal) | 100 |
| RSK4(Kin.Dom.2-C-terminal) | 80 |
| S6K1 | 100 |
| SBK1 | 84 |
| SGK | 86 |
| SgK110 | 100 |

| | |
|---|---|
| SGK2 | 83 |
| SGK3 | 90 |
| SIK | 100 |
| SIK2 | 100 |
| SLK | 100 |
| SNARK | 100 |
| SNRK | 82 |
| SRC | 73 |
| SRMS | 61 |
| SRPK1 | 100 |
| SRPK2 | 100 |
| SRPK3 | 100 |
| STK16 | 100 |
| STK33 | 100 |
| STK35 | 69 |
| STK36 | 30 |
| STK39 | 85 |
| SYK | 63 |
| TAK1 | 95 |
| TAOK1 | 100 |
| TAOK2 | 100 |
| TAOK3 | 95 |
| TBK1 | 95 |
| TEC | 45 |
| TESK1 | 94 |
| TGFBR1 | 75 |
| TGFBR2 | 75 |
| TIE1 | 100 |

| | |
|---|---|
| TIE2 | 71 |
| TLK1 | 87 |
| TLK2 | 87 |
| TNIK | 33 |
| TNK1 | 22 |
| TNK2 | 3.8 |
| TNNI3K | 89 |
| TRKA | 77 |
| TRKB | 100 |
| TRKC | 87 |
| TRPM6 | 99 |
| TSSK1B | 95 |
| TSSK3 | 100 |
| TTK | 72 |
| TXK | 42 |
| TYK2(JH1domain-catalytic) | 91 |
| TYK2(JH2domain-pseudokinase) | 100 |
| TYRO3 | 32 |
| ULK1 | 100 |
| ULK2 | 80 |
| ULK3 | 100 |
| VEGFR2 | 100 |
| VPS34 | 84 |
| VRK2 | 100 |
| WEE1 | 85 |
| WEE2 | 98 |
| WNK1 | 100 |
| WNK2 | 62 |

| | |
|---|---|
| WNK3 | 78 |
| WNK4 | 85 |
| YANK1 | 84 |
| YANK2 | 63 |
| YANK3 | 96 |
| YES | 20 |
| YSK1 | 76 |
| YSK4 | 88 |
| ZAK | 87 |
| ZAP70 | 97 |

**Supplementary Note 1.**

**Preparation of 1**

***N*-(5-Fluoro-2-iodophenyl)-2-nitrobenzamide (S1)**

To begin, 917 mg (5.48 mmol) 2-nitrobenzoic acid was dissolved in 20 mL of DCM, and 0.4 ml of DMF was added to the mixture. 0.54 ml (6.33 mmol) of oxalyl chloride were added dropwise under gas formation and the mixture was stirred for 1 h at ambient temperature, whereupon the excess of oxalyl chloride was removed in vacuo. At the same time, 1.00 g (4.22 mmol) of 5-fluoro-2-iodoaniline was dissolved in 20 mL of DCM, 1.8 ml (12.87 mmol) of triethylamine was added, and the mixture was cooled down to 0 °C. The previously prepared acid chloride was dissolved in 10 mL of DCM and slowly added to the reaction mixture, whereupon the mixture was warmed to ambient temperature and stirred for 1 h. Brine was added to the reaction mixture and the aqueous layer was extracted with DCM. The combined organic layers were dried over $Na_2SO_4$, and solvents were removed in vacuo. Purification via flash chromatography ($SiO_2$; *n*-hexane/EtOAc 50:50) yielded 38% (611 mg, 1.58 mmol) of a light-yellow solid. $^1$H NMR (400 MHz, DMSO) δ 10.39 (s, 1H), 8.19 (d, *J* = 8.1 Hz, 1H), 7.97 – 7.89 (m, 2H), 7.84 – 7.75 (m, 2H), 7.40 (dd, *J* = 10.1, 2.7 Hz, 1H), 7.02 (td, *J* = 8.5, 2.9 Hz, 1H). $^{13}$C NMR (101 MHz, DMSO) δ 164.7, 162.0 (d, *J* = 245.1 Hz), 146.4, 140.5 (d, *J* = 10.3 Hz), 140.2 (d, *J* = 8.7 Hz), 134.1, 132.1, 131.2, 129.0, 124.3, 115.5 (d, *J* = 21.6 Hz), 114.2 (d, *J* = 24.2 Hz), 90.1. TLC-MS (ESI+): calcd. *m*/*z* 385.96 for $C_{13}H_8FIN_2O_3$, found 409.4 $[M + Na]^+$.

***N*-(3-Bromobenzyl)-*N*-(5-fluoro-2-iodophenyl)-2-nitro benzamide (S2)**

To begin, 582 mg (1.51 mmol) of **S1** was dissolved in 15 mL of THF under a nitrogen atmosphere, and the solution was cooled to 0 °C. To the solution was added 66 mg (1.66 mmol) of a 60% dispersion in oil of sodium hydride portionwise. To the stirred reaction mixture was added 414 mg (1.66 mmol) of 3-bromobenzyl bromide portionwise. After full addition, the mixture was warmed to ambient temperature and stirred overnight until complete conversion. Brine was added, and the organic layer was separated. The aqueous phase was extracted with EtOAc twice, and the combined organic layers were dried over $Na_2SO_4$. After removal of solvents the product was purified via flash chromatography ($SiO_2$; *n*-hexane/EtOAc 50:50). Yield: 91% (765 mg, 1.38 mmol) of a yellowish solid. $^1$H NMR (400 MHz, $CDCl_3$) δ 7.91 – 7.86 (m, 1H), 7.68 – 7.63 (m, 1H), 7.57 (d, *J* = 7.6 Hz, 1H), 7.40 (t, *J* = 7.5 Hz, 1H), 7.37 – 7.25 (m, 4H), 7.18 – 7.12 (m, 1H), 6.58 – 6.52 (m, 1H), 6.51 – 6.45 (m, 1H), 5.80 (d, *J* = 14.4 Hz, 1H; $CH_2$), 4.17 (d, *J* = 14.4 Hz, 1H; $CH_2$). $^{13}$C NMR (101 MHz, $CDCl_3$) δ 166.6, 162.6 (d, *J* = 252.1 Hz), 145.9, 143.9 (d, *J* = 9.7 Hz), 141.0 (d, *J* = 8.5 Hz), 137.6, 133.8, 132.7, 132.1, 131.4, 130.4, 130.3, 128.5, 127.7, 124.6, 122.5, 118.9 (d, *J* = 23.1 Hz), 118.0 (d, *J* = 21.6 Hz), 92.8 (d, *J* = 4.0 Hz), 50.9. TLC-MS (ESI+): calcd. *m*/*z* 553.91 for $C_{20}H_{13}BrFIN_2O_3$, found 577.5/579.5 $[M + Na]^+$.

**2-Amino-*N*-(3-bromobenzyl)-*N*-(5-fluoro-2-iodophenyl) benzamide (S3)**

700 mg (1.26 mmol) of **S2**, 352 mg (6.30 mmol) of iron powder, and 674 mg (3.40 mmol) of ammonium chloride were suspended in a mixture of THF/MeOH/$H_2O$ (5:2:1, 12.5 mL). The resulting mixture was vigorously stirred at 50 °C for 1 h. Then, 72 µL of acetic acid was added and the mixture was stirred for another 1 h at 50 °C. Thereupon, the reaction mixture was cooled to

ambient temperature and filtered through a pad of celite. To the filtrate, water was added and the aqueous phase was extracted with EtOAC. The separated organic layer was then washed three times with an aqueous saturated $NaHCO_3$ solution. The organic layer was dried over $Na_2SO_4$, filtered and concentrated in vacuo. Obtaining 666 mg (quant., 1.26 mmol) of a light-yellow solid. The product was used as crude without further purification in the next step. TLC-MS (ESI+): calcd. *m*/*z* 523.94 for $C_{20}H_{15}BrFIN_2O$, found 547.5/549.5 $[M + Na]^+$.

**10-(3-Bromobenzyl)-8-fluoro-5,10-dihydro-11*H*-dibenzo[b,e][1,4]diazepin-11-one (S4)**

660 mg (1.26 mmol) of **S3**, 48 mg (0.25 mmol) of copper(I) iodide, and 434 mg (3.14 mmol) of $K_2CO_3$ were suspended in 2 ml of DMSO and the resulting reaction mixture was stirred at 135 °C for 2 h. After cooling down to ambient temperature, the mixture was diluted with an excess of EtOAc and washed three times with water. The organic layer was dried over $Na_2SO_4$, filtered and concentrated in vacuo. The crude product was purified via flash chromatography ($SiO_2$; *n*-hexane/EtOAc 65:35) to yield 76% (380 mg, 0.96 mmol) of a light-yellow solid. $^1H$ NMR (400 MHz, DMSO) δ 7.96 (s, 1H), 7.65 (d, *J* = 7.8 Hz, 1H), 7.52 (s, 1H), 7.41 – 7.35 (m, 2H), 7.32 – 7.23 (m, 3H), 7.16 – 7.11 (m, 1H), 7.09 (d, *J* = 7.6 Hz, 1H), 7.02 – 6.97 (m, 1H), 6.94 – 6.89 (m, 1H), 5.29 (s, 2H). $^{13}C$ NMR (101 MHz, DMSO) δ 167.9, 158.3 (d, *J* = 238.9 Hz), 152.1, 141.8, 140.2, 134.2 (d, *J* = 10.0 Hz), 132.8, 132.1, 130.5, 129.8, 129.3, 125.7, 124.2, 121.8, 121.7, 121.6 (d, *J* = 9.3 Hz), 118.8, 112.7 (d, *J* = 22.3 Hz), 110.9 (d, *J* = 25.3 Hz), 50.9. TLC-MS (ESI+): calcd. *m*/*z* 396.03 for $C_{20}H_{14}BrFN_2O$, found 451.4/453.4 $[M + Na + MeOH]^+$.

**8-Fluoro-10-(3-(4,4,5,5-tetramethyl-1,3,2-dioxaborolan-2-yl)benzyl)-5,10-dihydro-11*H*-dibenzo[b,e][1,4]diazepin-11-one (S5)**

To begin, 365 mg (0.92 mmol) of **S4**, 231 mg (0.91 mmol) of bis(pinacolato)diboron and 271 mg (2.76 mmol) of KOAc were suspended in 10 mL of 1,4-dioxane under an argon atmosphere. The suspension was degassed with three cycles of evacuation and backfilling with argon, followed by the addition of 34 mg (5 mol%) of $Pd(dppf)Cl_2$ and another three cycles of degassing. The reaction mixture was heated to 90 °C and stirred overnight. Thereupon, the reaction mixture was cooled down to ambient temperature and filtered through a pad of celite. The filtrate was concentrated and 410 mg (quant., 0.92 mmol) of a crude, brown oil was isolated and used without further purification in the next step. TLC-MS (ESI+): calcd. *m*/*z* 444.20 for $C_{26}H_{26}BFN_2O_3$, found 467.6 $[M + Na]^+$.

**8-Fluoro-10-(3-(2-(methylthio)-1-((2-(trimethylsilyl)ethoxy)methyl)-1*H*-imidazol-4-yl)benzyl)-5,10-dihydro-11*H*-dibenzo[b,e][1,4]diazepin-11-one (S6)**

To begin, 407 mg (0.92 mmol) of **S5**, 444 mg (1.37 mmol) of 4-bromo-2-(methylthio)-1-((2-(trimethylsilyl)ethoxy)methyl)-1*H*-imidazole (prepared as previously described[4]), and 976 mg (3.66 mmol) of $K_3PO_4$ trihydrate were suspended in 9 mL of 1,4-dioxane and 2.5 mL of demineralized water. The solution was degassed with three cycles of evacuation and backfilling with argon, followed by the addition of 52 mg (10 mol%) of P(*t*-Bu)$_3$ Pd G3 and another three cycles of evacuation and argon backfilling. The reaction mixture was warmed to 50 °C and stirred overnight. After cooling to ambient temperature, brine was added to the mixture and the aqueous phase was extracted several times with EtOAc. Combined organic layers were dried over $Na_2SO_4$, filtered, and evaporated to dryness. The crude product was purified via flash chromatography ($SiO_2$; *n*-hexane/EtOAc 70:30) to obtain a yellow oil in a 70% yield (361 mg, 0.64 mmol). $^1H$ NMR

(400 MHz, DMSO) δ 7.88 (s, 1H), 7.77 (s, 1H), 7.72 (s, 1H), 7.68 – 7.65 (m, 1H), 7.59 – 7.55 (m, 1H), 7.39 – 7.35 (m, 1H), 7.29 – 7.27 (m, 1H), 7.25 (dd, *J* = 7.5, 3.1 Hz, 1H), 7.17 – 7.11 (m, 2H), 7.10 – 7.07 (m, 1H), 7.02 – 6.98 (m, 1H), 6.91 – 6.86 (m, 1H), 5.30 (s, 2H), 5.28 (s, 2H), 3.55 – 3.50 (m, 2H), 2.58 (s, 3H), 0.88 – 0.84 (m, 2H), -0.04 (s, 9H). $^{13}$C NMR (101 MHz, DMSO) δ 167.9, 159.4, 157.0, 152.1, 143.1, 141.7, 141.7, 140.3, 137.6, 134.6, 134.5, 133.9, 132.6, 132.1, 128.6, 124.7, 124.4, 122.8, 122.6, 121.7, 121.6, 121.5, 118.8, 118.4, 112.6, 112.3, 110.8, 110.6, 73.5, 65.5, 51.7, 17.1, 15.8, -1.4. (With residues of pinacol). TLC-MS (ESI+): calcd. *m*/*z* 560.21 for $C_{30}H_{33}FN_4O_2SSi$, found 584.0 $[M + Na]^+$.

**10-(3-(5-Bromo-2-(methylthio)-1-((2-(trimethylsilyl)ethoxy)methyl)-1*H*-imidazol-4-yl)benzyl)-8-fluoro-5,10-dihydro-11*H*-dibenzo[b,e][1,4]diazepin-11-one (S7)**

To begin, 323 mg (0.58 mmol) of **S6** was dissolved in 10 mL of ACN under an argon atmosphere and the solution was cooled to -30 °C. Then, 92 mg (0.52 mmol) of *N*-bromosuccinimide dissolved in 10 mL of ACN was added dropwise to the solution while the temperature was maintained at -30 °C. The reaction mixture was stirred for 1 h at -30 °C and then slowly warmed up to ambient temperature. The reaction mixture was quenched by the addition of an aqueous saturated $Na_2SO_3$ solution, and the aqueous phase was extracted several times with EtOAc. The organic layers were dried over $Na_2SO_4$, filtered, and evaporated to dryness. The crude product was purified via flash chromatography ($SiO_2$; *n*-hexane/EtOAc 70:30) to obtain a yellowish solid in a 70% yield (260 mg, 0.41 mmol). $^1$H NMR (400 MHz, DMSO) δ 7.87 (s, 1H), 7.85 (s, 1H), 7.75 (d, *J* = 7.6 Hz, 1H), 7.67 (d, *J* = 7.7 Hz, 1H), 7.39 – 7.34 (m, 2H), 7.29 – 7.26 (m, 1H), 7.24 (s, 1H), 7.14 – 7.10 (m, 1H), 7.09 – 7.06 (m, 1H), 7.01 – 6.96 (m, 1H), 6.92 – 6.87 (m, 1H), 5.31 (s, 2H), 5.30 (s, 2H), 3.58 (t, *J* = 7.8 Hz, 2H), 2.60 (s, 3H), 0.87 (t, *J* = 7.9 Hz, 2H), -0.04 (s, 9H). $^{13}$C NMR (101 MHz, DMSO) δ 167.8, 159.4, 157.0, 152.1, 144.9, 141.6, 141.5, 137.8, 137.6, 134.6, 134.5, 132.7, 132.6, 132.1, 128.5, 125.7, 124.7, 124.5, 124.2, 121.6, 121.5, 118.7, 112.5, 112.3, 110.7, 110.5, 100.6, 73.6, 65.9, 51.8, 17.2, 15.5, -1.4. TLC-MS (ESI+): calcd. *m*/*z* 638.12 for $C_{30}H_{32}BrFN_4O_2SSi$, found 660.6/662.5 $[M + Na]^+$.

***N*-(4-(4-(3-((8-Fluoro-11-oxo-5,11-dihydro-10*H*-dibenzo[b,e][1,4]diazepin-10-yl)methyl)phenyl)-2-(methylthio)-1-((2-(trimethylsilyl)ethoxy)methyl)-1*H*-imidazol-5-yl)pyridin-2-yl)acetamide (S8)**

To begin, 260 mg (0.41 mmol) of **S7**, 197 mg (0.61 mmol) of *N*-(4-(4,4,5,5-tetramethyl-1,3,2-dioxaborolan-2-yl)pyridin-2-yl)acetamide, and 433 mg (1.63 mmol) of $K_3PO_4$ trihydrate were suspended in 4 mL of 1,4-dioxane and 1 mL of demineralized water. The solution was degassed with three cycles of evacuation and backfilling with argon, followed by the addition of 23 mg (10 mol%) of P(*t*-Bu)$_3$ Pd G3 and another three cycles of evacuation and argon backfilling. The reaction mixture was warmed to 50 °C and stirred overnight. After cooling down to ambient temperature, brine was added to the mixture and the aqueous phase was extracted several times with EtOAc. Combined organic layers were dried over $Na_2SO_4$, filtered, and evaporated to dryness. The crude product was purified via flash chromatography ($SiO_2$; *n*-hexane/EtOAc 50:50) to obtain a yellow solid in a 64% yield (180 mg, 0.26 mmol). $^1$H NMR (400 MHz, DMSO) δ 10.52 (s, 1H), 8.11 (d, *J* = 5.0 Hz, 1H), 8.08 (s, 1H), 7.82 (s, 1H), 7.60 (d, *J* = 7.2 Hz, 1H), 7.46 (s, 1H), 7.37 (t, *J* = 7.0 Hz, 1H), 7.23 – 7.18 (m, 2H), 7.16 – 7.13 (m, 1H), 7.13 – 7.09 (m, 1H), 7.08 – 7.03 (m, 2H), 7.02 – 6.98 (m, 1H), 6.97 – 6.95 (m, 1H), 6.89 (td, *J* = 8.5, 2.6 Hz, 1H), 5.15 (s, 2H), 5.08 (s, 2H), 3.37

– 3.33 (m, 2H), 2.66 (s, 3H), 2.05 (s, 3H), 0.79 – 0.75 (m, 2H), -0.09 (s, 9H). $^{13}$C NMR (101 MHz, DMSO) δ 169.2, 167.7, 159.3, 157.0, 152.6, 151.9, 148.3, 144.9, 141.28, 141.26, 139.5, 138.1, 137.5, 134.7, 134.6, 133.7, 132.6, 132.2, 128.3, 127.9, 125.02, 124.96, 124.3, 121.6, 121.5, 121.4, 120.4, 118.7, 114.2, 112.5, 112.3, 110.4, 110.2, 72.5, 65.5, 52.1, 23.8, 17.1, 15.5, -1.5. TLC-MS (ESI+): calcd. *m*/*z* 694.26 for $C_{37}H_{39}FN_6O_3SSi$, found 717.0 $[M + Na]^+$.

**_N_-(4-(4-(3-((8-Fluoro-11-oxo-5,11-dihydro-10H-dibenzo[b,e][1,4]diazepin-10-yl)methyl)phenyl)-2-(methylthio)-1_H_-imidazol-5-yl)pyridin-2-yl)acetamide (1)**

40 mg (0.06 mmol) of **S8** was dissolved in 3 mL of a mixture of TFA/DCM 33% v/v. The solution was stirred overnight at ambient temperature. After quenching the reaction mixture with a saturated aqueous $NaHCO_3$ solution, the aqueous layer was extracted several times with EtOAc. The combined organic layers were dried over $Na_2SO_4$, filtered, and the solvents removed in vacuo. The crude product was purified via flash chromatography ($SiO_2$; *n*-hexane/EtOAc/MeOH 10:85:5) to give the pure product as a white solid in a 62% yield (20 mg, 0.04 mmol). As mixture of tautomers: 1H NMR (400 MHz, DMSO) δ 12.82 – 12.62 (m, 1H), 10.46 – 10.23 (m, 1H), 8.35 – 7.93 (m, 2H), 7.84 (s, 1H), 7.57 (d, *J* = 7.6 Hz, 1H), 7.49 – 7.42 (m, 1H), 7.41 – 7.23 (m, 4H), 7.18 (d, *J* = 9.2 Hz, 1H), 7.12 – 7.02 (m, 2H), 6.96 (t, *J* = 7.5 Hz, 1H), 6.93 – 6.83 (m, 2H), 5.27 (s, 2H), 2.61 (s, 3H), 2.05 (s, 3H). HRMS (ESI): exact mass calcd. for $C_{31}H_{25}FN_6O_2S$ $[M + H]^+$: 565.18165, found: 565.18227.

**Preparation of 2-ethyl-imidazole precursors**

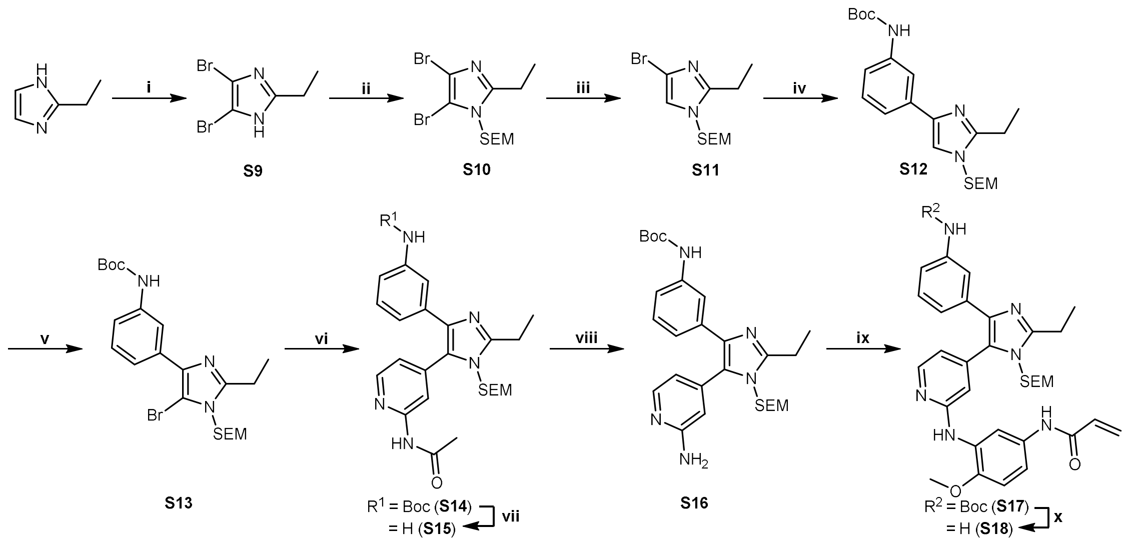


**Scheme S2: Synthesis of 2-ethyl-imidazole precursors** - Reagents and conditions are as follows: i) $Br_2$, $KHCO_3$, DMF, 70 °C, 76%; ii) SEM-Cl, NaH (60% dispersion in mineral oil), THF, 0 °C, 89%; iii) *n*-BuLi, THF, -80 °C, 99%; iv) 3-(*N*-Boc-amino)phenylboronic acid, $K_3PO_4$ trihydrate, P(*t*-Bu)$_3$ Pd G3, 1,4-dioxane/$H_2O$, 50 °C, 89%; v) NBS, ACN, -35 °C, 93%; vi) *N*-(4-(4,4,5,5-tetramethyl-1,3,2-dioxaborolan-2-yl)pyridin-2-yl)acetamide,, $K_3PO_4$ trihydrate, P(*t*-Bu)$_3$ Pd G3, 1,4-dioxane/$H_2O$, 55 °C, 80%; vii) 5% TFA in DCM, rt, 68%; viii) 3 N NaOH (aq), MeOH, 55 °C, 99%; ix) *N*-(3-bromo-4-methoxyphenyl)acrylamide, $Cs_2CO_3$, BrettPhos Pd G3, 1,4-dioxane/*t*-BuOH, rf, 87%; x) 7.5 % TFA in DCM, rt, 61%. With adaptions of previously described conditions – Wittlinger and Heppner et al. [4]

**4,5-Dibromo-2-ethyl-1*H*-imidazole (S9)**

To begin, 10.04 g (104.4 mmol) of 2-ethylimidazole was dissolved in 50 mL of DMF and 31.37 g (313.3 mmol) of $KHCO_3$ was added. The suspension was cooled down to 0 °C and 11 mL (214.1 mmol) of bromine was added dropwise under exothermic reaction. After complete addition, the reaction mixture was warmed up to 70 °C and stirred overnight. After cooling down to 0 °C, 150 mL of iced water was added, and the mixture was stirred for several minutes under formation of a yellow precipitate. The solid was filtered, washed with water, and dried in the oven. Yield: 20.16 g (76%, 79.41 mmol) as an off-white solid. $^1$H NMR (400 MHz, $CDCl_3$) δ 11.90 (s, 1H), 2.79 (q, *J* = 7.5 Hz, 2H), 1.29 (t, *J* = 7.5 Hz, 3H). $^{13}$C NMR (101 MHz, $CDCl_3$) δ 151.6, 106.8, 22.4, 12.7. [adapted from PATENT US6271380 B1 Gilligan, Paul Joseph; Bakthavatchalam, Rajagopal][5]

**4,5-Dibromo-2-ethyl-1-((2-(trimethylsilyl)ethoxy)methyl)-1*H*-imidazole (S10)**

To begin, 11.24 g (44.26 mmol) of **S9** was dissolved in 60 mL of THF under a nitrogen atmosphere, and the solution was cooled to 0 °C. To the solution was added 2.04 g (50.9 mmol) of a 60% dispersion in oil of sodium hydride portionwise while maintaining the temperature under 0 °C. To the stirred reaction mixture was added 8.38 mL (47.4 mmol) of SEM-Cl dissolved in 30 mL of THF dropwise while the temperature was maintained under 10 °C. After the full addition, the

mixture was warmed up to ambient temperature and stirred overnight. To the mixture was added brine and the aqueous phase was extracted several times with DCM. The combined organic layers were dried over $Na_2SO_4$, filtered, and the solvents removed in vacuo. The crude product was purified via flash chromatography ($SiO_2$; isocratic, *n*-hexane/EtOAc 95:5) to give the product as colorless oil in 89% yield (15.12 g, 39.36 mmol). $^1$H NMR (400 MHz, DMSO) δ 5.29 (s, 2H), 3.56 – 3.50 (m, 2H), 2.73 (q, *J* = 7.5 Hz, 2H), 1.19 (t, *J* = 7.5 Hz, 3H), 0.88 – 0.83 (m, 2H), -0.04 (s, 9H). $^{13}$C NMR (101 MHz, DMSO) δ 152.2, 115.9, 103.9, 74.4, 66.5, 21.1, 18.1, 12.3, -0.5.

**4-Bromo-2-ethyl-1-((2-(trimethylsilyl)ethoxy)methyl)-1*H*-imidazole (S11)**

To begin, 10.0 g (26.0 mmol) of **S10** was dissolved in 60 mL of THF under an argon atmosphere, and the solution was cooled to -80 °C. To the solution was added 10.4 mL (26.0 mmol) of a 2.5 M *n*-BuLi in *n*-hexane solution dropwise via syringe while the temperature was maintained at -80 °C. After full addition, the reaction mixture was stirred for 15 minutes, whereupon MeOH was added, and the mixture was warmed up to ambient temperature. To the mixture was added brine, and the organic layer was separated. The aqueous phase was extracted several times with EtOAc. The combined organic layers were dried over $Na_2SO_4$, and the solvent was removed in vacuo. The product was obtained as a yellow oil (99%, 7.90 g, 25.9 mmol) and was used without further purification in the next step. $^1$H NMR (400 MHz, DMSO) δ 7.32 (s, 1H), 5.24 (s, 2H), 3.50 – 3.45 (m, 2H), 2.65 (q, *J* = 7.5 Hz, 2H), 1.18 (t, *J* = 7.5 Hz, 3H), 0.86 – 0.81 (m, 2H), -0.04 (s, 9H). $^{13}$C NMR (101 MHz, DMSO) δ 149.8, 119.4, 112.2, 74.1, 65.3, 19.3, 17.1, 11.9, -1.4.

***tert*-Butyl (3-(2-ethyl-1-((2-(trimethylsilyl)ethoxy)methyl)-1*H*-imidazol-4-yl)phenyl) carbamate (S12)**

To begin, 2.50 g (8.19 mmol) of **S11**, 2.14 g (9.01 mmol) of 3-(*N*-Boc-amino)phenylboronic acid, and 8.72 g (32.8 mmol) of $K_3PO_4$ trihydrate were dissolved in 55 mL of 1,4-dioxane and 15 mL of demineralized water. The solution was degassed with three cycles of evacuation and backfilling with argon. To the solution was added 70 mg (1.5 mol%) of P(*t*-Bu)$_3$ Pd G3, and another three cycles of evacuation and argon backfilling were carried out. The reaction mixture was warmed to 50 °C and stirred overnight. After cooling to ambient temperature, brine was added and the aqueous phase was extracted several times with EtOAc. The combined organic layers were dried over $Na_2SO_4$, filtered, and evaporated to dryness. The crude product was purified via flash chromatography ($SiO_2$; *n*-hexane/EtOAc 70:30) to obtain a yellow solid in 89% yield (3.05 g, 7.30 mmol). $^1$H NMR (400 MHz, DMSO) δ 9.29 (s, 1H), 7.94 (s, 1H), 7.53 (s, 1H), 7.33 – 7.29 (m, 1H), 7.23 – 7.16 (m, 2H), 5.29 (s, 2H), 3.55 – 3.49 (m, 2H), 2.71 (q, *J* = 7.5 Hz, 2H), 1.48 (s, 9H), 1.25 (t, *J* = 7.5 Hz, 3H), 0.88 – 0.83 (m, 2H), -0.04 (s, 9H). $^{13}$C NMR (101 MHz, DMSO) δ 152.8, 149.7, 139.7, 138.3, 134.9, 128.6, 118.2, 116.5, 116.2, 114.1, 78.8, 74.2, 65.1, 28.1, 19.4, 17.2, 12.4, -1.7. TLC-MS (ESI+): calcd. *m*/*z* 417.24 for $C_{22}H_{35}N_3O_3Si$, found 418.5 $[M + H]^+$.

***tert*-Butyl (3-(5-bromo-2-ethyl-1-((2-(trimethylsilyl)ethoxy)methyl)-1*H*-imidazol-4-yl) phenyl)carbamate (S13)**

To begin, 2.90 g (6.94 mmol) of **S12** was dissolved in 50 mL of ACN under an argon atmosphere and the solution was cooled to -30 °C. Then, 1.24 g (6.94 mmol) of *N*-bromosuccinimide dissolved in 20 mL of ACN was added dropwise to the solution, while the temperature was maintained at -30 °C. The reaction mixture was stirred for 1 h at -30 °C and then slowly warmed to ambient

temperature. The reaction mixture was quenched by the addition of an aqueous saturated $Na_2SO_3$ solution, and the aqueous phase was extracted several times with EtOAc. The organic layers were dried over $Na_2SO_4$, filtered, and evaporated to dryness. The crude product was purified via flash chromatography ($SiO_2$; *n*-hexane/EtOAc 70:30) to obtain a light-yellow oil in 93% yield (3.19 g, 6.42 mmol). $^1H$ NMR (400 MHz, DMSO) δ 9.38 (s, 1H), 8.04 (s, 1H), 7.49 (d, *J* = 7.7 Hz, 1H), 7.38 (d, *J* = 7.8 Hz, 1H), 7.27 (t, *J* = 7.9 Hz, 1H), 5.33 (s, 2H), 3.58 (t, *J* = 8.0 Hz, 2H), 2.78 (q, *J* = 7.4 Hz, 2H), 1.48 (s, 9H), 1.26 (t, *J* = 7.5 Hz, 3H), 0.88 (t, *J* = 8.0 Hz, 2H), -0.03 (s, 9H). $^{13}C$ NMR (101 MHz, DMSO) δ 152.8, 150.9, 139. 6, 135.9, 133.6, 128.4, 120.0, 116.9, 116.3, 99.2, 78.9, 72.7, 65.4, 28.1, 20.2, 17.3, 11.8, -1.4. TLC-MS (ESI+): calcd. *m*/*z* 495.16 for $C_{22}H_{34}BrN_3O_3Si$, found 517.7/519.7 $[M + Na]^+$.

***tert*-Butyl (3-(5-(2-acetamidopyridin-4-yl)-2-ethyl-1-((2-(trimethylsilyl)ethoxy)methyl)-1*H*-imidazol-4-yl)phenyl) carbamate (S14)**

To begin, 2.70 g (5.44 mmol) of **S13**, 2.00 g (7.61 mmol) of *N*-(4-(4,4,5,5-tetramethyl-1,3,2-dioxaborolan-2-yl)pyridin-2-yl)acetamide, and 5.79 g (21.8 mmol) of $K_3PO_4$ trihydrate were dissolved in 55 mL of 1,4-dioxane and 15 mL of demineralized water. The solution was degassed with three cycles of evacuation and backfilling with argon. To the solution was added 50 mg (1.5 mol%) of $P(t\text{-}Bu)_3$ Pd G3, and another three cycles of evacuation and argon backfilling were carried out. The reaction mixture was warmed to 55 °C and stirred overnight. After cooling to ambient temperature, brine was added and the aqueous phase was extracted several times with EtOAc. The combined organic layers were dried over $Na_2SO_4$, filtered, and evaporated to dryness. The crude product was purified via flash chromatography ($SiO_2$; *n*-hexane/EtOAc 40:60) to obtain an off-white solid in 80% yield (2.40 g, 4.35 mmol). $^1H$ NMR (400 MHz, DMSO) δ 10.61 (s, 1H), 9.24 (s, 1H), 8.37 – 8.32 (m, 1H), 8.10 (s, 1H), 7.71 – 7.65 (m, 1H), 7.25 (d, *J* = 7.7 Hz, 1H), 7.05 (t, *J* = 7.9 Hz, 1H), 6.98 (dd, *J* = 5.1, 1.5 Hz, 1H), 6.87 – 6.82 (m, 1H), 5.10 (s, 2H), 3.33 – 3.29 (m, 2H), 2.80 (q, *J* = 7.5 Hz, 2H), 2.07 (s, 3H), 1.45 (s, 9H), 1.32 (t, *J* = 7.5 Hz, 3H), 0.79 – 0.74 (m, 2H), -0.09 (s, 9H). $^{13}C$ NMR (101 MHz, DMSO) δ 169.2, 152.69, 152.67, 150.5, 148.4, 140.3, 139.5, 136.5, 134.6, 128.2, 126.0, 120.8, 120.5, 116.9, 116.5, 114.6, 78.8, 71.8, 65.1, 28.1, 23.88, 19.8, 17.2, 12.1, -1.5. (With residues of pinacol). TLC-MS (ESI+): calcd. *m*/*z* 551.29 for $C_{29}H_{41}N_5O_4Si$, found 574.1 $[M + Na]^+$.

***N*-(4-(4-(3-Aminophenyl)-2-ethyl-1-((2-(trimethylsilyl)ethoxy)methyl)-1*H*-imidazol-5-yl)pyridin-2-yl) acetamide (S15)**

900 mg (1.63 mmol) of **S14** was dissolved in 32 mL of a mixture of TFA/DCM 5% v/v. The solution was stirred overnight at ambient temperature. The reaction mixture was concentrated in vacuo and the residue was quenched with a saturated aqueous $NaHCO_3$ solution, whereupon the aqueous layer was extracted several times with EtOAc. The combined organic layers were dried over $Na_2SO_4$, filtered, and the solvents removed in vacuo. The crude product was purified via flash chromatography ($SiO_2$; *n*-hexane/EtOAc/MeOH 10:80:5) to give the pure product as a white solid in a 68% yield (500 mg, 1.11 mmol). $^1H$ NMR (400 MHz, DMSO) δ 10.61 (s, 1H), 8.33 (d, *J* = 5.0 Hz, 1H), 8.10 (s, 1H), 6.98 (dd, *J* = 5.1, 1.4 Hz, 1H), 6.87 – 6.83 (m, 1H), 6.80 (t, *J* = 7.8 Hz, 1H), 6.39 – 6.32 (m, 2H), 5.08 (s, 2H), 4.97 (s, 2H), 3.33 – 3.27 (m, 2H), 2.78 (q, *J* = 7.5 Hz, 2H), 2.08 (s, 3H), 1.31 (t, *J* = 7.5 Hz, 3H), 0.79 – 0.73 (m, 2H), -0.09 (s, 9H). $^{13}C$ NMR (101 MHz, DMSO) δ 169.2, 152.6, 150.3, 148.5, 148.2, 140.7, 137.2, 134.7, 128.4, 125.5, 121.0, 114.6, 112.7, 112.4,

71.7, 65.0, 23.9, 19.7, 17.2, 12.1, -1.5. TLC-MS (ESI+): calcd. *m/z* 451.24 for $C_{24}H_{33}N_5O_2Si$, found 473.9 $[M + Na]^+$.

***tert*-Butyl (3-(5-(2-aminopyridin-4-yl)-2-ethyl-1-((2-(trimethylsilyl)ethoxy)methyl)-1*H*-imidazol-4-yl)phenyl) carbamate (S16)**

840 mg (1.52 mmol) of **S14** was dissolved in 25 mL of MeOH and 5 mL of a 3 N NaOH solution was added. The reaction mixture was stirred at 55 °C for 6 h. After cooling down to ambient temperature, solvents were concentrated in vacuo. The oily residue was dissolved in small amounts of MeOH and a slow addition of iced water precipitated a white solid, which was collected by filtration and dried in the oven to obtain the product in 99% yield (770 mg, 1.51 mmol). $^1$H NMR (400 MHz, DMSO) δ 9.26 (s, 1H), 7.97 (d, *J* = 5.1 Hz, 1H), 7.75 (s, 1H), 7.25 (d, *J* = 7.6 Hz, 1H), 7.05 (t, *J* = 7.9 Hz, 1H), 6.83 (d, *J* = 7.7 Hz, 1H), 6.42 (d, *J* = 5.1 Hz, 1H), 6.35 (s, 1H), 6.02 (s, 2H), 5.10 (s, 2H), 3.35 – 3.29 (m, 2H), 2.78 (q, *J* = 7.4 Hz, 2H), 1.46 (s, 9H), 1.31 (t, *J* = 7.5 Hz, 3H), 0.80 – 0.74 (m, 2H), -0.07 (s, 9H). $^{13}$C NMR (101 MHz, DMSO) δ 160.2, 152.8, 150.0, 148.4, 139.5, 139.3, 135.7, 134.9, 128.1, 126.8, 120.5, 117.0, 116.4, 113.4, 109.4, 78.8, 71.7, 65.1, 28.1, 19.8, 17.2, 12.1, -1.4. TLC-MS (ESI+): calcd. *m/z* 509.28 for $C_{27}H_{39}N_5O_3Si$, found 532.1 $[M + Na]^+$.

***tert*-Butyl (3-(5-(2-((5-acrylamido-2-methoxyphenyl)amino)pyridin-4-yl)-2-ethyl-1-((2-(trimethylsilyl)ethoxy)methyl)-1*H*-imidazol-4-yl)phenyl) carbamate (S17)**

To begin, 500 mg (0.98 mmol) of **S16**, 327 mg (1.28 mmol) of *N*-(3-bromo-4-methoxyphenyl) acrylamide, and 415 mg of $Cs_2CO_3$ (1.28 mmol) in 10 mL of a mixture of 1,4-dioxane/*t*-BuOH (4 + 1) was degassed three times by evacuating and backfilling with argon under stirring. To the mixture was added 44 mg of BrettPhos Pd G3 (5 mol%), and another three cycles of evacuation and argon backfilling were carried out. The solution was stirred under reflux for 4 h. After cooling to ambient temperature, Celite was added to the mixture, and solvents were removed in vacuo. Purification via flash chromatography ($SiO_2$; *n*-hexane/EtOAc 30:70) yielded 87% (585 mg, 0.85 mmol) of a yellow solid. $^1$H NMR (400 MHz, DMSO) δ 9.96 (s, 1H), 9.23 (s, 1H), 8.42 (d, *J* = 2.5 Hz, 1H), 8.20 (d, *J* = 5.2 Hz, 1H), 8.18 (s, 1H), 7.75 (t, *J* = 1.5 Hz, 1H), 7.41 (dd, *J* = 8.8, 2.5 Hz, 1H), 7.25 (dd, *J* = 8.1, 0.8 Hz, 1H), 7.07 (t, *J* = 7.9 Hz, 1H), 6.99 (s, 1H), 6.94 (d, *J* = 8.9 Hz, 1H), 6.90 – 6.87 (m, 1H), 6.65 (dd, *J* = 5.2, 1.2 Hz, 1H), 6.45 (dd, *J* = 17.0, 10.1 Hz, 1H), 6.22 (dd, *J* = 17.0, 2.1 Hz, 1H), 5.69 (dd, *J* = 10.1, 2.0 Hz, 1H), 5.14 (s, 2H), 3.78 (s, 3H), 3.34 – 3.29 (m, 2H), 2.80 (q, *J* = 7.5 Hz, 2H), 1.43 (s, 9H), 1.32 (t, *J* = 7.5 Hz, 3H), 0.76 – 0.72 (m, 2H), -0.10 (s, 9H). $^{13}$C NMR (101 MHz, DMSO) δ 162.8, 156.3, 152.8, 150.3, 147.7, 145.4, 139.54, 139.48, 136.2, 134.8, 132.2, 131.9, 129.8, 128.2, 126.4, 126.1, 120.6, 117.1, 116.5, 116.1, 113.1, 112.5, 111.8, 110.8, 78.9, 71.8, 65.2, 55.9, 28.1, 19.8, 17.2, 12.2, -1.5. TLC-MS (ESI+): calcd. *m/z* 684.35 for $C_{37}H_{48}N_6O_5Si$, found 708.3 $[M + Na]^+$.

***N*-(3-((4-(4-(3-Aminophenyl)-2-ethyl-1-((2-(trimethylsilyl)ethoxy)methyl)-1*H*-imidazol-5-yl)pyridin-2-yl)amino)-4-methoxyphenyl) acrylamide (S18)**

570 mg (0.84 mmol) of **S17** was dissolved in 10.5 mL of a mixture of TFA/DCM 7.5% v/v. The solution was stirred for 24 h at ambient temperature. The reaction mixture was concentrated in vacuo and the residue was quenched with a saturated aqueous $NaHCO_3$ solution, whereupon the aqueous layer was extracted several times with EtOAc. The combined organic layers were dried

over $Na_2SO_4$, filtered, and the solvents removed in vacuo. The crude product was purified via flash chromatography ($SiO_2$; *n*-hexane/EtOAc/MeOH 10:80:10) to give the product as a yellow solid in a 61% yield (300 mg, 0.51 mmol). $^1$H NMR (400 MHz, DMSO) δ 9.97 (s, 1H), 8.41 (d, *J* = 2.5 Hz, 1H), 8.21 – 8.17 (m, 2H), 7.43 (dd, *J* = 8.8, 2.5 Hz, 1H), 7.00 (s, 1H), 6.95 (d, *J* = 8.9 Hz, 1H), 6.92 – 6.90 (m, 1H), 6.83 (t, *J* = 7.8 Hz, 1H), 6.66 (dd, *J* = 5.2, 1.2 Hz, 1H), 6.49 – 6.42 (m, 2H), 6.37 (ddd, *J* = 8.0, 2.2, 0.8 Hz, 1H), 6.22 (dd, *J* = 17.0, 2.1 Hz, 1H), 5.70 (dd, *J* = 10.1, 2.1 Hz, 1H), 5.12 (s, 2H), 4.96 (s, 2H), 3.79 (s, 3H), 3.31 – 3.29 (m, 2H), 2.78 (q, *J* = 7.5 Hz, 2H), 1.31 (t, *J* = 7.5 Hz, 3H), 0.77 – 0.72 (m, 2H), -0.09 (s, 9H). $^{13}$C NMR (101 MHz, DMSO) δ 162.7, 156.2, 149.9, 148.4, 147.4, 145.4, 139.9, 136.7, 134.9, 132.2, 131.9, 129.8, 128.3, 125.9, 125.8, 116.2, 114.7, 112.9, 112.7, 112.5, 112.3, 111.8, 110.8, 71.7, 65.1, 55.8, 19.7, 17.2, 12.1, -1.5. TLC-MS (ESI+): calcd. *m/z* 584.29 for $C_{32}H_{40}N_6O_3Si$, found 607.8 $[M + Na]^+$.

**Preparation of dibenzodiazepinone-carboxylic acid precursor**

**Methyl 2-amino-3-bromobenzoate (S19)**

1.08 g (5.00 mmol) of 2-amino-3-bromo-benzoic acid was dissolved in 10 mL of MeOH, 2 mL of concentrated $H_2SO_4$ was added and the reaction mixture was refluxed for 3 days. After cooling down to 0 °C the reaction mixture was quenched with a saturated $NaHCO_3$ solution under precipitation of a brown solid, which was collected by filtration. The solid was thoroughly washed with *n*-hexane and the filtrate was concentrated in vacuo to give a light-yellow solid in 56% yield (650 mg, 2.83 mmol), which was used as crude material in the next step. $^1$H NMR (400 MHz, DMSO) δ 7.78 (dd, *J* = 8.0, 1.4 Hz, 1H), 7.67 (dd, *J* = 7.7, 1.4 Hz, 1H), 6.70 (s, 2H), 6.55 (t, *J* = 7.9 Hz, 1H), 3.82 (s, 3H). $^{13}$C NMR (101 MHz, DMSO) δ 167.3, 147.6, 137.5, 130.6, 116.1, 110.9, 109.7, 51.9. TLC-MS (ESI-): calcd. *m/z* 228.97 for $C_8H_8BrNO_2$, found 213.7/215.6 $[M - CH_3]^-$.

**Methyl 2-amino-3-((2-(methoxycarbonyl)phenyl)amino)benzoate (S20)**

To begin, 370 mg (1.61 mmol) of **S19**, 218 µL (1.69 mmol) of methyl anthranilate, and 1048 mg of $Cs_2CO_3$ (3.22 mmol) in 10 mL of 1,4-dioxane were degassed three times by evacuating and backfilling with argon under stirring. To the mixture was added 36 mg of BrettPhos Pd G3 (2.5 mol%), and another three cycles of evacuation and argon backfilling were carried out. The solution was stirred at 100 °C overnight. After cooling to ambient temperature, demineralized water was added, and the aqueous phase was extracted several times with EtOAc. The combined organic layers were dried over $Na_2SO_4$, filtered, and the solvents removed in vacuo. The crude product was obtained in 73% yield (350 mg, 1.17 mmol) and used without further purification in the next step. $^1$H NMR (400 MHz, DMSO) δ 8.78 (s, 1H), 7.88 (dd, *J* = 8.0, 1.4 Hz, 1H), 7.71 (dd, *J* = 8.1, 1.1 Hz, 1H), 7.34 – 7.29 (m, 2H), 6.75 – 6.70 (m, 1H), 6.65 – 6.60 (m, 1H), 6.51 (d, *J* = 8.4 Hz, 1H), 6.47 (s, 2H), 3.86 (s, 3H), 3.82 (s, 3H). $^{13}$C NMR (101 MHz, DMSO) δ 168.2, 167.9, 148.8, 147.6, 134.5, 131.8, 131.1, 128.6, 126.5, 116.6, 114.9, 113.5, 111.2, 110.4, 51.8, 51.7. TLC-MS (ESI+): calcd. *m/z* 300.11 for $C_{16}H_{16}N_2O_4$, found 323.1 $[M + Na]^+$.

**Methyl 11-oxo-10,11-dihydro-5*H*-dibenzo[b,e][1,4]diazepine-9-carboxylate (S21)**

To 300 mg (1.00 mmol) of **S20** was added 10 mL of glacial acetic acid and the reaction mixture was refluxed for 72 h. After cooling down to 0 °C, the reaction mixture was neutralized by careful addition of an aqueous 3 M NaOH solution under precipitation of a green solid, which was collected

by filtration, washed with iced water and dried in the oven. The product was obtained as green solid in 63% yield (170 mg, 0.63 mmol). 1H NMR (400 MHz, DMSO) δ 10.24 – 9.89 (m, 1H), 8.09 (s, 1H), 7.69 (d, J = 5.2 Hz, 1H), 7.53 (d, J = 5.6 Hz, 1H), 7.39 (s, 1H), 7.29 (d, J = 5.4 Hz, 1H), 7.17 – 7.01 (m, 2H), 6.94 (s, 1H), 3.86 (s, 3H). $^{13}$C NMR (101 MHz, DMSO) δ 167.6, 167.3, 150.2, 141.1, 133.8, 132.0, 131.4, 125.1, 124.6, 124.2, 122.3, 121.3, 119.33, 119.25, 52.6. TLC-MS (ESI+): calcd. *m/z* 268.08 for $C_{15}H_{12}N_2O_3$, found 291.1 $[M + Na]^+$.

**11-Oxo-10,11-dihydro-5*H*-dibenzo[b,e][1,4]diazepine-9-carboxylic acid (S22)**

150 mg (0.56 mmol) of **S21** was dissolved in 6 mL of MeOH and 0.5 mL of an aqueous 2 N NaOH solution was added. The reaction mixture was stirred overnight at ambient temperature until complete conversion. The mixture was concentrated in vacuo and an aqueous 0.1 HCl solution was added under precipitation of a green solid. The solid was collected by filtration, washed with cold 0.1 N HCl and dried in the oven. The product was obtained as green solid in 95% yield (135 mg, 0.53 mmol). $^{1}$H NMR (400 MHz, DMSO) δ 13.66 (s, 1H), 10.55 (s, 1H), 8.05 (s, 1H), 7.70 (dd, *J* = 7.8, 1.1 Hz, 1H), 7.58 (dd, *J* = 7.8, 1.0 Hz, 1H), 7.42 – 7.36 (m, 1H), 7.27 (d, *J* = 7.1 Hz, 1H), 7.09 – 7.02 (m, 2H), 6.93 (t, *J* = 7.4 Hz, 1H). $^{13}$C NMR (101 MHz, DMSO) δ 169.2, 167.6, 150.1, 140.2, 133.9, 132.1, 132.1, 125.5, 124.6, 123.9, 122.1, 121.1, 119.2, 119.1. TLC-MS (ESI-): calcd. *m/z* 254.07 for $C_{14}H_{10}N_2O_3$, found 208.7 $[M – CO_2H]^-$.

***N*-(4-(4-(3-aminophenyl)-2-(methylthio)-1-((2-(trimethylsilyl)ethoxy)methyl)-1*H*-imidazol-5-yl)pyridin-2-yl)acetamide (S23)**

Preparation of **S23** as previously described.[4]

**Preparation of 2, 3, 4, 6 and *C*-linked fragments 10-12.**

***N*-(3-(5-(2-Acetamidopyridin-4-yl)-2-(methylthio)-1*H*-imidazol-4-yl)phenyl)-11-oxo-10,11-dihydro-5*H*-dibenzo[b,e][1,4]diazepine-9-carboxamide (2)**

To begin, 50 mg (0.11 mmol) of **S23**, 37 mg (0.14 mmol) of **S22**, and 61 mg (0.16mmol) of HATU were dissolved in 2 mL of DMF, and to the mixture was added 45 µL (0.32 mmol) of triethylamine. The reaction mixture was stirred overnight at ambient temperature. Brine was added to the reaction mixture, and the aqueous layer was extracted several times with EtOAc. The combined organic layers were dried over $Na_2SO_4$, and solvents were removed in vacuo. The residue was purified via flash chromatography ($SiO_2$; *n*-hexane/EtOAc/MeOH 20:75:5) (identification of the intermediate via TLC-MS (ESI+): calcd. *m/z* 705.26 for $C_{37}H_{39}N_7O_4SSi$, found 728.3 $[M + Na]^+$) and then directly dissolved in a 33% TFA/DCM mixture. After stirring overnight at ambient temperature, solvents were evaporated and to the residue was added a saturated aqueous $NaHCO_3$ solution. The aqueous layer was extracted several times with EtOAc. The combined organic layers were dried over $Na_2SO_4$, and solvents were removed in vacuo. The residue was purified via flash chromatography ($SiO_2$; EtOAc/ (10% IPA in EtOAc) 20:80) to obtain a 65% yield (40 mg, 0.07 mmol) of a white solid. As mixture of tautomers: $^{1}$H NMR (400 MHz, DMSO) δ 12.83 – 12.69 (m, 1H), 10.63 – 10.51 (m, 1H), 10.49 – 10.31 (m, 1H), 10.12 – 9.98 (m, 1H), 8.38 – 8.11 (m, 2H), 8.10 – 8.01 (m, 1H), 7.97 – 7.86 (m, 1H), 7.83 – 7.70 (m, 1H), 7.69 – 7.63 (m, 1H), 7.48 – 7.40 (m, 1H), 7.39 – 7.27 (m, 2H), 7.26 – 7.21 (m, 1H), 7.21 – 7.17 (m, 1H), 7.16 – 7.11 (m, 1H), 7.11

– 7.02 (m, 2H), 6.94 (t, *J* = 7.5 Hz, 1H), 2.63 (s, 3H), 2.11 – 2.01 (m, 3H). HRMS (ESI): exact mass calcd. for $C_{31}H_{25}N_7O_3S$ $[M + Na]^+$: 598.16318, found: 598.16425.

**_N_-(3-(5-(2-Acetamidopyridin-4-yl)-2-ethyl-1_H_-imidazol-4-yl)phenyl)-11-oxo-10,11-dihydro-5_H_-dibenzo[b,e][1,4]diazepine-9-carboxamide (3)**

To begin, 50 mg (0.11 mmol) of **S15**, 31 mg (0.12 mmol) of **S22**, and 55 mg (0.14 mmol) of HATU were dissolved in 2 mL of DMF, and to the mixture was added 31 µL (0.22 mmol) of triethylamine. The reaction mixture was stirred overnight at ambient temperature. Demineralized water was added to the reaction mixture under precipitation of a yellowish solid, which was collected by filtration and dried in the oven. (Identification of the intermediate via TLC-MS (ESI+): calcd. *m*/*z* 687.30 for $C_{38}H_{41}N_7O_4Si$, found 710.8 $[M + Na]^+$) The solid was then directly dissolved in 2.5 mL of DCM and the mixture was cooled down to 0°C. After addition of 100 µL of MSA, the mixture was slowly warmed up to ambient temperature and stirred for 1 h. To the mixture was added a saturated aqueous $NaHCO_3$ solution, and the aqueous layer was extracted several times with EtOAc. Combined organic layers were dried over $Na_2SO_4$, and solvents were removed in vacuo. The residue was purified via flash chromatography ($SiO_2$; DCM/MeOH 90:10) to obtain a 50% yield (31 mg, 0.06 mmol) of a white solid. As mixture of tautomers: $^1H$ NMR (400 MHz, DMSO) δ 12.41 – 12.19 (m, 1H), 10.63 – 10.48 (m, 1H), 10.48 – 10.26 (m, 1H), 10.16 – 9.99 (m, 1H), 8.44 – 8.05 (m, 3H), 8.00 – 7.86 (m, 1H), 7.82 – 7.62 (m, 2H), 7.49 – 7.28 (m, 3H), 7.24 (d, *J* = 7.8 Hz, 1H), 7.19 (d, *J* = 7.7 Hz, 1H), 7.16 – 7.00 (m, 3H), 6.94 (t, *J* = 7.5 Hz, 1H), 2.77 – 2.64 (m, 2H), 2.04 (s, 3H), 1.28 (t, *J* = 7.6 Hz, 3H). HRMS (ESI): exact mass calcd. for $C_{32}H_{27}N_7O_3$ $[M + Na]^+$: 580.20676, found: 580.20779.

**_N_-(3-(5-(2-((5-Acrylamido-2-methoxyphenyl)amino)pyridin-4-yl)-2-ethyl-1_H_-imidazol-4-yl)phenyl)-11-oxo-10,11-dihydro-5_H_-dibenzo[b,e][1,4]diazepine-9-carboxamide (4)**

To begin, 50 mg (0.09 mmol) of **S18**, 26 mg (0.10 mmol) of **S22**, and 49 mg (0.13 mmol) of HATU were dissolved in 2 mL of DMF, and to the mixture was added 36 µL (0.26 mmol) of triethylamine. The reaction mixture was stirred overnight at ambient temperature. Demineralized water was added to the reaction mixture under precipitation of a yellowish solid, which was collected by filtration and dried in the oven. (Identification of the intermediate via TLC-MS (ESI+): calcd. *m*/*z* 820.35 for $C_{46}H_{48}N_8O_5Si$, found 844.0 $[M + Na]^+$) The solid was then directly dissolved in 2 mL of DCM and the mixture was cooled down to 0°C. After addition of 100 µL of MSA, the mixture was slowly warmed up to ambient temperature and stirred for 1 h. To the mixture was added a saturated aqueous $NaHCO_3$ solution, and the aqueous layer was extracted several times with EtOAc. Combined organic layers were dried over $Na_2SO_4$, and solvents were removed in vacuo. The residue was purified via flash chromatography ($SiO_2$; *n*-hexane/EtOAc 15:85 to EtOAc/IPA 85:15) to obtain a 30% yield (18 mg, 0.03 mmol) of a white solid. As mixture of tautomers: $^1H$ NMR (400 MHz, DMSO) δ 12.25 – 12.16 (m, 1H), 10.65 – 10.53 (m, 1H), 10.11 – 10.03 (m, 1H), 9.98 – 9.90 (m, 1H), 8.36 – 8.29 (m, 1H), 8.10 – 8.06 (m, 1H), 8.04 – 7.99 (m, 1H), 7.98 – 7.93 (m, 1H), 7.88 – 7.69 (m, 2H), 7.67 (d, *J* = 7.8 Hz, 1H), 7.46 – 7.42 (m, 1H), 7.42 – 7.40 (m, 1H), 7.39 – 7.35 (m, 1H), 7.34 – 7.32 (m, 1H), 7.30 – 7.20 (m, 2H), 7.19 – 7.09 (m, 2H), 7.05 (d, *J* = 8.0 Hz, 1H), 6.96 – 6.89 (m, 2H), 6.73 (dd, *J* = 5.3, 0.8 Hz, 1H), 6.48 – 6.40 (m, 1H), 6.24 – 6.16 (m, 1H), 5.71 – 5.65 (m, 1H), 3.82 – 3.75 (m, 3H), 2.74 – 2.67 (m, 2H), 1.29 (t, *J* = 7.6 Hz, 3H). HRMS (ESI): exact mass calcd. for $C_{40}H_{34}N_8O_4$ $[M + Na]^+$: 713.25952, found: 713.25995.

***N*-(4-(5-(3-aminophenyl)-2-(methylthio)-1*H*-imidazol-4-yl)pyridin-2-yl)acetamide (6)**

To begin, 40 mg (0.09 mmol) **S23** were suspended in 2 ml of a 4 N HCl in dioxane and stirred overnight at ambient temperature. To the mixture was added a saturated aqueous $NaHCO_3$ solution, and the aqueous layer was extracted several times with EtOAc. Combined organic layers were dried over $Na_2SO_4$, and solvents were removed in vacuo. The residue was purified via flash chromatography ($SiO_2$; DCM/MeOH 90:10) to obtain an off-white solid in 69% yield (20 mg, 0.06 mmol). As mixture of tautomers: $^1$H NMR (400 MHz, DMSO) δ 12.81 – 12.39 (m, 1H), 10.59 – 10.14 (m, 1H), 8.50 – 8.00 (m, 2H), 7.15 – 6.90 (m, 2H), 6.78 – 6.45 (m, 3H), 5.40 – 4.94 (m, 2H), 2.59 (s, 3H), 2.15 – 2.01 (m, 3H). $^{13}$C NMR (101 MHz, DMSO) δ 168.8, 152.4, 148.8, 147.2, 144.0, 141.4, 133.7, 132.1, 130.7, 129.2, 116.3, 116.0, 114.0, 113.7, 110.6, 23.8, 15.1. TLC-MS (ESI+): calcd. *m*/*z* 339.12 for $C_{17}H_{17}N_5OS$, found 361.9 $[M + Na]^+$.

**11-oxo-*N*-phenyl-10,11-dihydro-5*H*-dibenzo[b,e][1,4]diazepine-9-carboxamide (10)**

To begin, 50 mg (0.19 mmol) of **S22**, 23 mg (0.24 mmol) of aniline, and 106 mg (0.28 mmol) of HATU were dissolved in 2 mL of DMF, and to the mixture was added 97 µL (0.56 mmol) of *N*,*N*-diisopropylethylamin. The reaction mixture was stirred overnight at ambient temperature. Demineralized water was added to the reaction mixture under precipitation of a white solid, which was collected by filtration, washed with water and dried in the oven. The crude product was purified via flash chromatography ($SiO_2$; DCM/MeOH 90:10) to obtain a white solid in 50% (31 mg, 0.09 mmol) yield. $^1$H NMR (400 MHz, DMSO) δ 10.51 (s, 1H), 10.09 (s, 1H), 8.07 (s, 1H), 7.74 (d, *J* = 7.8 Hz, 2H), 7.68 (dd, *J* = 7.9, 1.5 Hz, 1H), 7.41 – 7.34 (m, 4H), 7.24 (dd, *J* = 7.9, 1.1 Hz, 1H), 7.16 – 7.11 (m, 2H), 7.06 (dd, *J* = 8.1, 0.6 Hz, 1H), 6.96 – 6.92 (m, 1H). TLC-MS (ESI-): calcd. *m*/*z* 329.12 for $C_{20}H_{15}N_3O_2$, found 328.4 $[M - H]^-$.

**11-oxo-*N*-(thiazol-2-yl)-10,11-dihydro-5*H*-dibenzo[b,e][1,4]diazepine-9-carboxamide (11)**

To begin, 50 mg (0.19 mmol) of **S22**, 24 mg (0.24 mmol) of 2-aminothiazole, and 106 mg (0.28 mmol) of HATU were dissolved in 2 mL of DMF, and to the mixture was added 97 µL (0.56 mmol) of *N*,*N*-diisopropylethylamin. The reaction mixture was stirred overnight at ambient temperature. Demineralized water was added to the reaction mixture under precipitation of a white solid, which was collected by filtration, washed with water and dried in the oven. The crude product was purified via flash chromatography ($SiO_2$; DCM/MeOH 90:10 and *n*-hexane/(EtOAc/IPA 10%) 30:70) to obtain a white solid in 51% (32 mg, 0.10 mmol) yield. $^1$H NMR (400 MHz, DMSO) δ 12.80 (s, 1H), 10.16 (s, 1H), 8.06 (s, 1H), 7.68 (dd, *J* = 7.8, 1.2 Hz, 1H), 7.57 (d, *J* = 3.6 Hz, 1H), 7.44 (d, *J* = 7.6 Hz, 1H), 7.41 – 7.36 (m, 1H), 7.31 (d, *J* = 3.6 Hz, 1H), 7.27 – 7.23 (m, 1H), 7.11 (t, *J* = 7.8 Hz, 1H), 7.05 (d, *J* = 8.0 Hz, 1H), 6.95 (t, *J* = 7.5 Hz, 1H). TLC-MS (ESI-): calcd. *m*/*z* 336.07 for $C_{17}H_{12}N_4O_2S$, found 335.3 $[M - H]^-$.

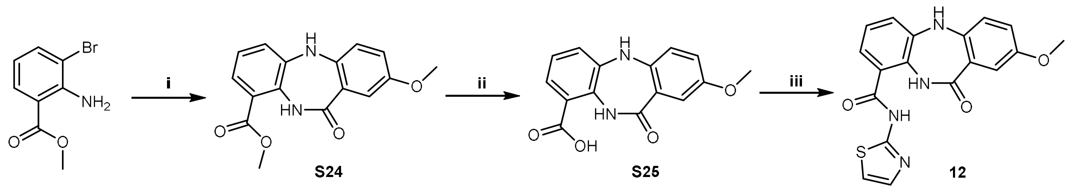

**Scheme S3:** Preparation of **12** – Reagents and conditions are as follows: i) a) Methyl 2-amino-5-methoxybenzoate, BrettPhos Pd G3, $Cs_2CO_3$, 1,4-dioxane, rf; b) glacial acetic acid, rf, 68%; ii) 5 M NaOH, MeOH, rt, 94%; iii) 2-aminothiazole, TBTU, $Et_3N$, $CH_2Cl_2$, rt, 52%.

**Methyl 2-methoxy-11-oxo-10,11-dihydro-5*H*-dibenzo[b,e][1,4]diazepine-9-carboxylate (S24)**

To begin, 400 mg (1.74 mmol) of methyl 2-amino-3-bromobenzoate, 308 mg (1.70 mmol) methyl 2-amino-5-methoxybenzoate, and 763 mg (2.34 mmol) $Cs_2CO_3$ in 25 mL of dry 1,4-dioxane were degassed three times by evacuation and backfilling with nitrogen gas. 45 mg (0.050 mmol) of BrettPhos Pd G3 was added, and another three cycles of evacuation and nitrogen backfilling were performed. With strong stirring, the solution was heated to 100 ºC for 24h under nitrogen. The solution was then filtered through celite, the filtrate concentrated to dryness in vacuo (Identification of the intermediate via ITMS (ESI+): calcd. *m/z* 330.12 for $C_{17}H_{18}N_2O_5$, found 331.17 $[M + H]^+$), and the resulting solid redissolved in glacial acetic acid and allowed to reflux for 48h. The solution was then poured into a beaker of ice water and neutralized by the addition of a saturated $NaHCO_3$ solution, resulting in the precipitation of a solid which was collected by filtration, washed with 0.1M HCl, and dried in the oven to give 346 mg (68% yield) of the product as a green solid. $^1H$ NMR (400 MHz, DMSO) δ 10.06 (s, 1H), 7.80 (s, 1H), 7.53 (dd, $J$ = 7.8, 1.5 Hz, 1H), 7.29 (dd, $J$ = 7.9, 1.5 Hz, 1H), 7.20 (d, $J$ = 2.8 Hz, 1H), 7.10 (t, $J$ = 7.9 Hz, 1H), 7.05 (dd, $J$ = 8.8, 2.8 Hz, 1H), 7.01 (d, $J$ = 8.7 Hz, 1H), 3.87 (s, 3H), 3.72 (s, 3H). $^{13}C$ NMR (126 MHz, DMSO) δ 167.9, 167.7, 154.4, 144.2, 142.7, 131.8, 125.3, 124.8, 124.8, 123.9, 121.4, 121.2, 120.0, 115.1, 55.8, 53.0. ITMS (ESI+): calcd. *m/z* 298.10 for $C_{16}H_{14}N_2O_4$, found 299.17 $[M+H]^+$

**2-Methoxy-11-oxo-10,11-dihydro-5*H*-dibenzo[b,e][1,4]diazepine-9-carboxylic acid (S25)**

To begin, 331 mg (1.11 mmol) of **S24** was dissolved in 30 mL MeOH with 5 mL of a 5 M NaOH solution. With strong stirring, the solution was allowed to stir overnight at room temperature. After TLC indicated complete conversion, the solvent volume was reduced in vacuo and acidified by the dropwise addition of a 1 M HCl solution resulting in the precipitation of a green solid. The solid was collected by filtration, washed with a small amount of 0.1 M HCl, and dried in the oven to give 298 mg (94% yield) of the product as a green solid. $^1H$ NMR (400 MHz, DMSO) δ 13.58 (s, 1H), 10.51 (s, 1H), 7.75 (s, 1H), 7.57 (dd, $J$ = 7.8, 1.5 Hz, 1H), 7.26 (dd, $J$ = 7.9, 1.5 Hz, 1H), 7.21 (d, $J$ = 2.8 Hz, 1H), 7.09 – 6.99 (m, 3H), 3.72 (s, 3H). $^{13}C$ NMR (126 MHz, DMSO) δ 169.6, 167.9, 154.3, 144.2, 141.8, 132.6, 125.7, 124.8, 124.5, 123.7, 121.5, 121.1, 119.8, 115.2, 55.8. ITMS (ESI+): calcd. *m/z* 284.08 for $C_{15}H_{12}N_2O_4$, found 285.17 $[M+H]^+$

**2-Methoxy-11-oxo-*N*-(thiazol-2-yl)-10,11-dihydro-5*H*-dibenzo[b,e][1,4]diazepine-9-carboxamide (12)**

To begin, 151 mg (0.532 mmol) of **2**, 63 mg (0.63 mmol) of 2-aminothiazole, and 225 mg (0.701 mmol) of TBTU were added to approximately 15 mL of DCM. With strong stirring, 0.5 mL of

$Et_3N$ was added dropwise and the solution was allowed to stir overnight at room temperature. The reaction mixture was then diluted with DCM, added to a separatory funnel, and washed with brine. The organic layer was collected, dried over $Na_2SO_4$, filtered, and concentrated in vacuo to give a red oil, which was then purified via column chromatography ($SiO_2$; 0-50% EtOAc in hexanes) to obtain 100 mg (52% yield) of the product as a pale yellow solid. $^1$H NMR (500 MHz, DMSO) δ 12.72 (s, 1H), 10.01 (s, 1H), 7.79 (s, 1H), 7.57 (d, $J$ = 3.6 Hz, 1H), 7.39 (s, 1H), 7.32 (s, 1H), 7.24 (d, $J$ = 7.8 Hz, 1H), 7.18 (d, $J$ = 2.7 Hz, 1H), 7.11 (t, $J$ = 7.8 Hz, 1H), 7.05 – 6.99 (m, 2H), 3.71 (s, 3H). ITMS (ESI+): calcd. *m/z* 366.08 for $C_{18}H_{14}N_4O_3S$, found 367.17 $[M+H]^+$

# HPLC Traces

## Compound **1**

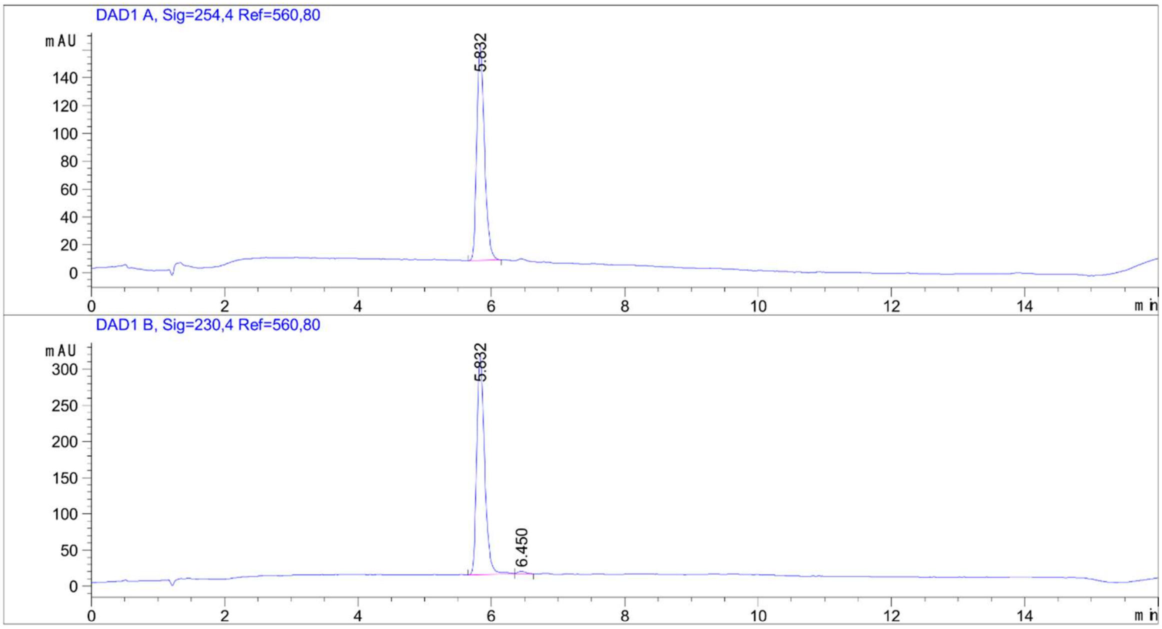


=====================================================================

Area Percent Report

=====================================================================

Sorted By : Signal
Multiplier: : 1.0000
Dilution: : 1.0000
Use Multiplier & Dilution Factor with ISTDs

Signal 1: DAD1 A, Sig=254,4 Ref=560,80

| Peak # | RetTime [min] | Type | Width [min] | Area [mAU*s] | Height [mAU] | Area % |
|---|---|---|---|---|---|---|
| 1 | 5.832 | BB | 0.1233 | 1242.57690 | 155.29688 | 100.0000 |

Totals : 1242.57690 155.29688

Signal 2: DAD1 B, Sig=230,4 Ref=560,80

| Peak # | RetTime [min] | Type | Width [min] | Area [mAU*s] | Height [mAU] | Area % |
|---|---|---|---|---|---|---|
| 1 | 5.832 | BV | 0.1256 | 2499.54956 | 304.93323 | 98.6797 |
| 2 | 6.450 | VB | 0.1416 | 33.44312 | 3.76729 | 1.3203 |

Totals : 2532.99268 308.70051

=====================================================================

*** End of Report ***

Compound **2**

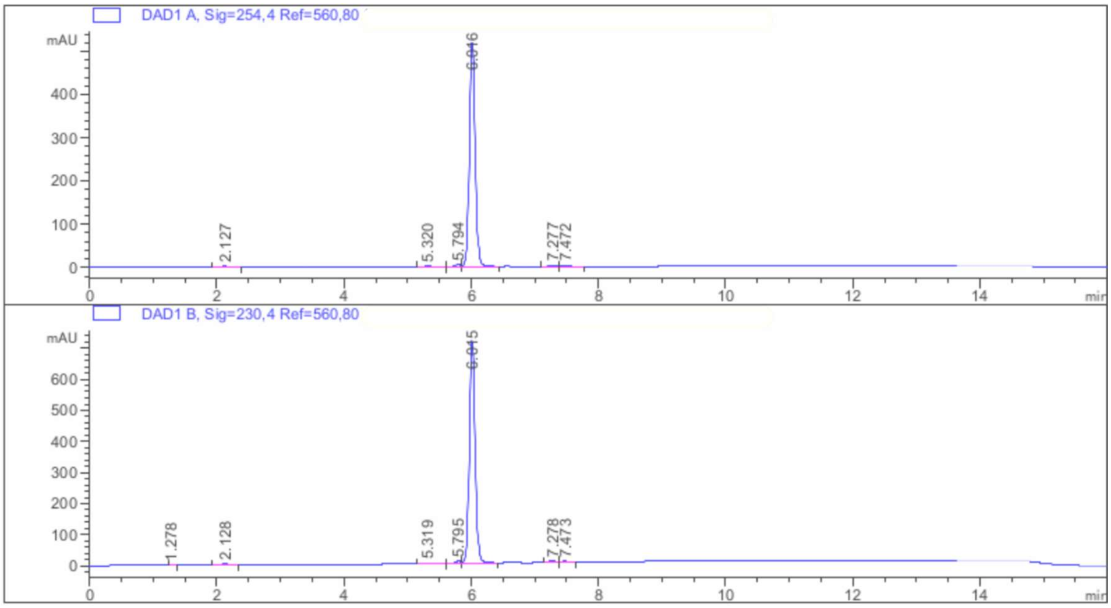


Area Percent Report

Sorted By : Signal
Multiplier: : 1.0000
Dilution: : 1.0000
Use Multiplier & Dilution Factor with ISTDs

Signal 1: DAD1 A, Sig=254,4 Ref=560,80

| Peak # | RetTime [min] | Type | Width [min] | Area [mAU*s] | Height [mAU] | Area % |
|---|---|---|---|---|---|---|
| 1 | 2.127 | BB | 0.1125 | 23.93348 | 3.22657 | 0.7072 |
| 2 | 5.320 | BB | 0.1192 | 21.80744 | 2.67255 | 0.6444 |
| 3 | 5.794 | BV | 0.1026 | 34.24997 | 5.08063 | 1.0121 |
| 4 | 6.016 | VB | 0.0974 | 3262.41772 | 518.84698 | 96.4036 |
| 5 | 7.277 | BV | 0.1024 | 24.70338 | 3.77010 | 0.7300 |
| 6 | 7.472 | VB | 0.1131 | 17.01361 | 2.22797 | 0.5027 |

Totals : 3384.12561 535.82480

Signal 2: DAD1 B, Sig=230,4 Ref=560,80

| Peak # | RetTime [min] | Type | Width [min] | Area [mAU*s] | Height [mAU] | Area % |
|---|---|---|---|---|---|---|
| 1 | 1.278 | BB | 0.0473 | 6.12392 | 2.02462 | 0.1318 |
| 2 | 2.128 | BB | 0.1094 | 38.35267 | 5.36624 | 0.8255 |
| 3 | 5.319 | BB | 0.1173 | 32.49109 | 4.06219 | 0.6993 |
| 4 | 5.795 | BV | 0.1055 | 47.96972 | 7.03979 | 1.0325 |
| 5 | 6.015 | VB | 0.0970 | 4465.96436 | 713.48822 | 96.1216 |
| 6 | 7.278 | BV | 0.1002 | 34.24449 | 5.24423 | 0.7370 |
| 7 | 7.473 | VB | 0.1064 | 21.01408 | 2.97528 | 0.4523 |

Totals : 4646.16032 740.20056

*** End of Report ***

Compound **3**

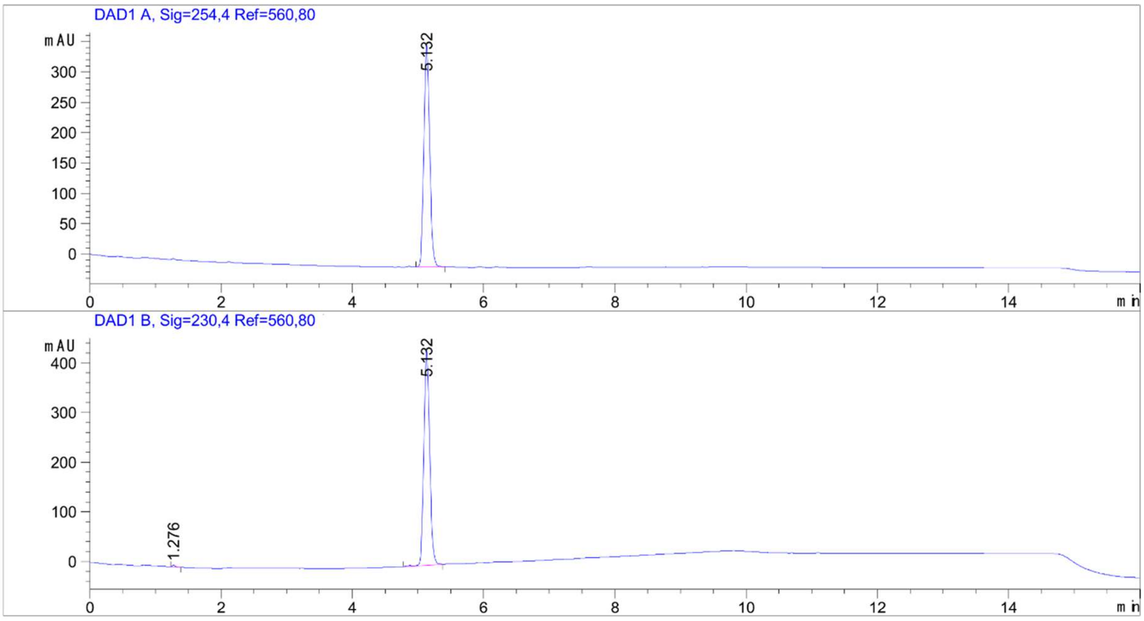


=====================================================================

Area Percent Report

=====================================================================

Sorted By : Signal
Multiplier: : 1.0000
Dilution: : 1.0000
Use Multiplier & Dilution Factor with ISTDs

Signal 1: DAD1 A, Sig=254,4 Ref=560,80

| Peak # | RetTime [min] | Type | Width [min] | Area [mAU*s] | Height [mAU] | Area % |
|---|---|---|---|---|---|---|
| 1 | 5.132 | BB | 0.0954 | 2252.04419 | 368.15723 | 100.0000 |

Totals : 2252.04419 368.15723

Signal 2: DAD1 B, Sig=230,4 Ref=560,80

| Peak # | RetTime [min] | Type | Width [min] | Area [mAU*s] | Height [mAU] | Area % |
|---|---|---|---|---|---|---|
| 1 | 1.276 | BB | 0.0470 | 10.50854 | 3.50590 | 0.3914 |
| 2 | 5.132 | BB | 0.0957 | 2674.60620 | 435.11304 | 99.6086 |

Totals : 2685.11474 438.61894

=====================================================================

*** End of Report ***

Compound **4**

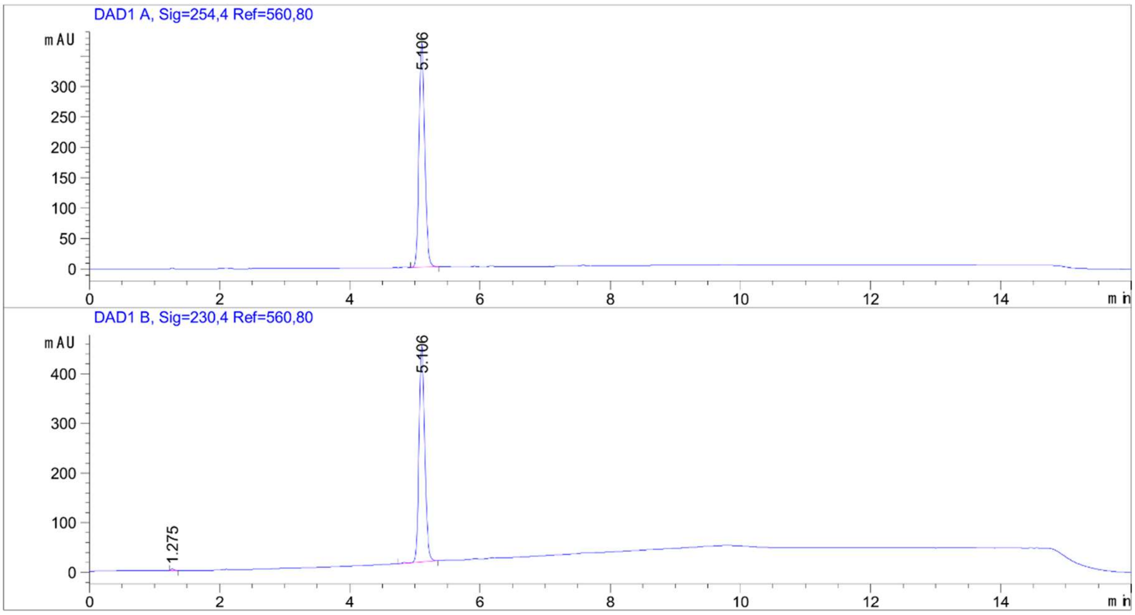


=====================================================================

Area Percent Report

=====================================================================

Sorted By : Signal
Multiplier: : 1.0000
Dilution: : 1.0000
Use Multiplier & Dilution Factor with ISTDs

Signal 1: DAD1 A, Sig=254,4 Ref=560,80

| Peak # | RetTime [min] | Type | Width [min] | Area [mAU*s] | Height [mAU] | Area % |
|---|---|---|---|---|---|---|
| 1 | 5.106 | BB | 0.0958 | 2269.39600 | 368.60446 | 100.0000 |

Totals : 2269.39600 368.60446

Signal 2: DAD1 B, Sig=230,4 Ref=560,80

| Peak # | RetTime [min] | Type | Width [min] | Area [mAU*s] | Height [mAU] | Area % |
|---|---|---|---|---|---|---|
| 1 | 1.275 | BB | 0.0476 | 10.04510 | 3.29362 | 0.3724 |
| 2 | 5.106 | BB | 0.0960 | 2687.35864 | 435.36902 | 99.6276 |

Totals : 2697.40375 438.66263

=====================================================================

*** End of Report ***

Compound **6**

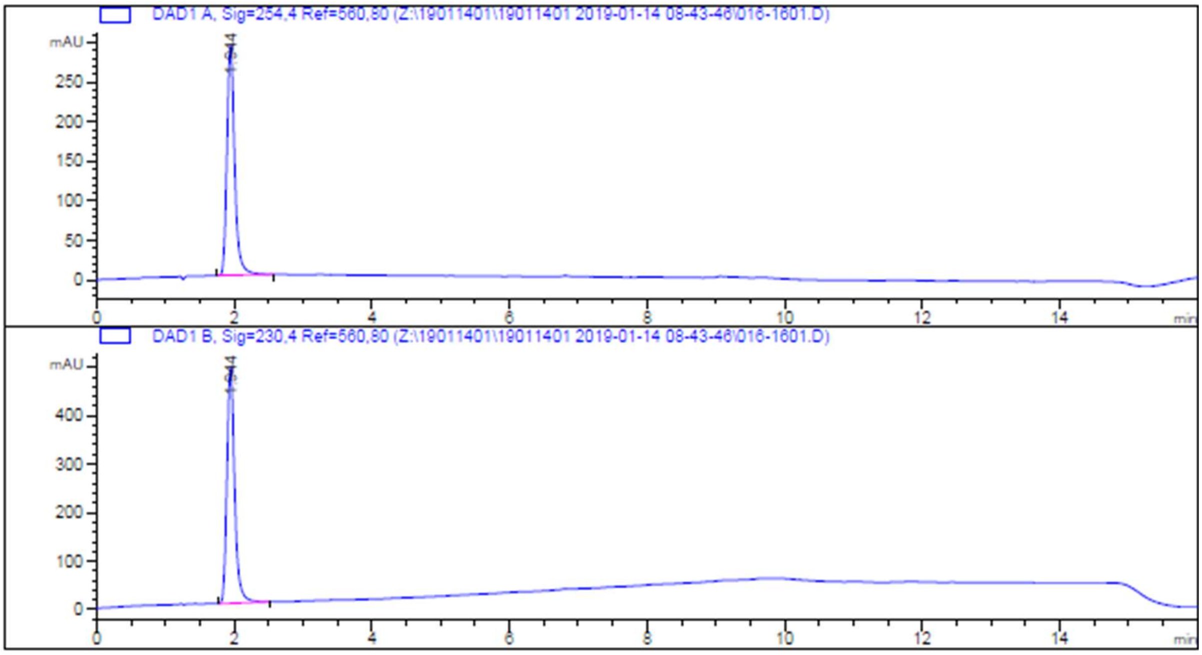


=====================================================================

Area Percent Report

=====================================================================

Sorted By : Signal
Multiplier: : 1.0000
Dilution: : 1.0000
Use Multiplier & Dilution Factor with ISTDs

Signal 1: DAD1 A, Sig=254,4 Ref=560,80

| Peak # | RetTime [min] | Type | Width [min] | Area [mAU*s] | Height [mAU] | Area % |
|---|---|---|---|---|---|---|
| 1 | 1.944 | BB | 0.1188 | 2271.73779 | 291.97125 | 100.0000 |

Totals : 2271.73779 291.97125

Signal 2: DAD1 B, Sig=230,4 Ref=560,80

| Peak # | RetTime [min] | Type | Width [min] | Area [mAU*s] | Height [mAU] | Area % |
|---|---|---|---|---|---|---|
| 1 | 1.944 | BB | 0.1183 | 3787.05591 | 489.24664 | 100.0000 |

Totals : 3787.05591 489.24664

Compound **10**

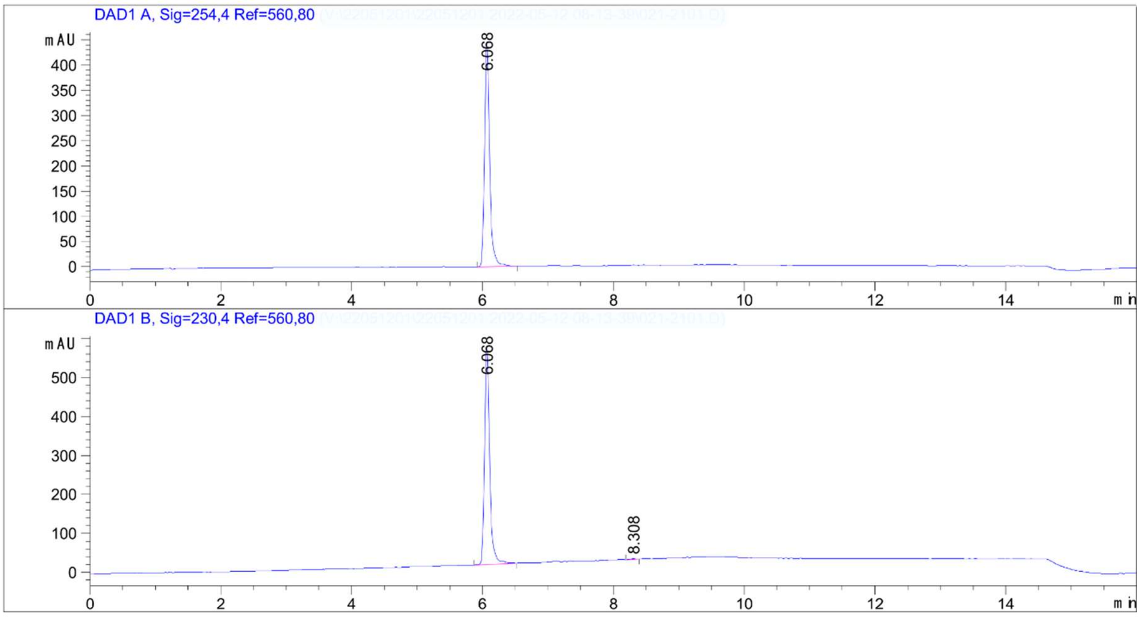


=====================================================================

Area Percent Report

=====================================================================

Sorted By : Signal
Multiplier: : 1.0000
Dilution: : 1.0000
Use Multiplier & Dilution Factor with ISTDs

Signal 1: DAD1 A, Sig=254,4 Ref=560,80

| Peak # | RetTime [min] | Type | Width [min] | Area [mAU*s] | Height [mAU] | Area % |
|---|---|---|---|---|---|---|
| 1 | 6.068 | BB | 0.0814 | 2409.17285 | 443.48016 | 100.0000 |
| Totals : | | | | 2409.17285 | 443.48016 | |

Signal 2: DAD1 B, Sig=230,4 Ref=560,80

| Peak # | RetTime [min] | Type | Width [min] | Area [mAU*s] | Height [mAU] | Area % |
|---|---|---|---|---|---|---|
| 1 | 6.068 | BB | 0.0817 | 3046.58838 | 558.33026 | 99.6039 |
| 2 | 8.308 | BB | 0.0721 | 12.11452 | 2.43312 | 0.3961 |
| Totals : | | | | 3058.70290 | 560.76338 | |

=====================================================================

*** End of Report ***

Compound **11**

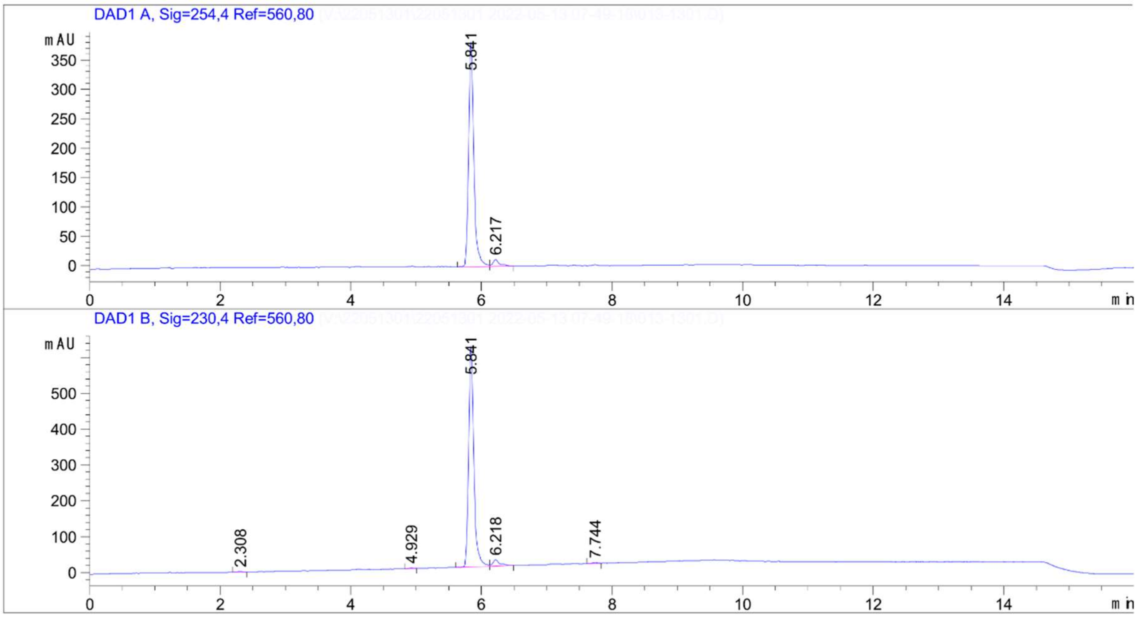


=====================================================================

Area Percent Report

=====================================================================

Sorted By : Signal
Multiplier: : 1.0000
Dilution: : 1.0000
Use Multiplier & Dilution Factor with ISTDs

Signal 1: DAD1 A, Sig=254,4 Ref=560,80

| Peak # | RetTime [min] | Type | Width [min] | Area [mAU*s] | Height [mAU] | Area % |
|---|---|---|---|---|---|---|
| 1 | 5.841 | BV | 0.0827 | 2111.23096 | 380.40961 | 96.3715 |
| 2 | 6.217 | VB | 0.0994 | 79.48979 | 11.67710 | 3.6285 |

Totals : 2190.72075 392.08670

Signal 2: DAD1 B, Sig=230,4 Ref=560,80

| Peak # | RetTime [min] | Type | Width [min] | Area [mAU*s] | Height [mAU] | Area % |
|---|---|---|---|---|---|---|
| 1 | 2.308 | BB | 0.0820 | 8.00557 | 1.41610 | 0.2250 |
| 2 | 4.929 | BB | 0.0772 | 7.43863 | 1.41716 | 0.2090 |
| 3 | 5.841 | BV | 0.0827 | 3396.12769 | 612.81683 | 95.4291 |
| 4 | 6.218 | VB | 0.1038 | 132.57205 | 18.46015 | 3.7252 |
| 5 | 7.744 | BB | 0.0894 | 14.65253 | 2.53782 | 0.4117 |

Totals : 3558.79647 636.64806

=====================================================================

*** End of Report ***

Compound **12**

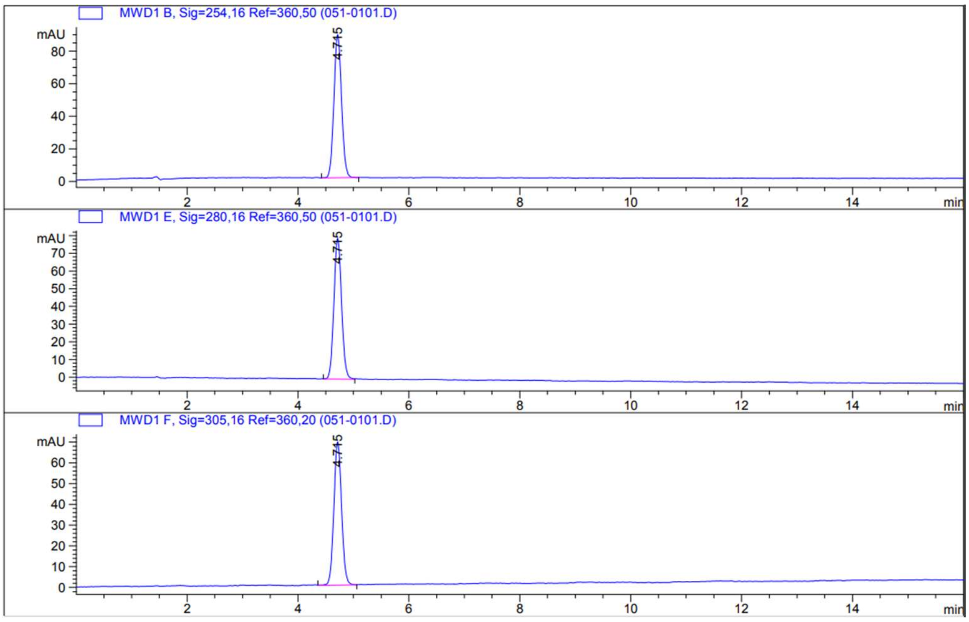


```
=====================================================================
                         Area Percent Report
=====================================================================

Sorted By             :      Signal
Multiplier:                    :      1.0000
Dilution:                      :      1.0000
Use Multiplier & Dilution Factor with ISTDs


Signal 1: MWD1 B, Sig=254,16 Ref=360,50

Peak RetTime Type  Width     Area       Height      Area
  #   [min]        [min]   [mAU*s]      [mAU]        %
----|-------|----|-------|----------|----------|--------|
   1   4.715 BB     0.1496  839.64484   87.78494 100.0000

Totals :                    839.64484   87.78494

Signal 2: MWD1 E, Sig=280,16 Ref=360,50

Peak RetTime Type  Width     Area       Height      Area
  #   [min]        [min]   [mAU*s]      [mAU]        %
----|-------|----|-------|----------|----------|--------|
   1   4.715 VV     0.1502  766.02618   79.65793 100.0000

Totals :                    766.02618   79.65793


Signal 3: MWD1 F, Sig=305,16 Ref=360,20

Peak RetTime Type  Width     Area       Height      Area
  #   [min]        [min]   [mAU*s]      [mAU]        %
----|-------|----|-------|----------|----------|--------|
   1   4.715 VV     0.1506  666.41174   69.04562 100.0000

Totals :                    666.41174   69.04562

=====================================================================
                          *** End of Report ***
```

**Supplementary References.**


1 Jia, Y. et al. Overcoming EGFR(T790M) and EGFR(C797S) resistance with mutant-selective allosteric inhibitors. Nature 534, 129-132, doi:10.1038/nature17960 (2016).

2 Ichihara, O., Barker, J., Law, R. J. & Whittaker, M. Compound Design by Fragment-Linking. Molecular Informatics 30, 298-306, doi:https://doi.org/10.1002/minf.201000174 (2011).

3 De Clercq, D. J. H. et al. Discovery and Optimization of Dibenzodiazepinones as Allosteric Mutant-Selective EGFR Inhibitors. ACS Med. Chem. Lett. 10, 1549-1553, doi:10.1021/acsmedchemlett.9b00381 (2019).

4 Wittlinger, F. et al. Design of a "Two-in-One" Mutant-Selective Epidermal Growth Factor Receptor Inhibitor That Spans the Orthosteric and Allosteric Sites. J. Med. Chem. 65, 1370-1383, doi:10.1021/acs.jmedchem.1c00848 (2022).

5 Gilligan, Paul Joseph & Bakthavatchalam, Rajagopal (2000). 1H-Imidazo[4,5-d]pyridazin-7-ones, 3H-imidazo[4,5-c]pyridin-4-ones, and corresponding thiones as corticotropin releasing factor (CRF) receptor ligands (WO 00/39127). World intellectual property organization.
