# Supplementary material for: Linking ATP and allosteric sites to achieve superadditive binding with bivalent EGFR kinase inhibitors
Source: Commun Chem. 2024 Feb 20;7:38. doi: 10.1038/s42004-024-01108-3 (PMC10879502; doi:10.1038/s42004-024-01108-3)
Supplement: Supplementary file 3 — Description of Additional Supplementary Files [file 42004_2024_1108_MOESM3_ESM.pdf]

Description of Additional Supplementary Files

File name: Supplementary Data 1

Description: Copies of the ^{1}H and ^{13}C spectra of isolated new compounds

File name: Supplementary Data 2

Description: Percent activity data for figure 4

File name: Supplementary Data 3

Description: Atomic coordinates and other crystallographic information for the cocrystal structures of **1** (PDB ID 8FV3) in complex with EGFR(T790M/V948R).

File name: Supplementary Data 4

Description: Atomic coordinates and other crystallographic information for the cocrystal structures of **2** (PDB ID 8FV4) in complex with EGFR(T790M/V948R).
