# Supplementary material for: Linking ATP and allosteric sites to achieve superadditive binding with bivalent EGFR kinase inhibitors
Source: Commun Chem. 2024 Feb 20;7:38. doi: 10.1038/s42004-024-01108-3 (PMC10879502; doi:10.1038/s42004-024-01108-3)

Supplementary Data 1

Linking ATP and allosteric sites to achieve superadditive binding with bivalent EGFR kinase inhibitors

Florian Wittlinger[1,#], Blessing C. Ogboo[2,#], Ekaterina Shevchenko[1,3,4] , Tahereh Damghani[2], Calvin D. Pham[2], Ilse K. Schaeffner[5,6], Brandon T. Oligny[2], Surbhi P. Chitnis[2], Tyler S. Beyett[5,6,12], Alexander Rasch[1], Brian Buckley[7], Daniel A. Urul[8], Tatiana Shaurova[9], Earl W. May[8], Erik M. Schaefer[8], Michael J. Eck[5,6], Pamela A. Hershberger[9], Antti Poso[1,3,4,10], Stefan A. Laufer[1,3,4]*, David E. Heppner[2,9,11]*

1. Department of Pharmaceutical and Medicinal Chemistry, Institute of Pharmaceutical Sciences, Eberhard Karls Universität Tübingen, Auf der Morgenstelle 8, 72076 Tübingen, Germany

2. Department of Chemistry, University at Buffalo, The State University of New York, Buffalo, NY, 14260, USA

3. Cluster of Excellence iFIT (EXC 2180) "Image-Guided and Functionally Instructed Tumor Therapies" Eberhard Karls Universität Tübingen, 72076 Tübingen, Germany.

4. Tübingen Center for Academic Drug Discovery & Development (TüCAD2), 72076 Tübingen, Germany

5. Department of Cancer Biology, Dana-Farber Cancer Institute, Boston, MA, 02215 USA

6. Department of Biological Chemistry and Molecular Pharmacology, Harvard Medical School, Boston, MA, 02115 USA

7. Department of Cell Stress Biology, Roswell Park Comprehensive Cancer Center, Buffalo, NY,14203, USA

8. AssayQuant Technologies, Inc., Marlboro, MA, 01752, USA

9. Department of Pharmacology and Therapeutics, Roswell Park Comprehensive Cancer Center, Buffalo, NY,14203, USA

10. School of Pharmacy, University of Eastern Finland, 70210 Kuopio, Finland

11. Department of Structural Biology, University at Buffalo, The State University of New York, Buffalo, NY, 14260, USA

12. Present Address: Department of Pharmacology and Chemical Biology, Emory University School of Medicine, 5119 Rollins Research Center, 1510 Clifton Rd, Atlanta, GA 30322, USA

*These authors jointly supervised this work:

David E. Heppner (0000-0002-0722-5160) davidhep@buffalo.edu

Stefan A. Laufer (0000-0001-6952-1486) stefan.laufer@uni-tuebingen.de

#These authors contributed equally:

Florian Wittlinger, Blessing C. Ogboo

NMR Spectra

^{1}H NMR (400 MHz, DMSO) spectrum of compound **S1**

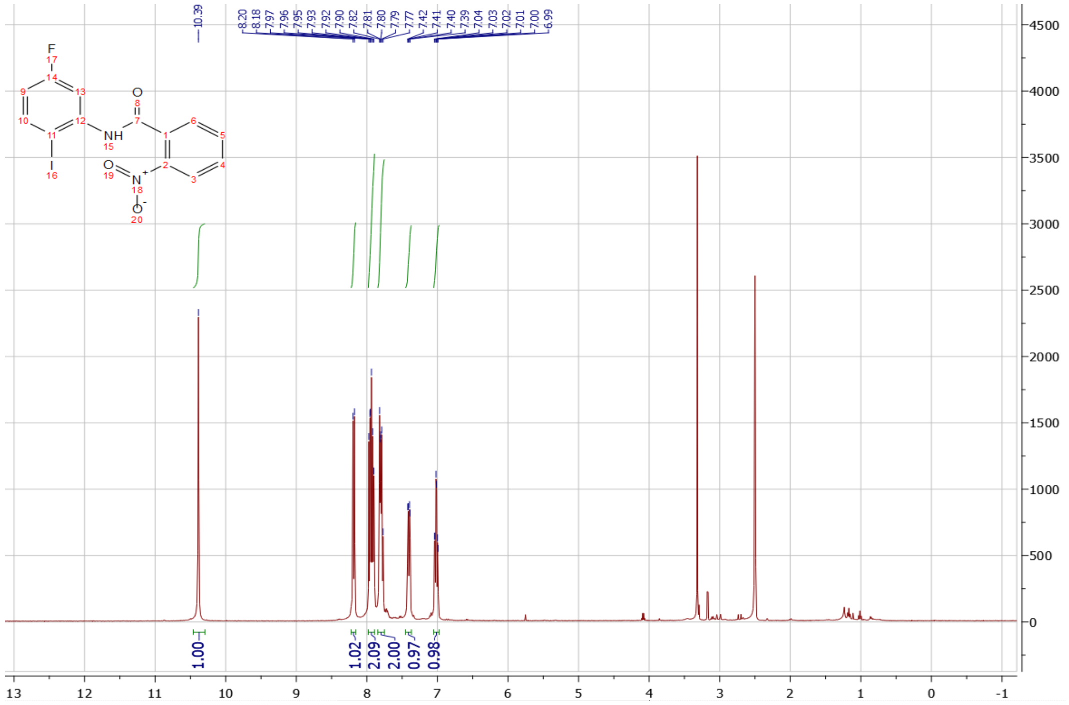

^{13}C NMR (101 MHz, DMSO) spectrum of compound **S1**

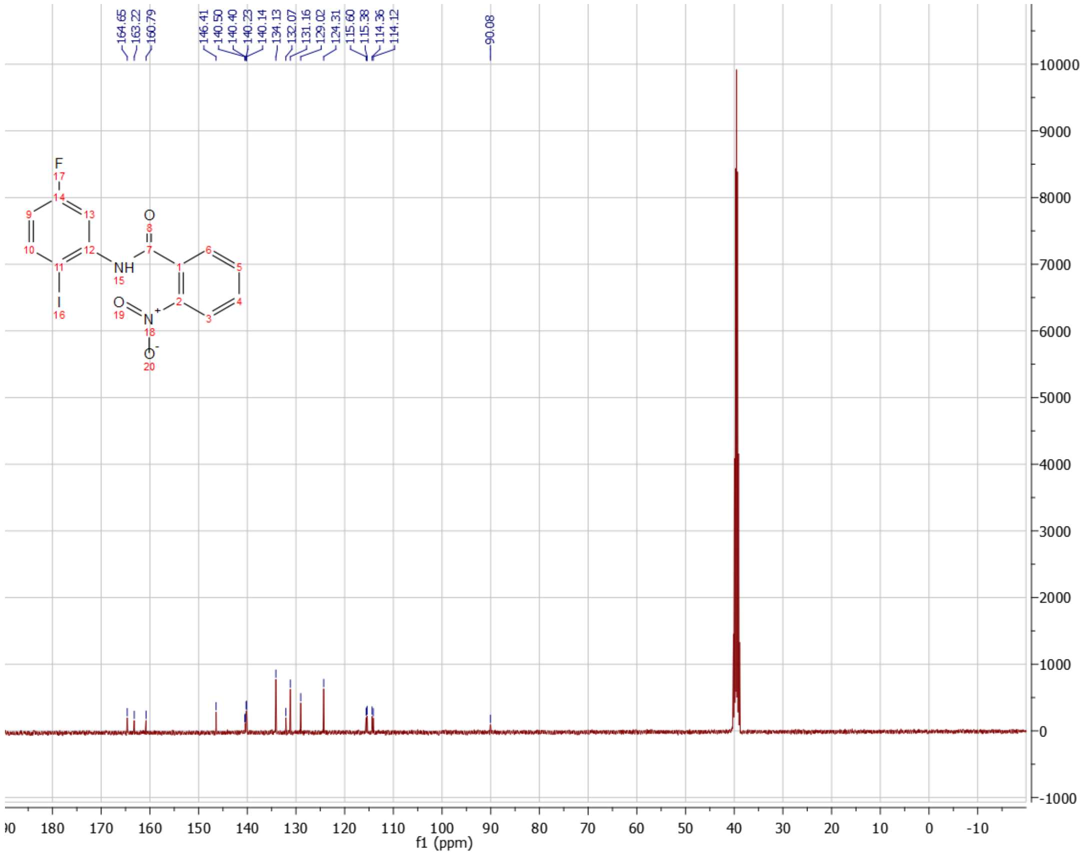

^{1}H NMR (400 MHz, $CDCl_3$) spectrum of compound **S2**

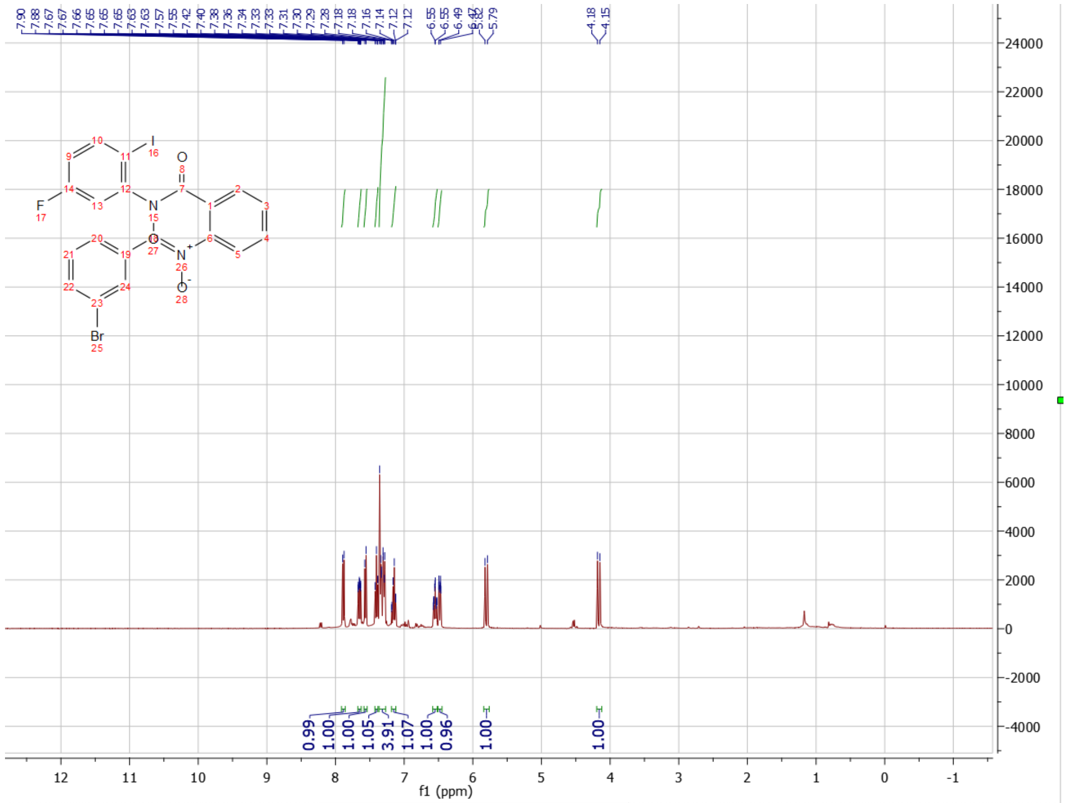

^{13}C NMR (101 MHz, $CDCl_3$) spectrum of compound **S2**

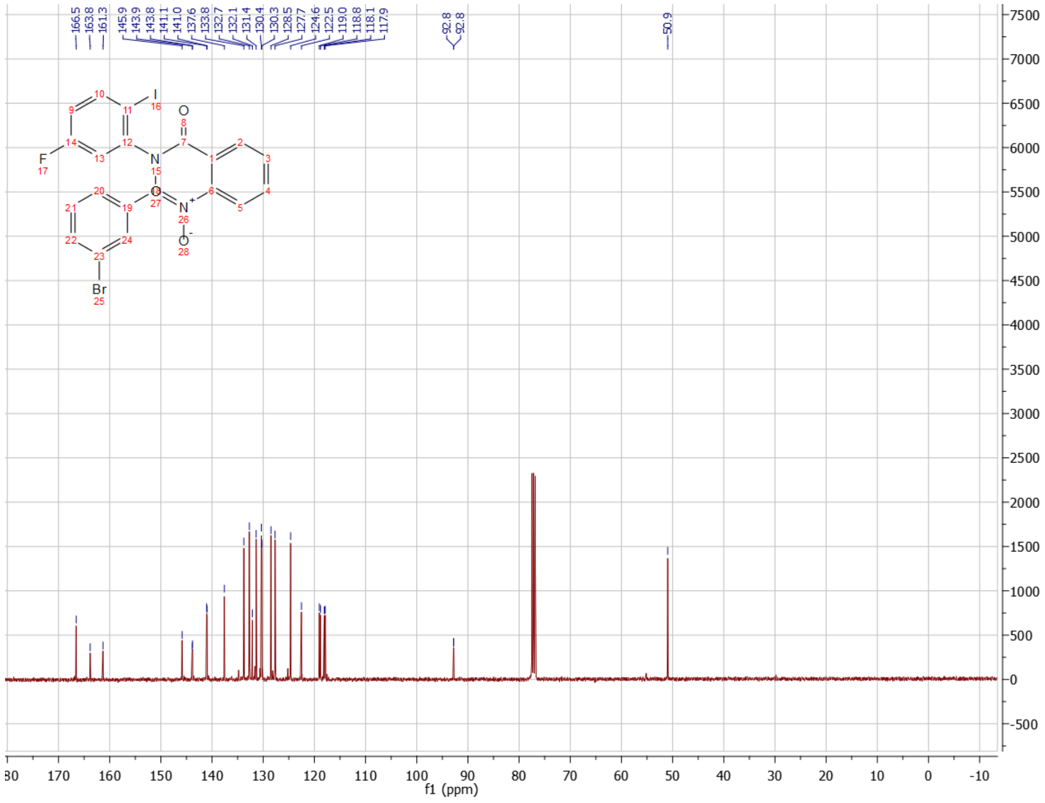

^{1}H NMR (400 MHz, DMSO) spectrum of compound **S4**

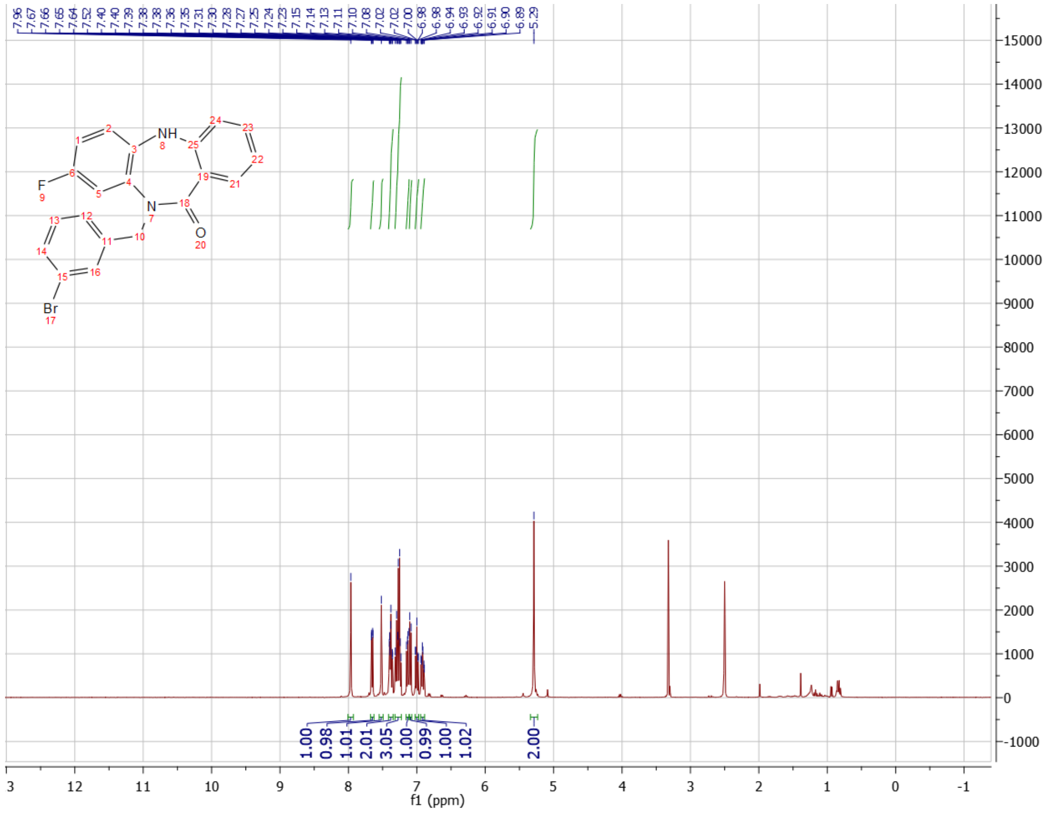

^{13}C NMR (101 MHz, DMSO) spectrum of compound **S4**

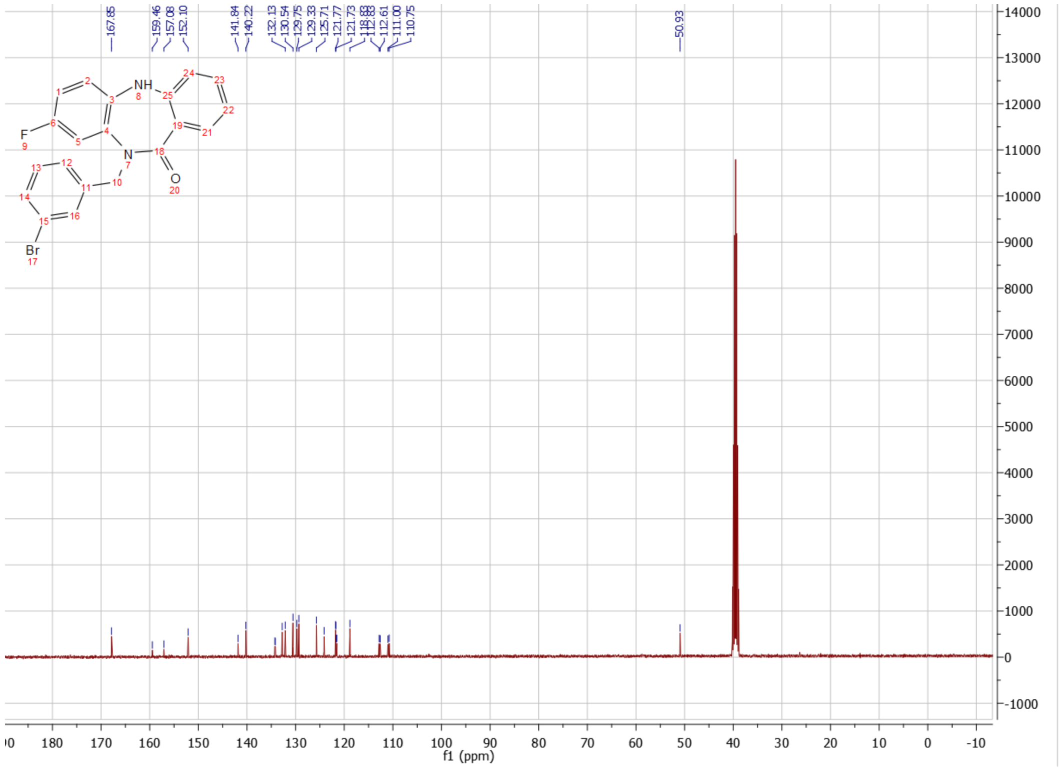

^{1}H NMR (400 MHz, DMSO) spectrum of compound **S6**

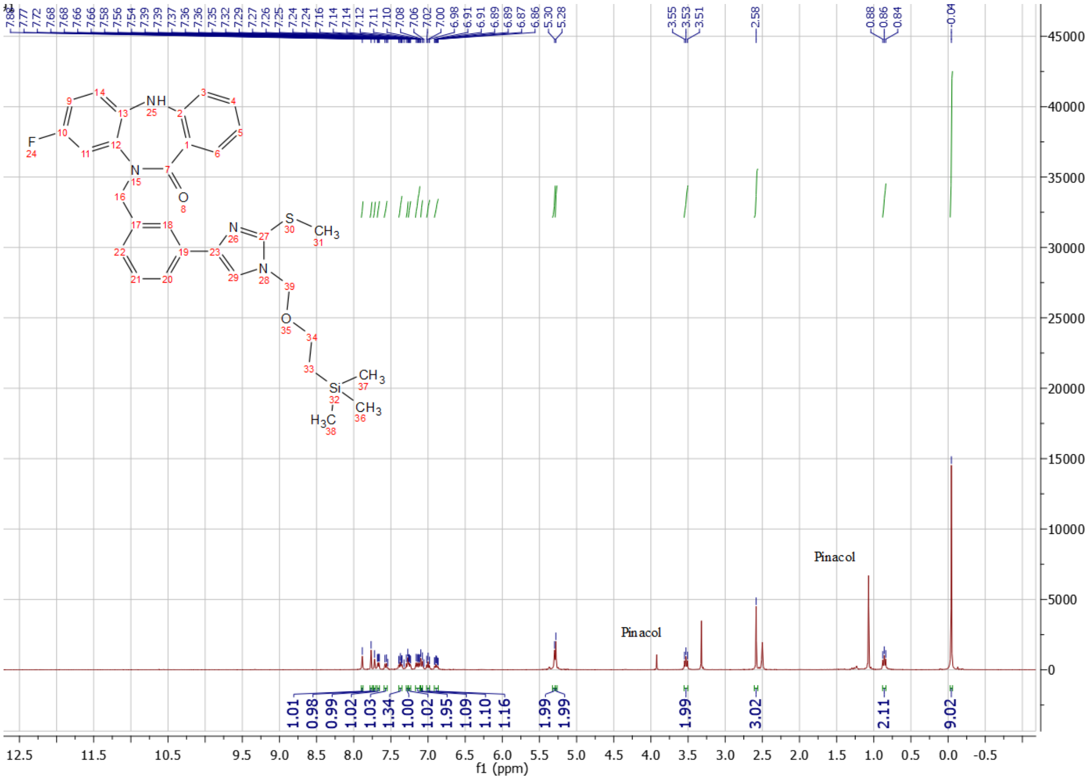

^{13}C NMR (101 MHz, DMSO) spectrum of compound **S6**

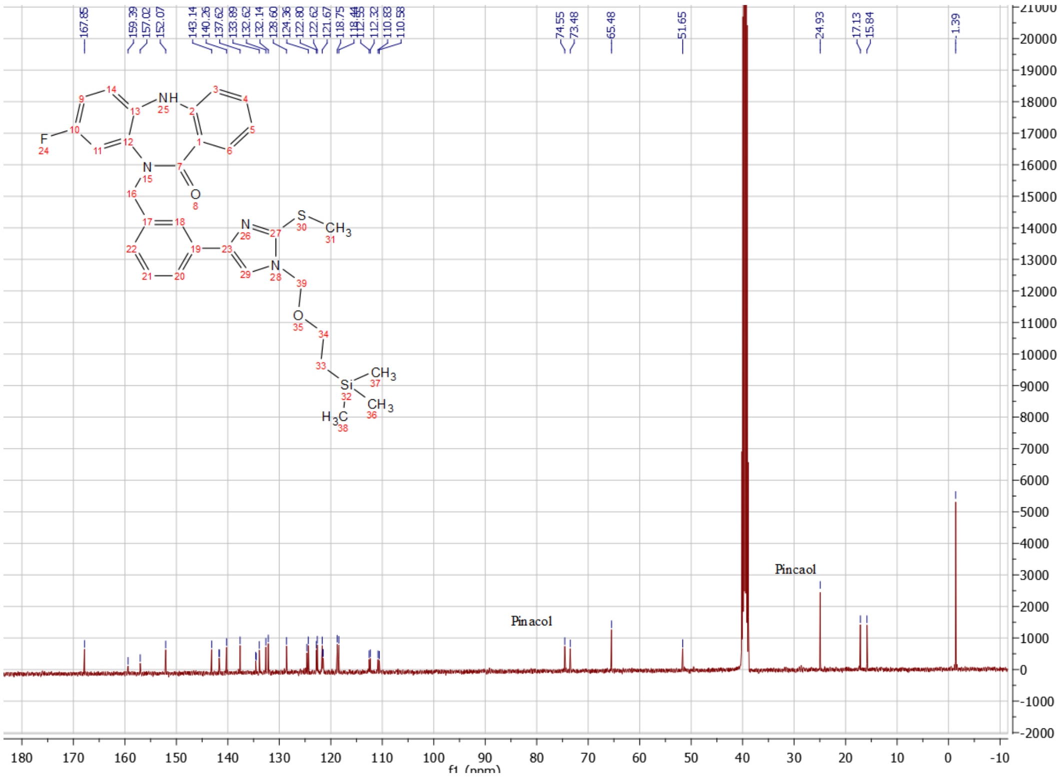

^{1}H NMR (400 MHz, DMSO) spectrum of compound **S7**

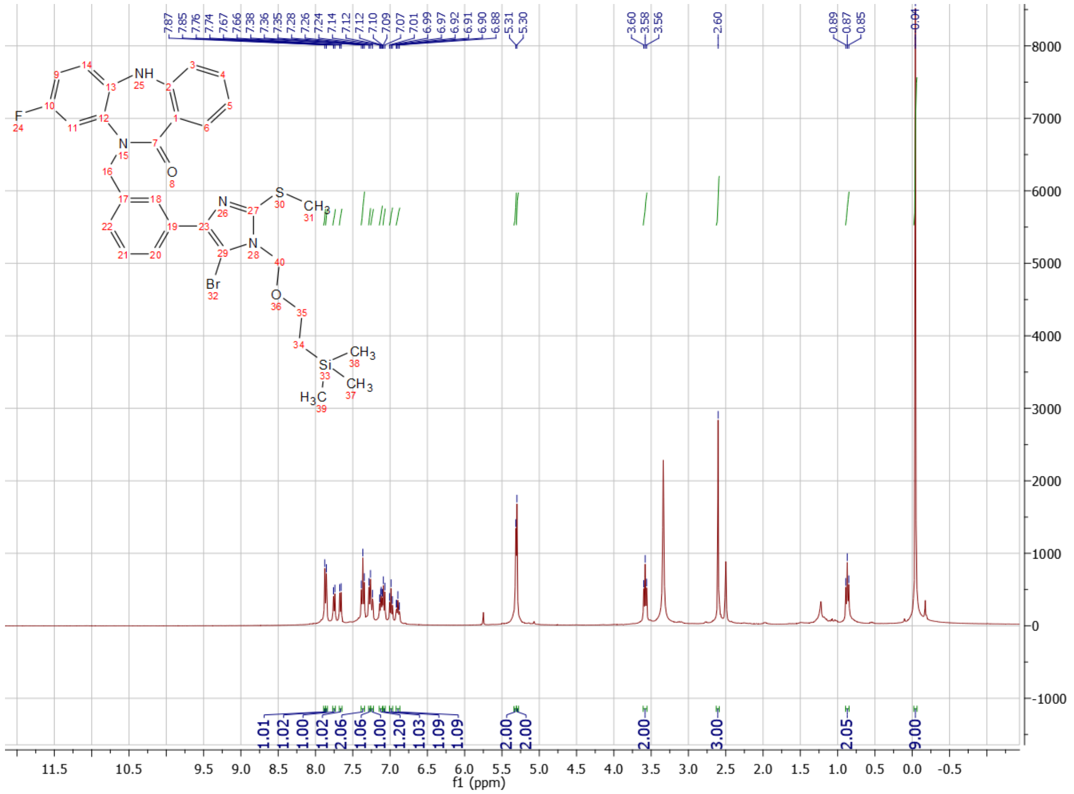

^{13}C NMR (101 MHz, DMSO) spectrum of compound **S7**

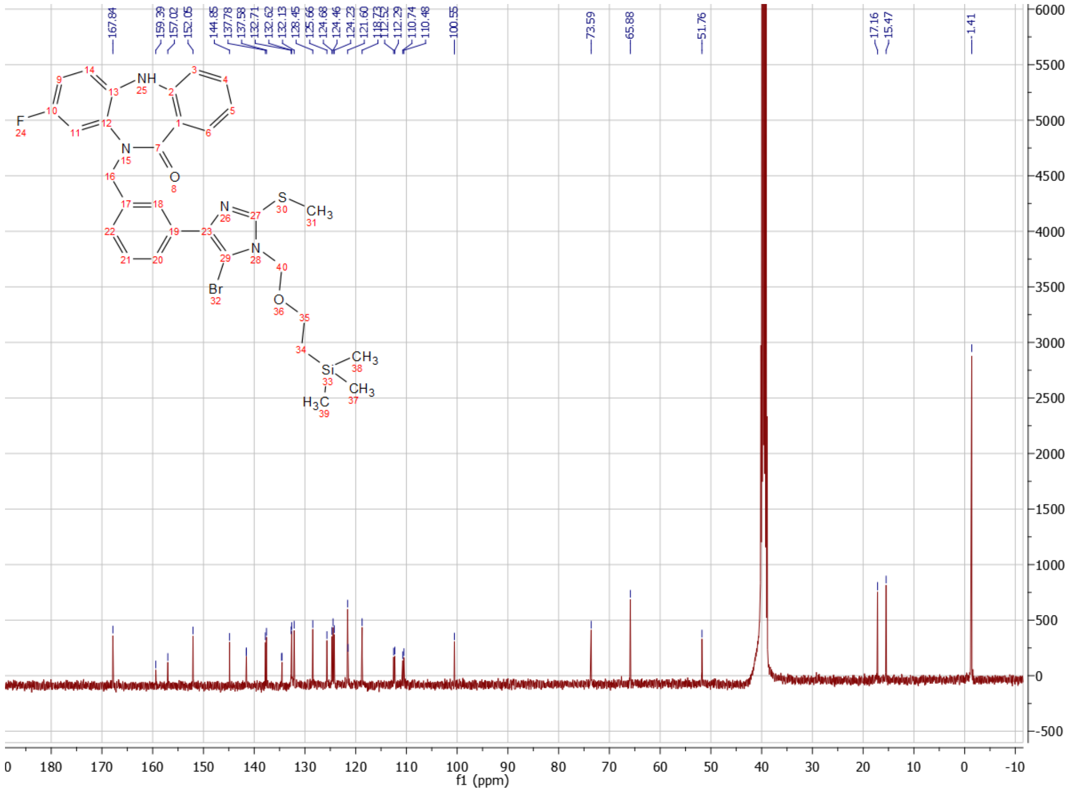

^{1}H NMR (400 MHz, DMSO) spectrum of compound **S8**

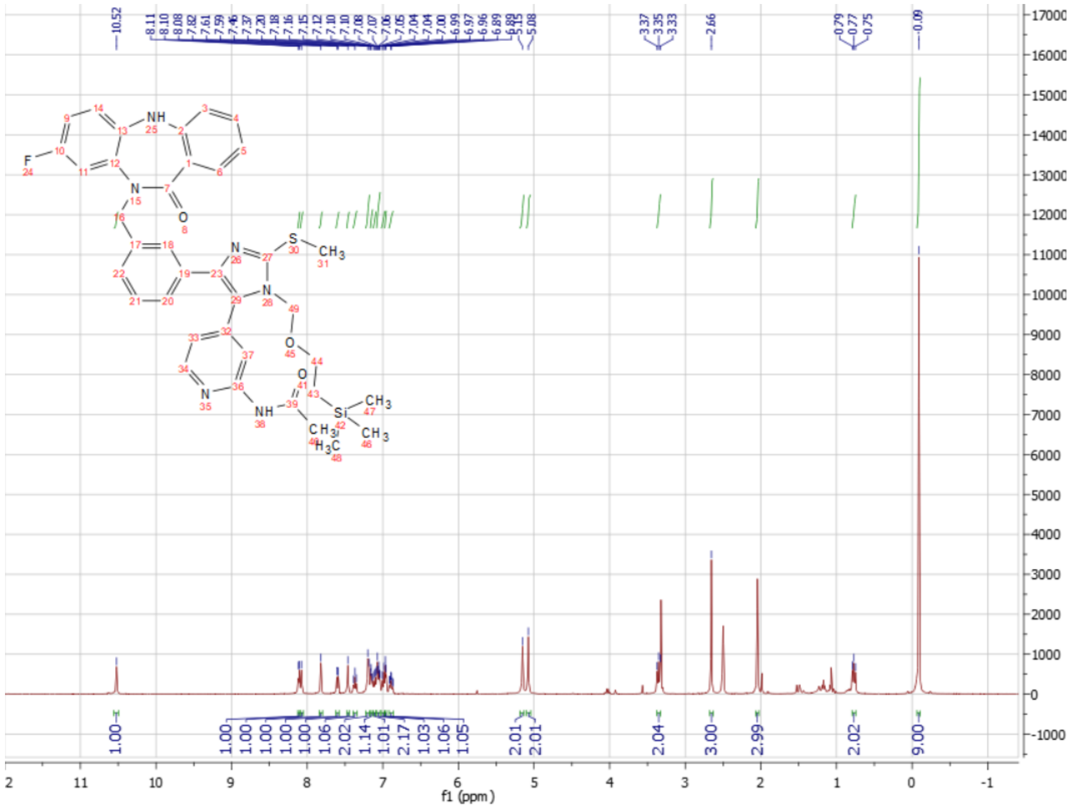

^{13}C NMR (101 MHz, DMSO) spectrum of compound **S8**

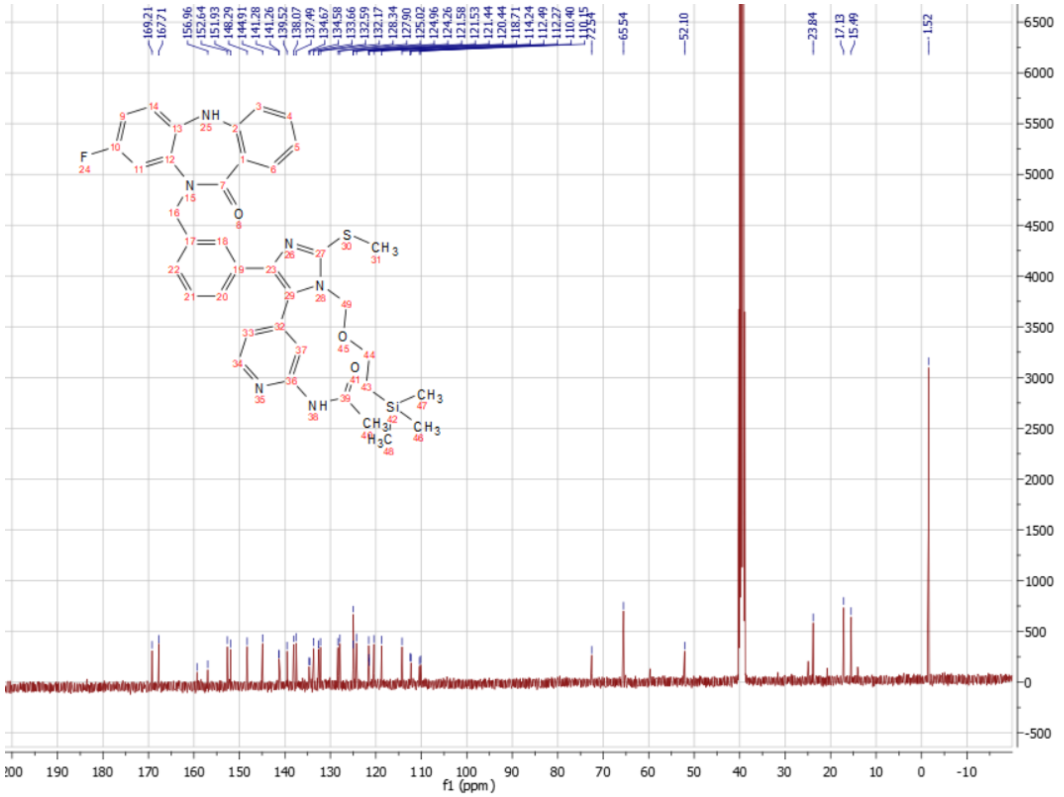

^{1}H NMR (400 MHz, $CDCl_3$) spectrum of compound **S9**

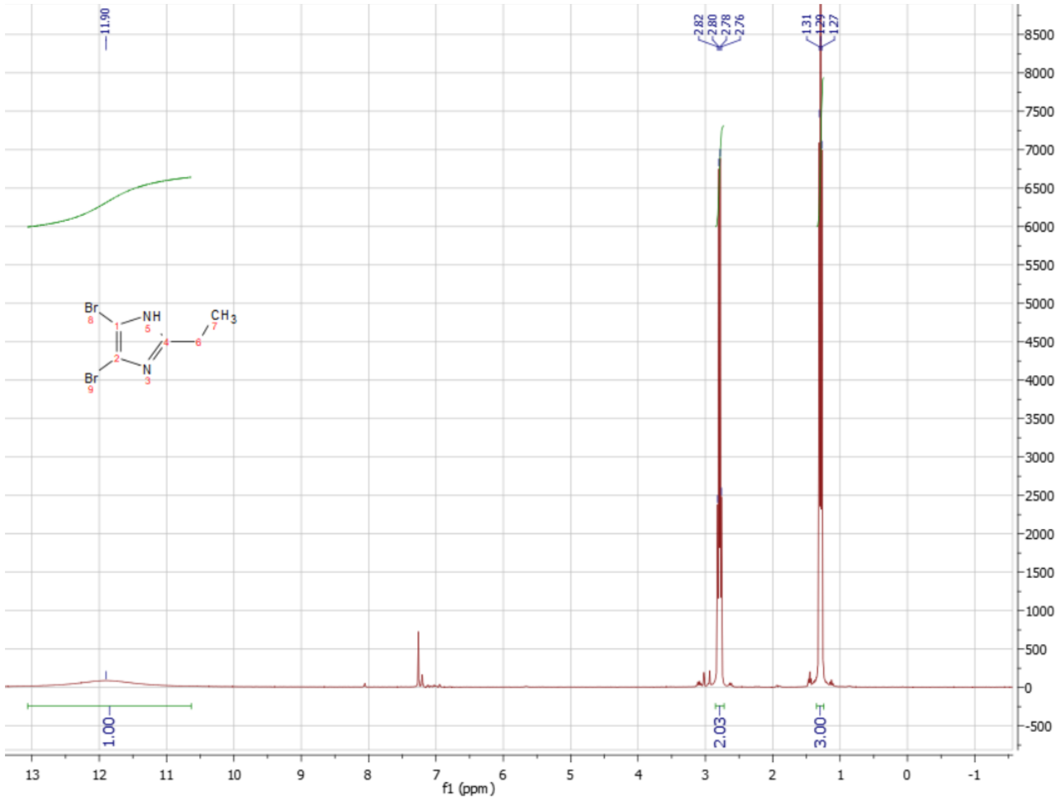

^{13}C NMR (101 MHz, $CDCl_3$) spectrum of compound **S9**

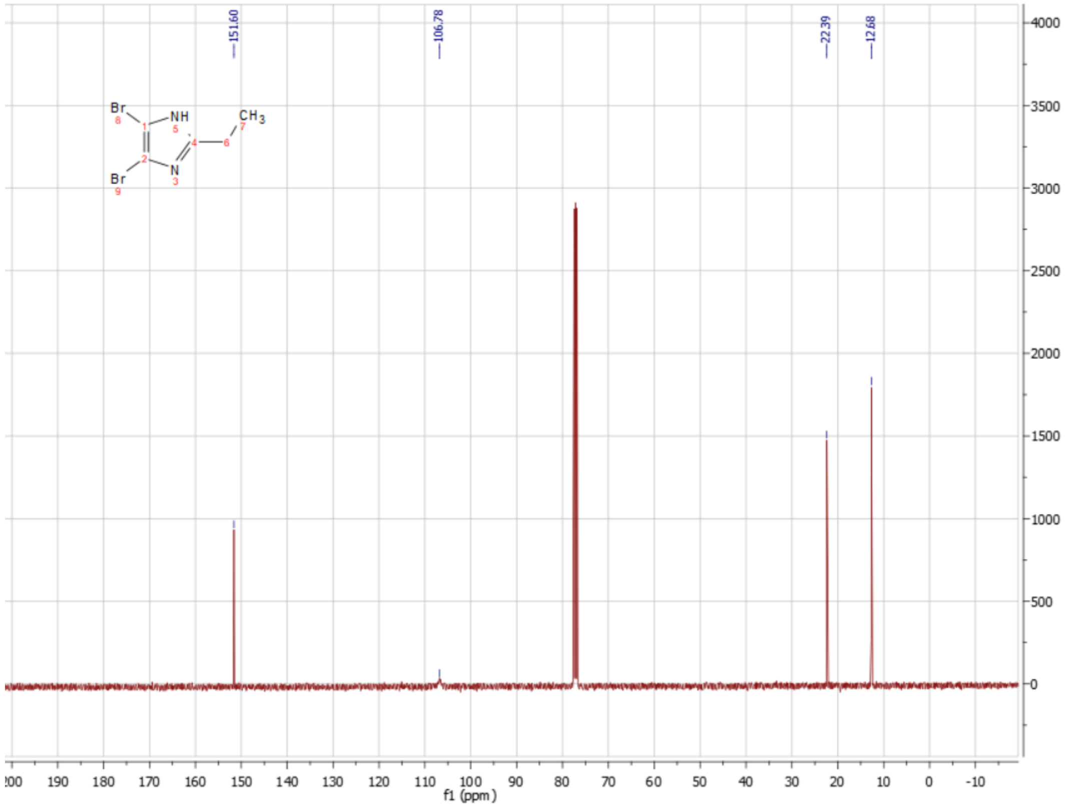

^{1}H NMR (400 MHz, DMSO) spectrum of compound **S10**

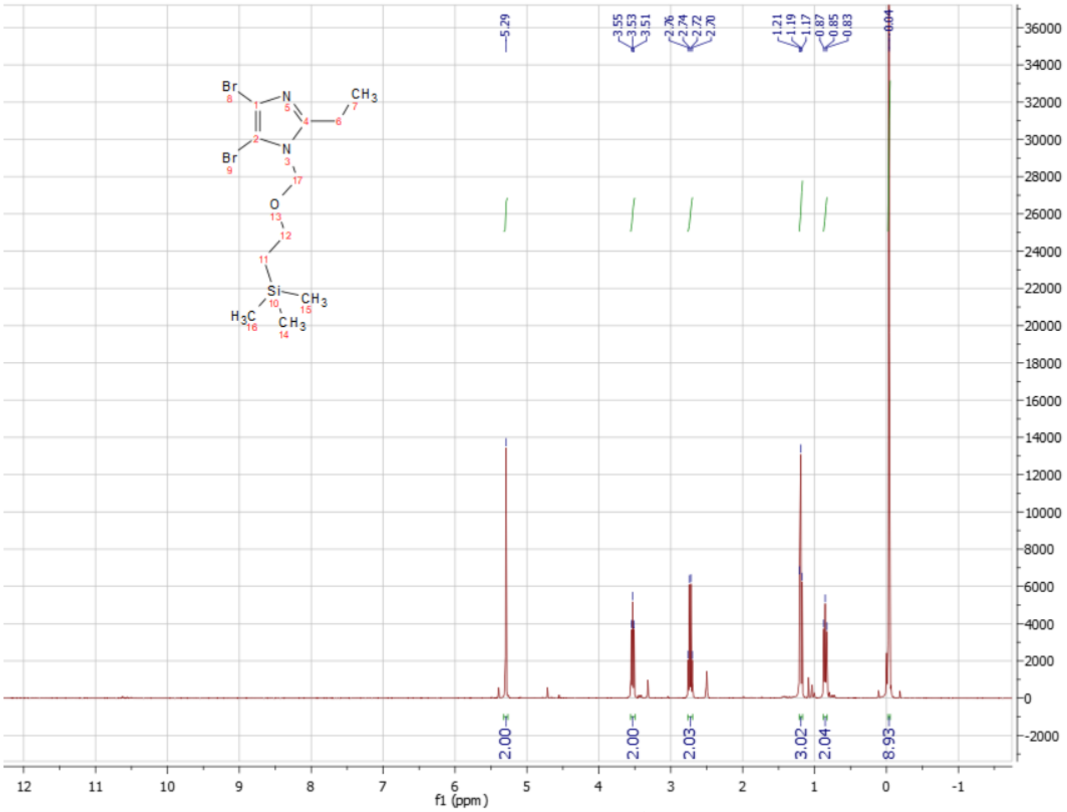

^{13}C NMR (101 MHz, DMSO) spectrum of compound **S10**

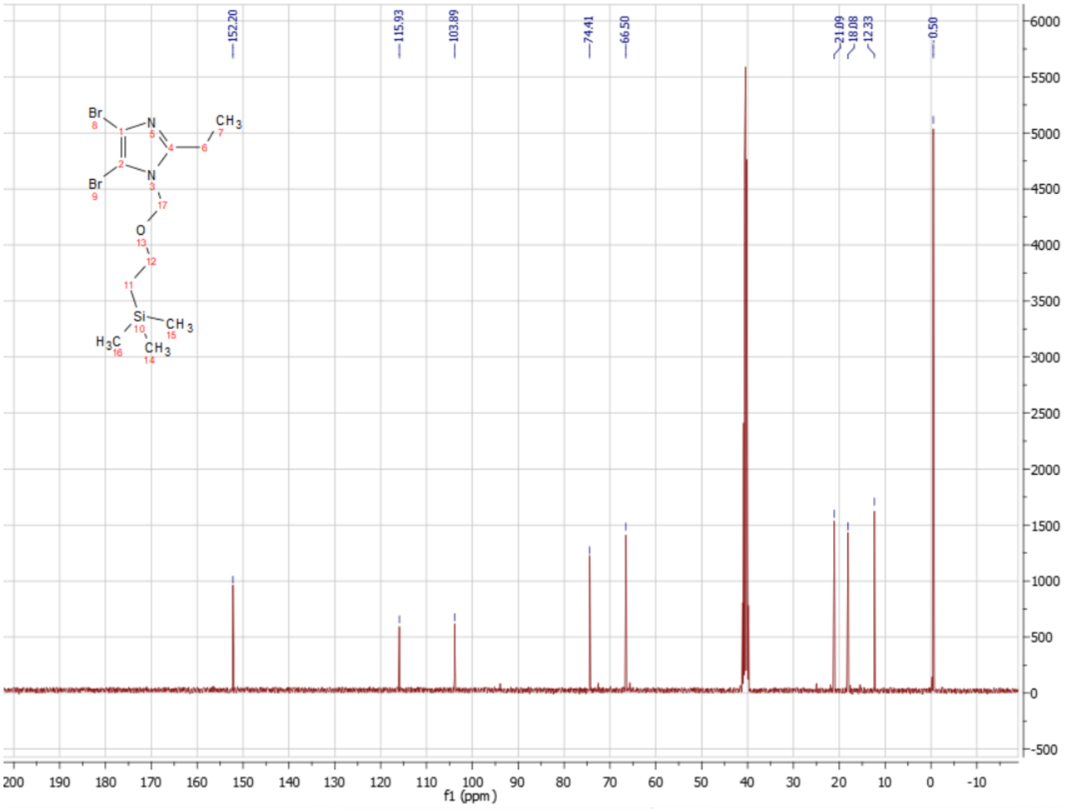

^{1}H NMR (400 MHz, DMSO) spectrum of compound **S11**

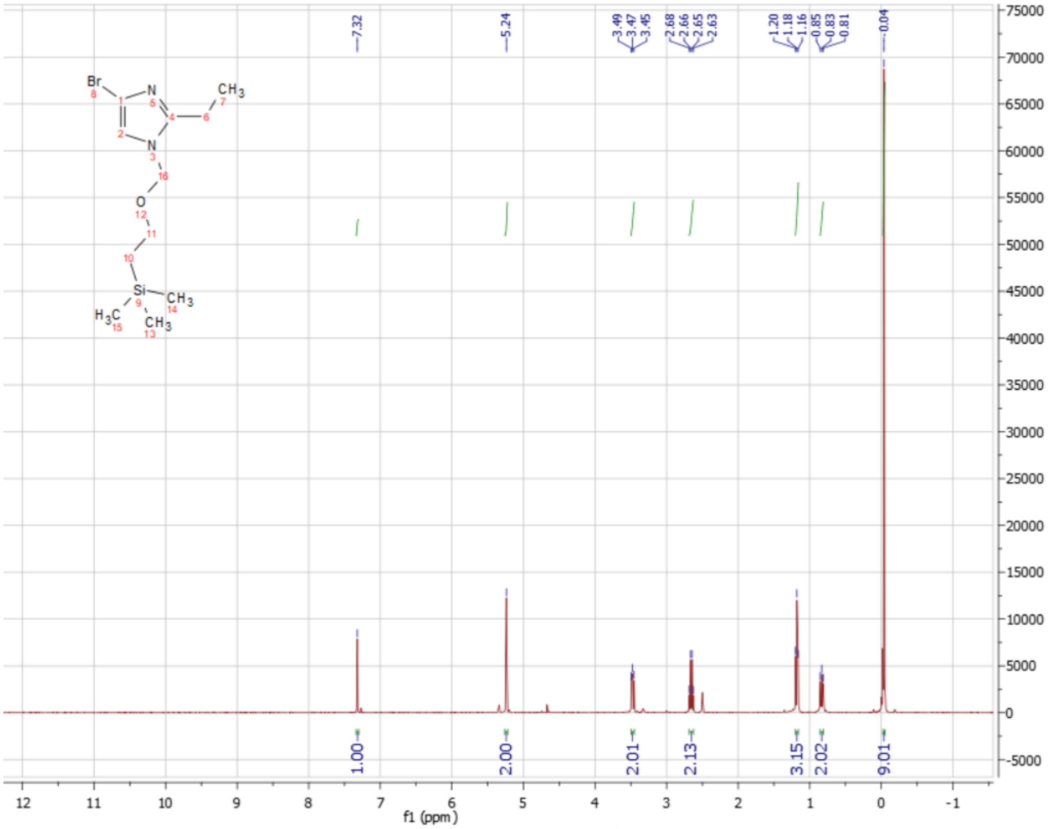

^{13}C NMR (101 MHz, DMSO) spectrum of compound **S11**

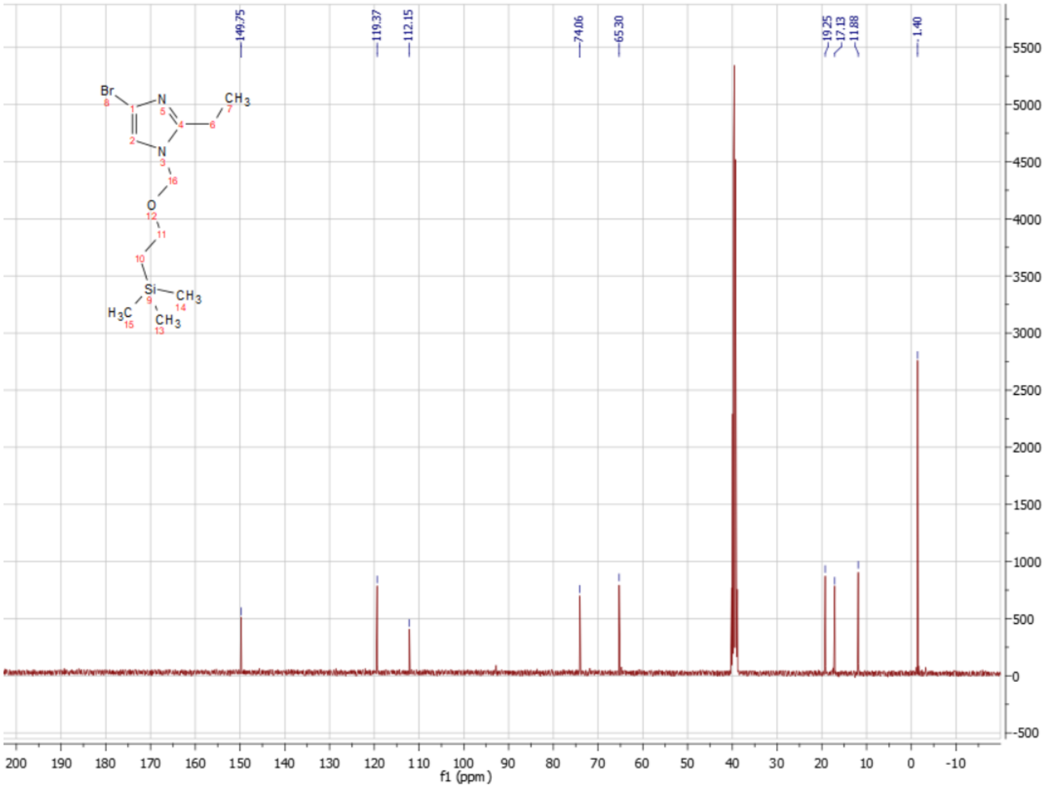

^{1}H NMR (400 MHz, DMSO) spectrum of compound **S12**

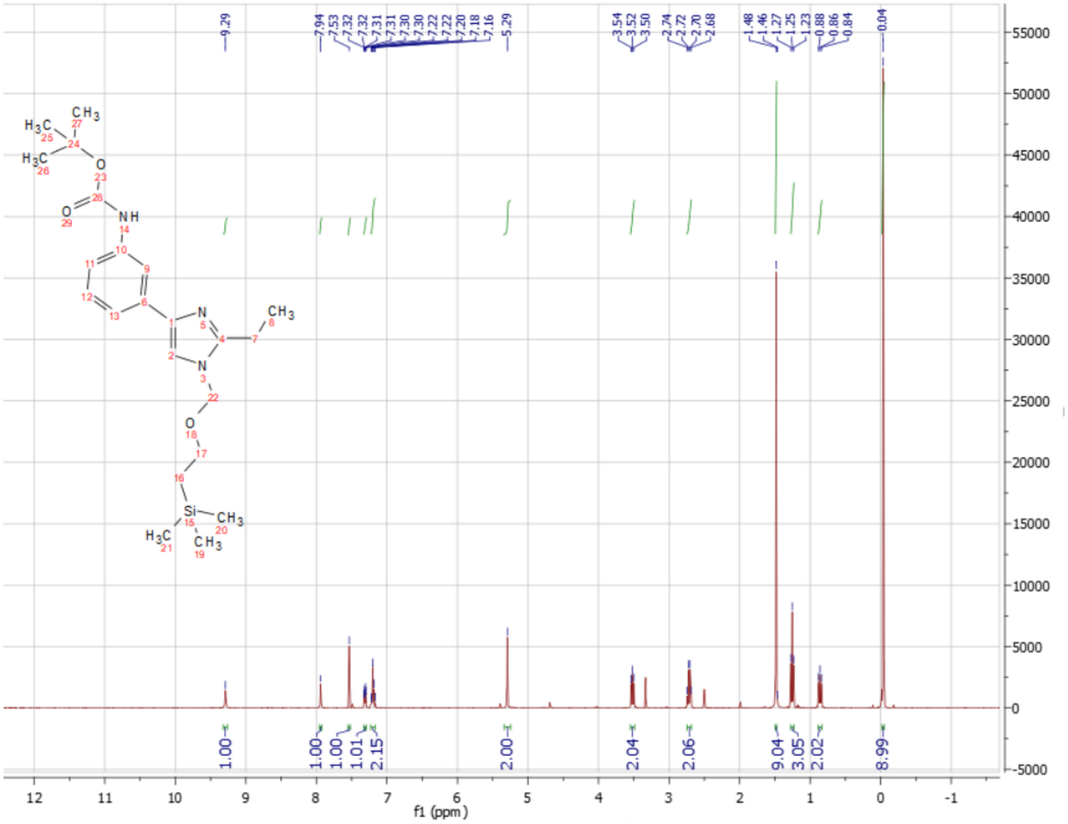

^{13}C NMR (101 MHz, DMSO) spectrum of compound **S12**

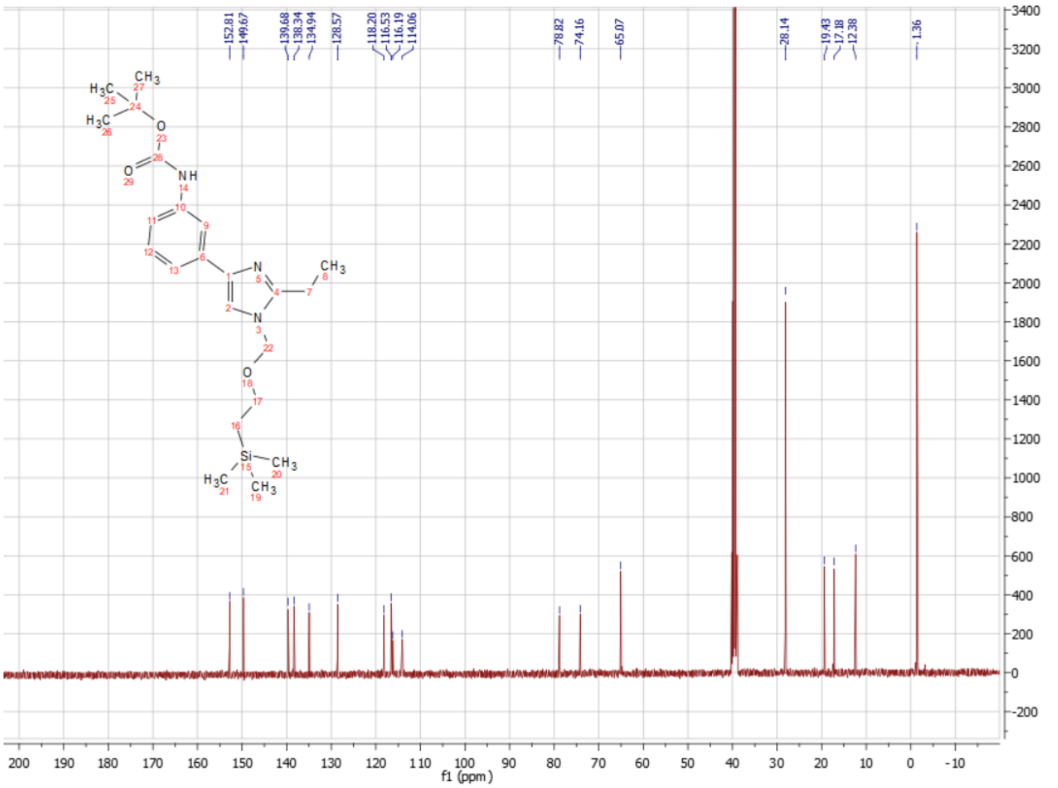

^{1}H NMR (400 MHz, DMSO) spectrum of compound **S13**

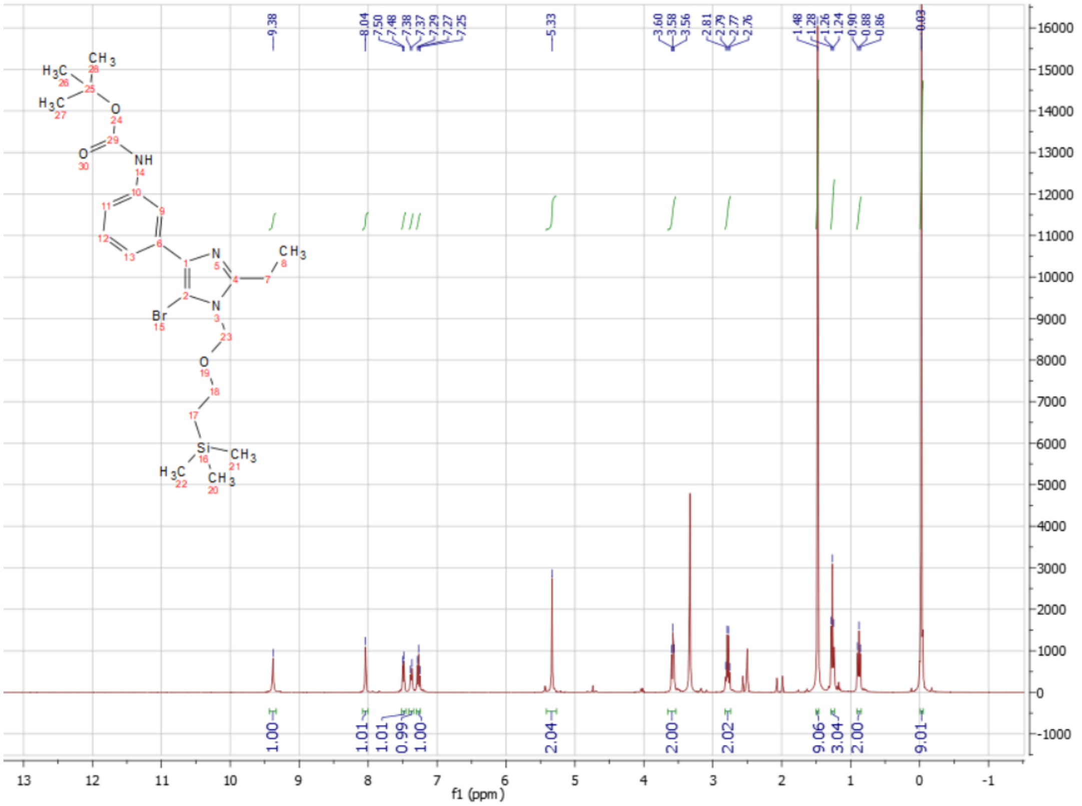

^{13}C NMR (101 MHz, DMSO) spectrum of compound **S13**

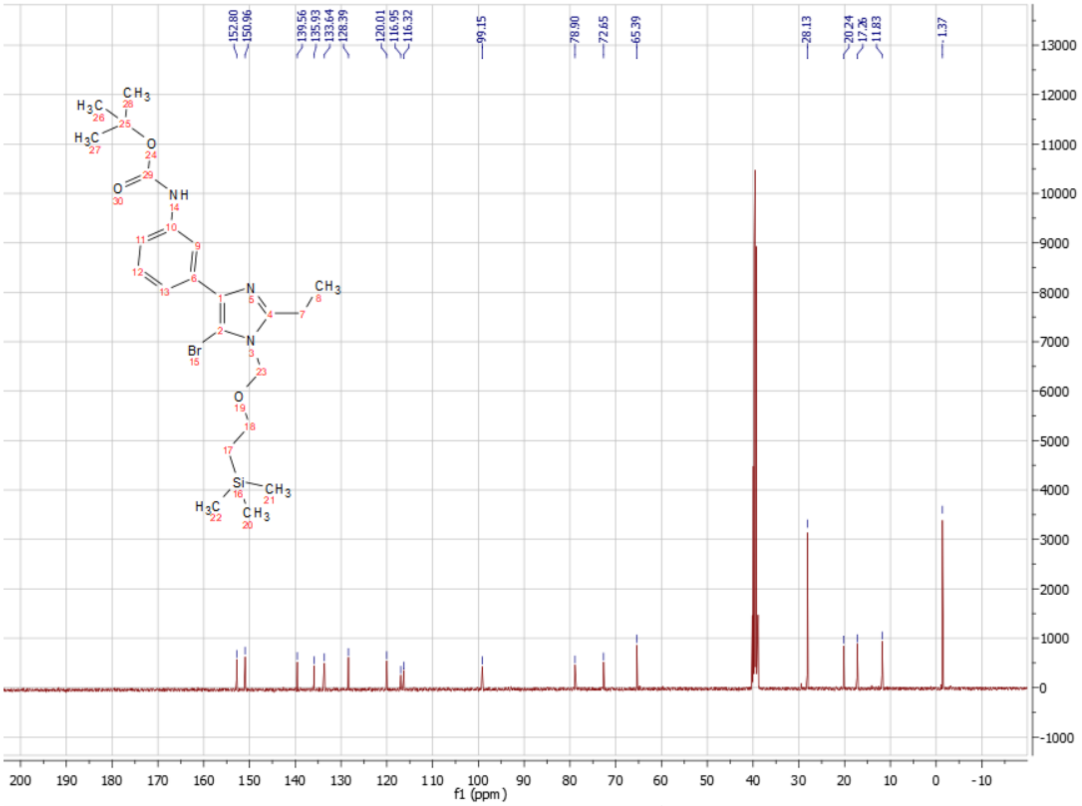

^{1}H NMR (400 MHz, DMSO) spectrum of compound **S14**

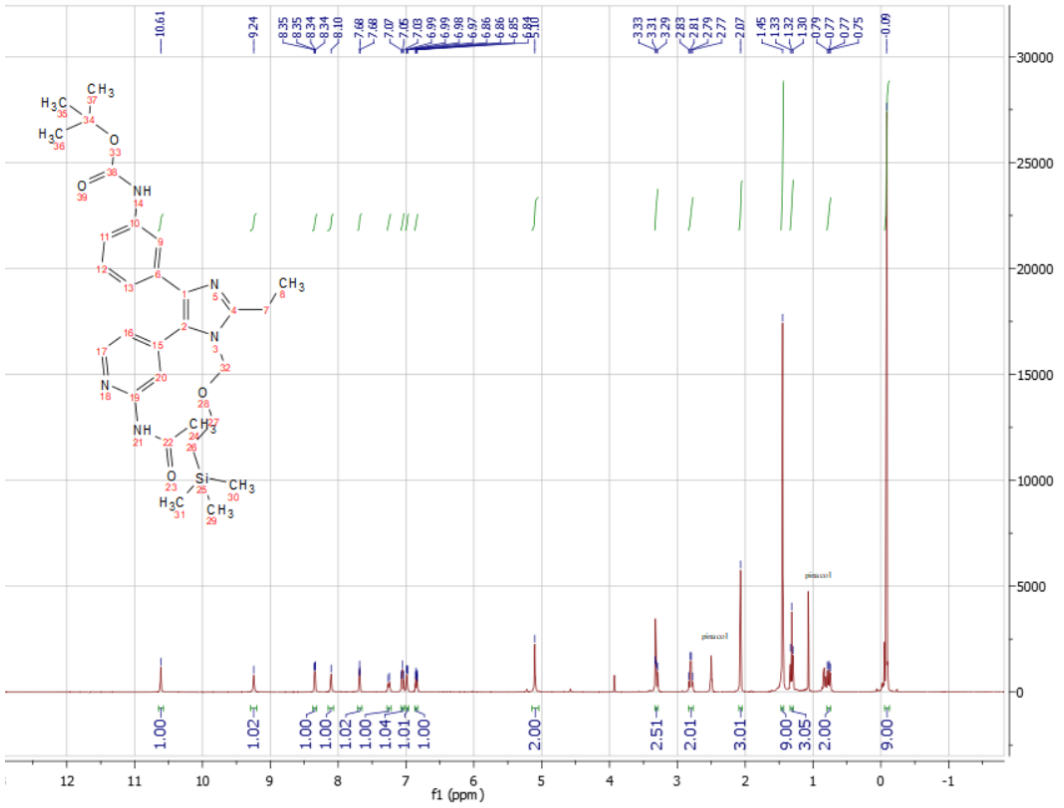

^{13}C NMR (101 MHz, DMSO) spectrum of compound **S14**

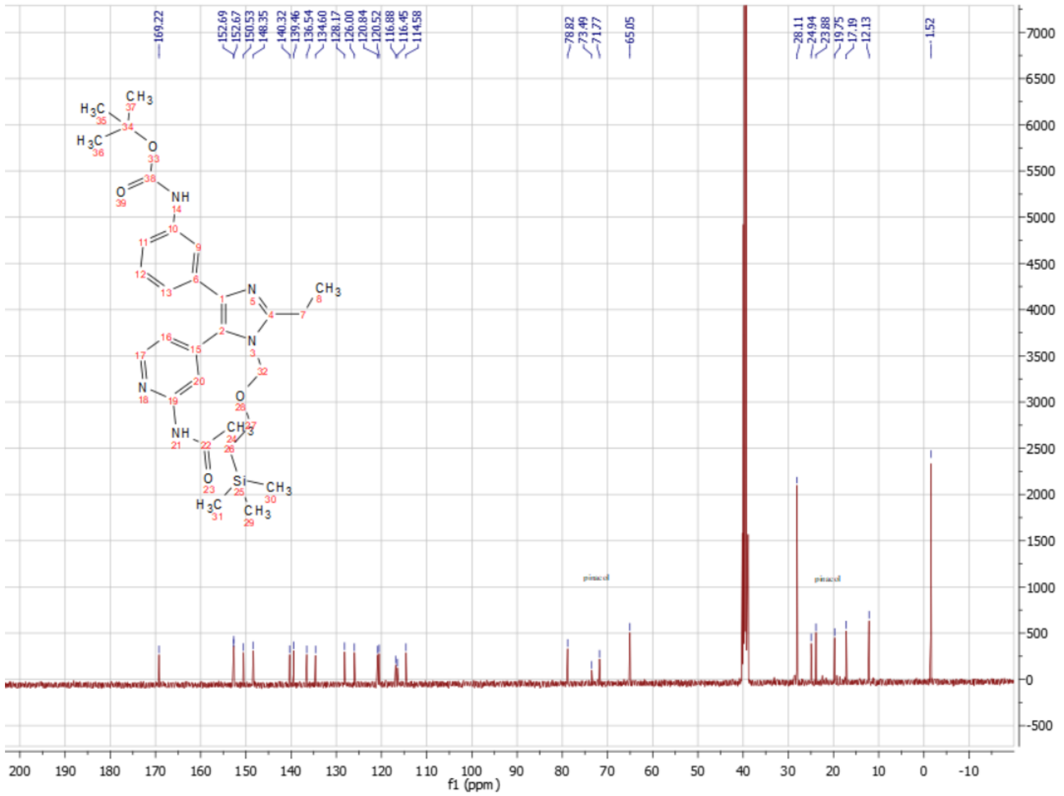

^{1}H NMR (400 MHz, DMSO) spectrum of compound **S15**

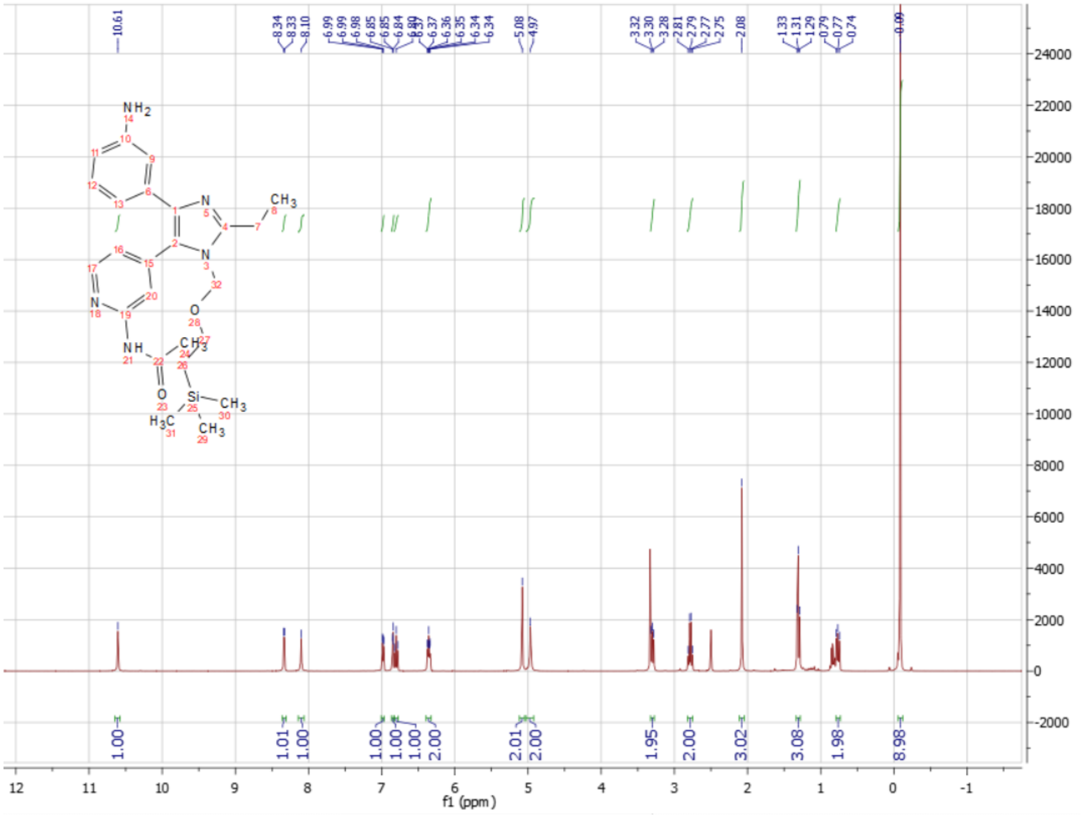

^{13}C NMR (101 MHz, DMSO) spectrum of compound **S15**

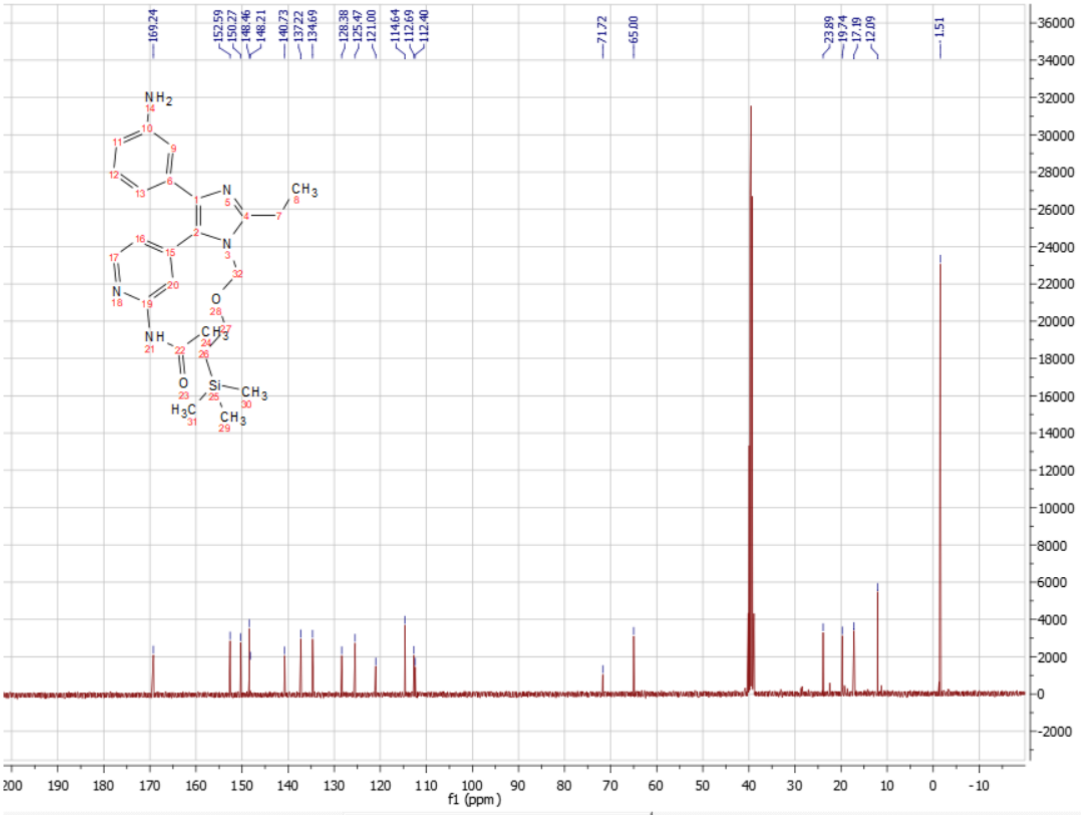

^{1}H NMR (400 MHz, DMSO) spectrum of compound **S16**

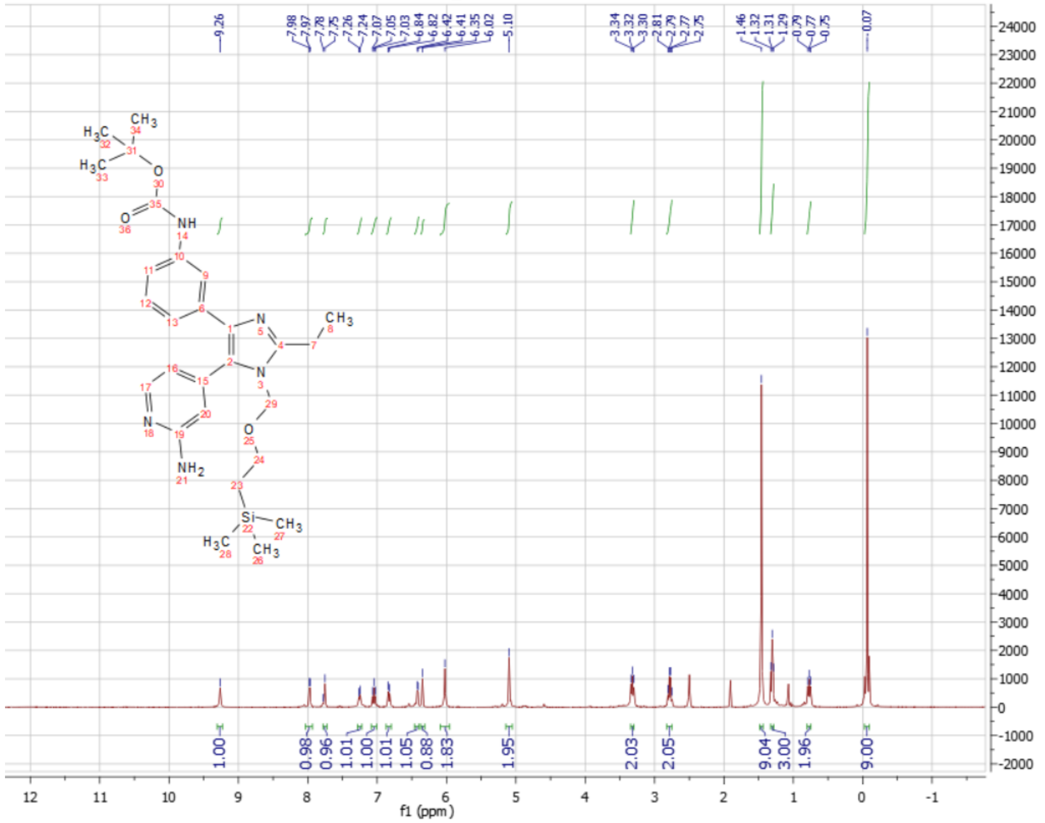

^{13}C NMR (101 MHz, DMSO) spectrum of compound **S16**

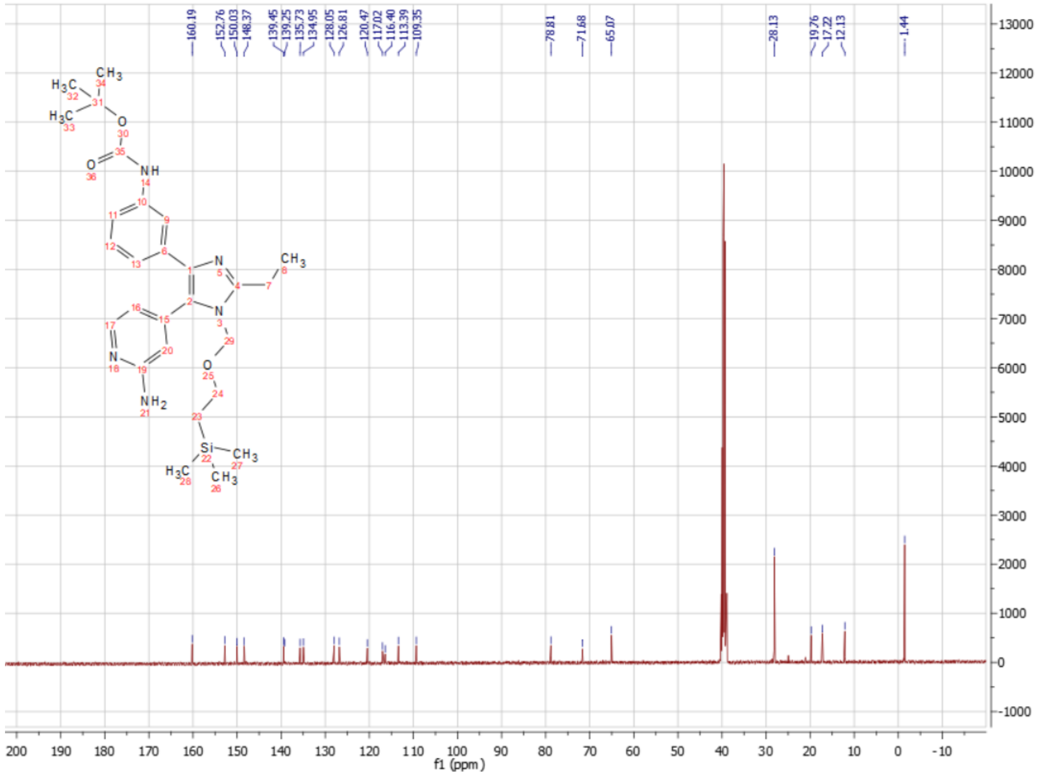

^{1}H NMR (400 MHz, DMSO) spectrum of compound **S17**

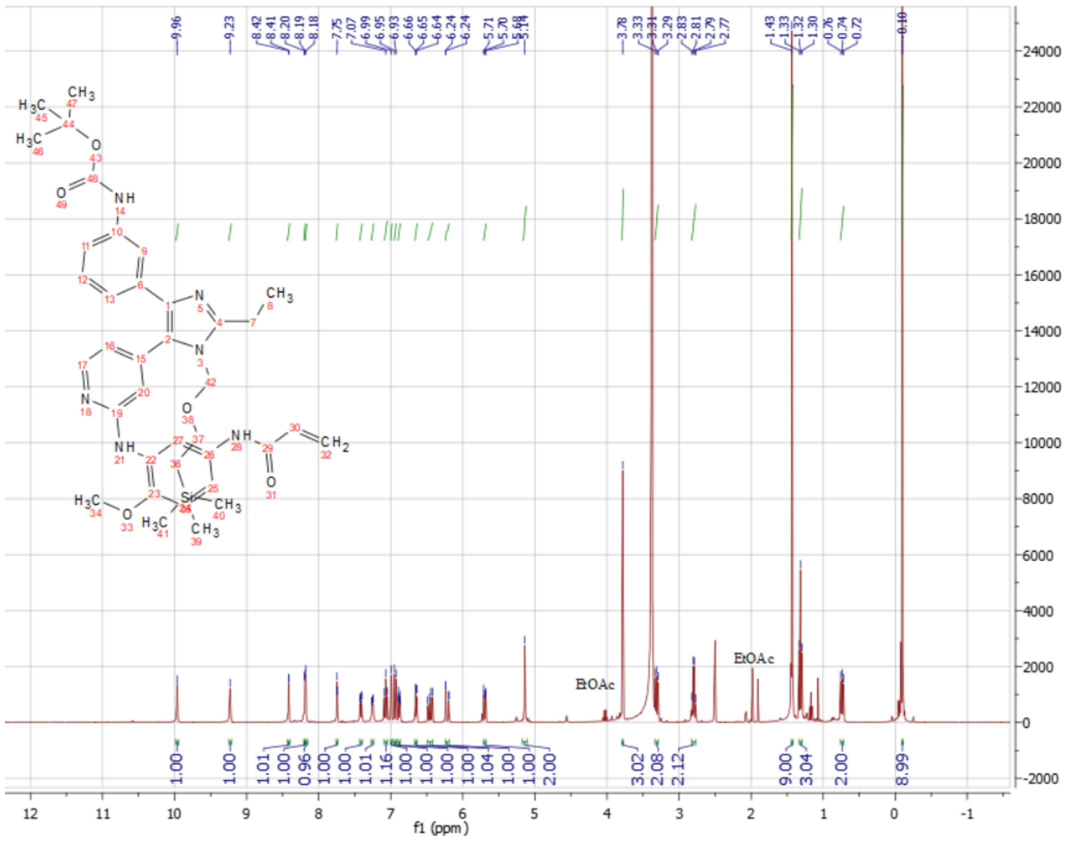

^{13}C NMR (101 MHz, DMSO) spectrum of compound **S17**

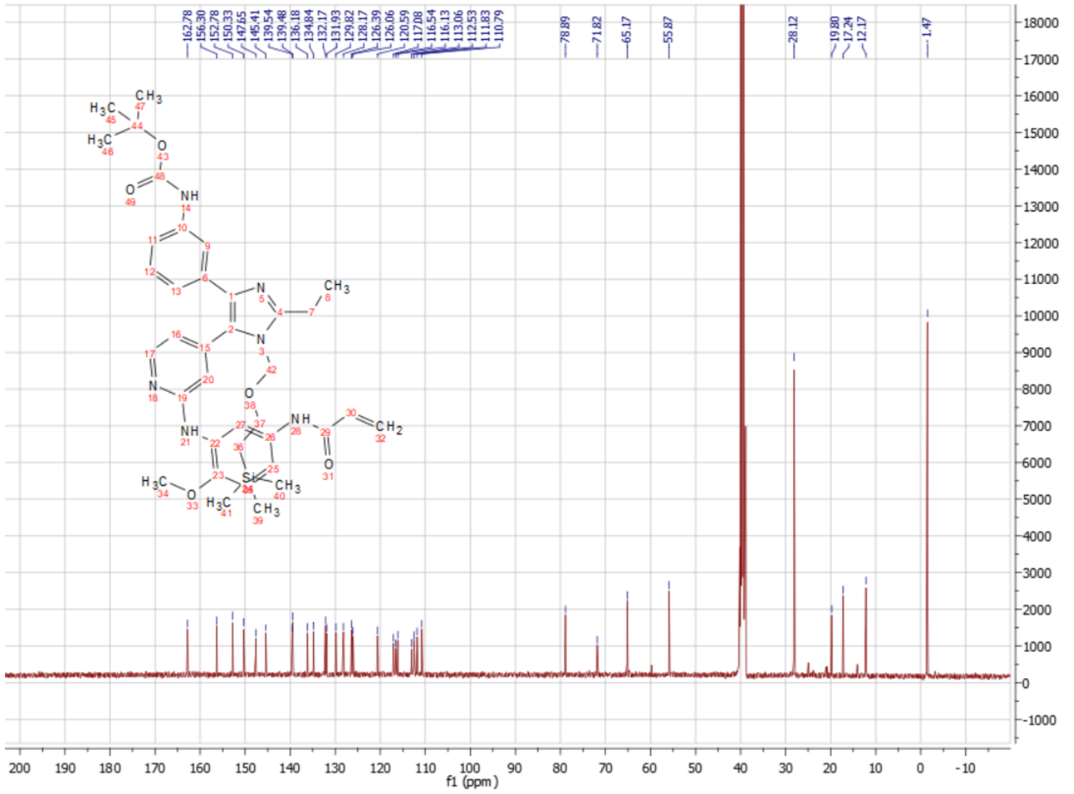

^{1}H NMR (400 MHz, DMSO) spectrum of compound **S18**

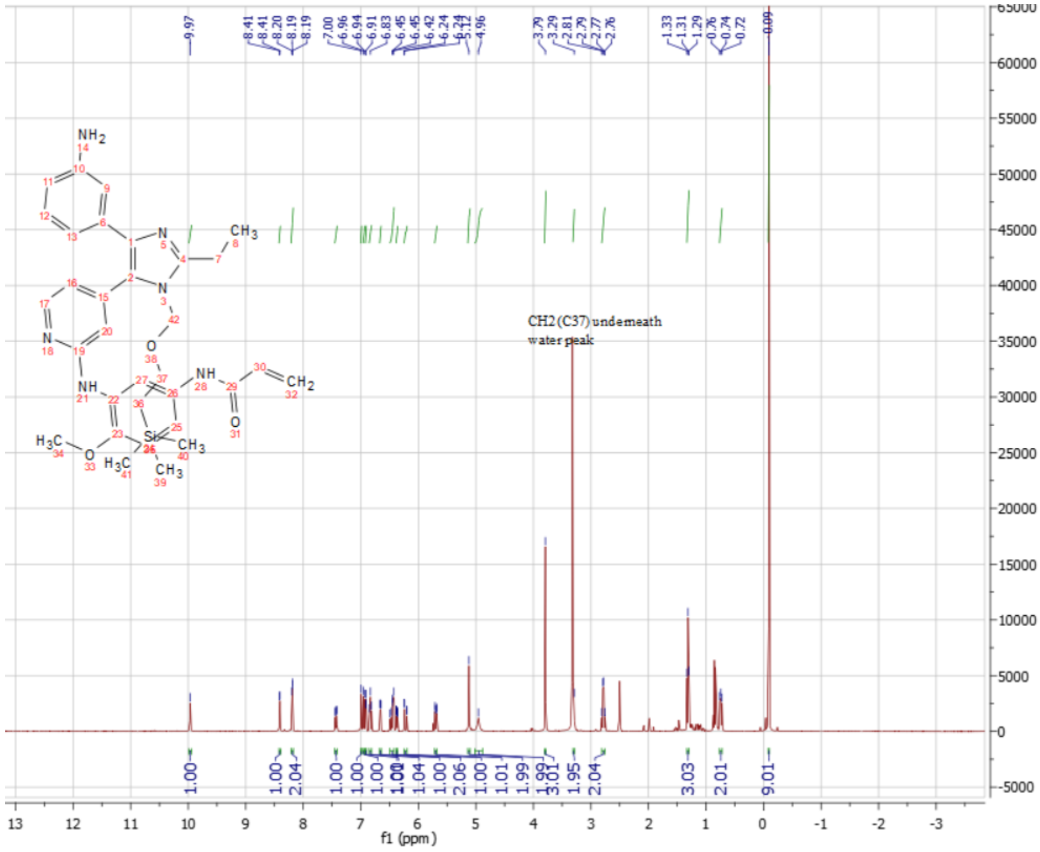

^{13}C NMR (101 MHz, DMSO) spectrum of compound **S18**

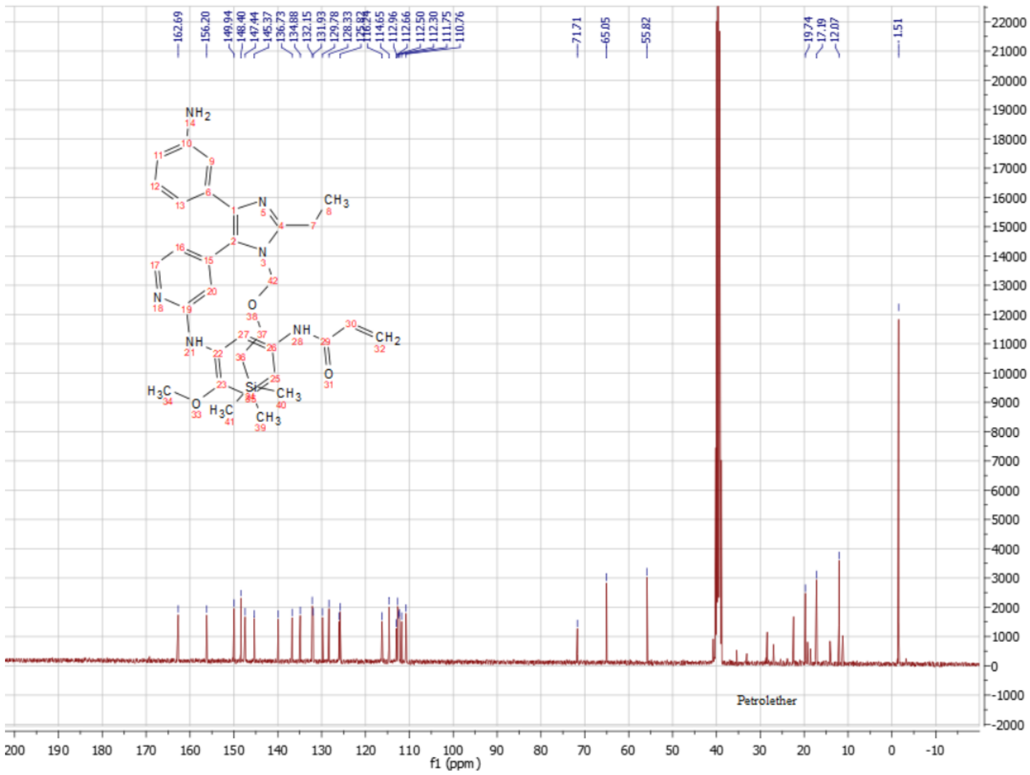

^1H NMR (400 MHz, DMSO) spectrum of compound **S19**

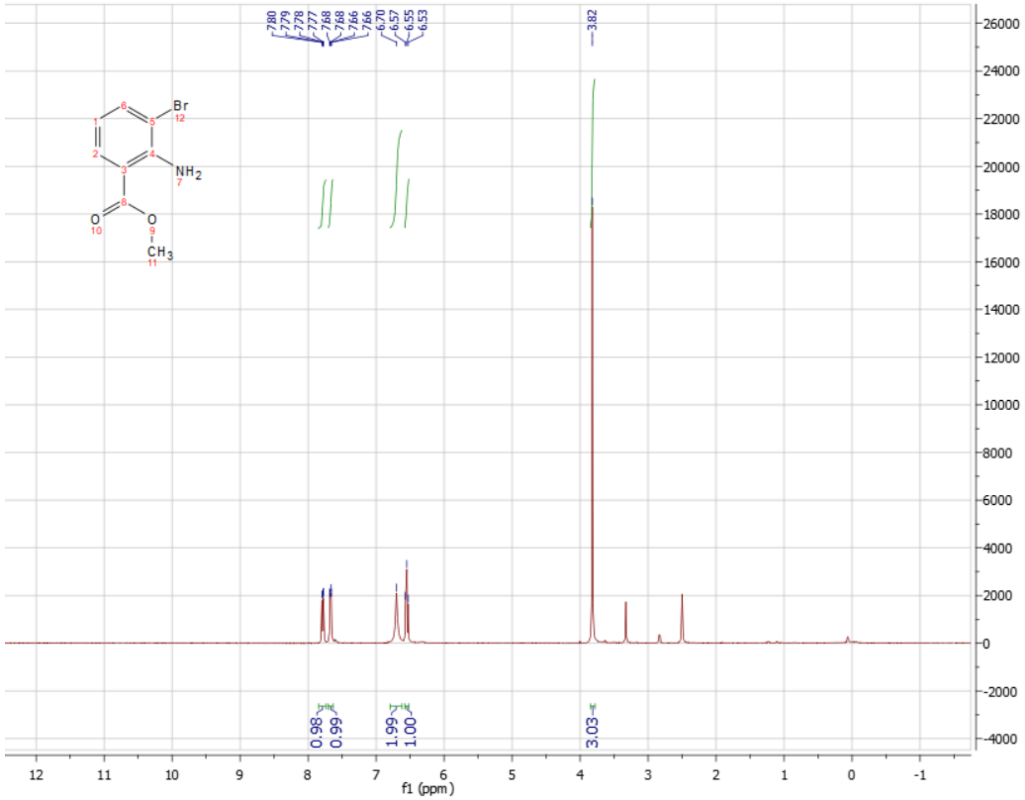

^{13}C NMR (101 MHz, DMSO) spectrum of compound **S19**

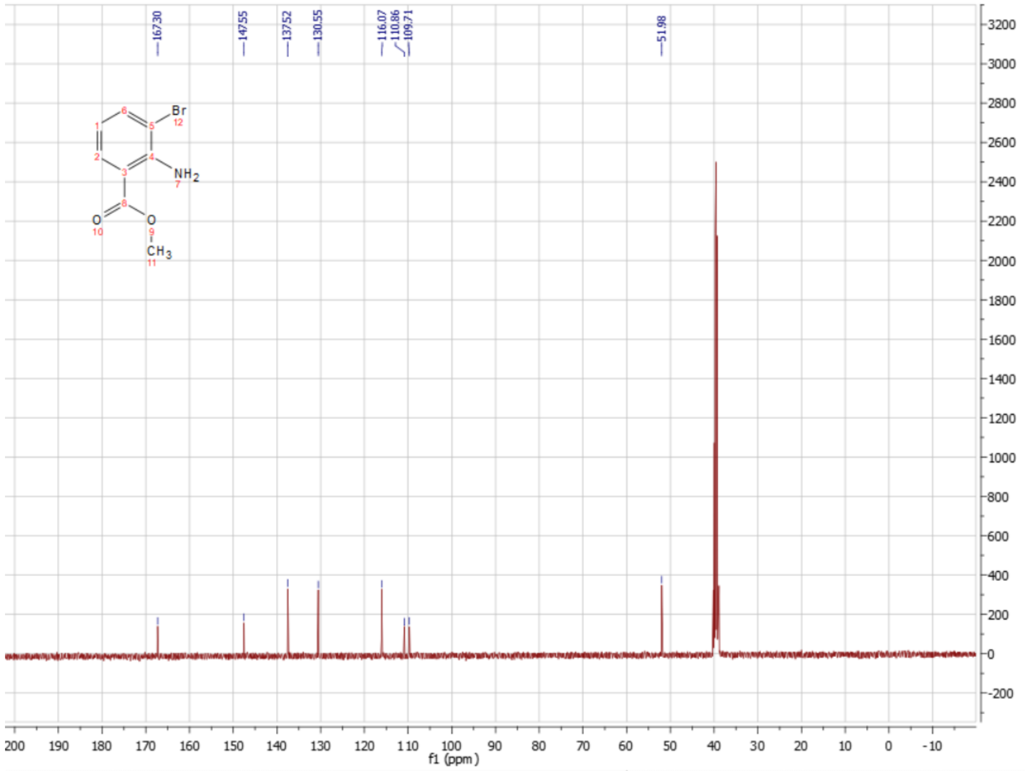

^{1}H NMR (400 MHz, DMSO) spectrum of compound **S20**

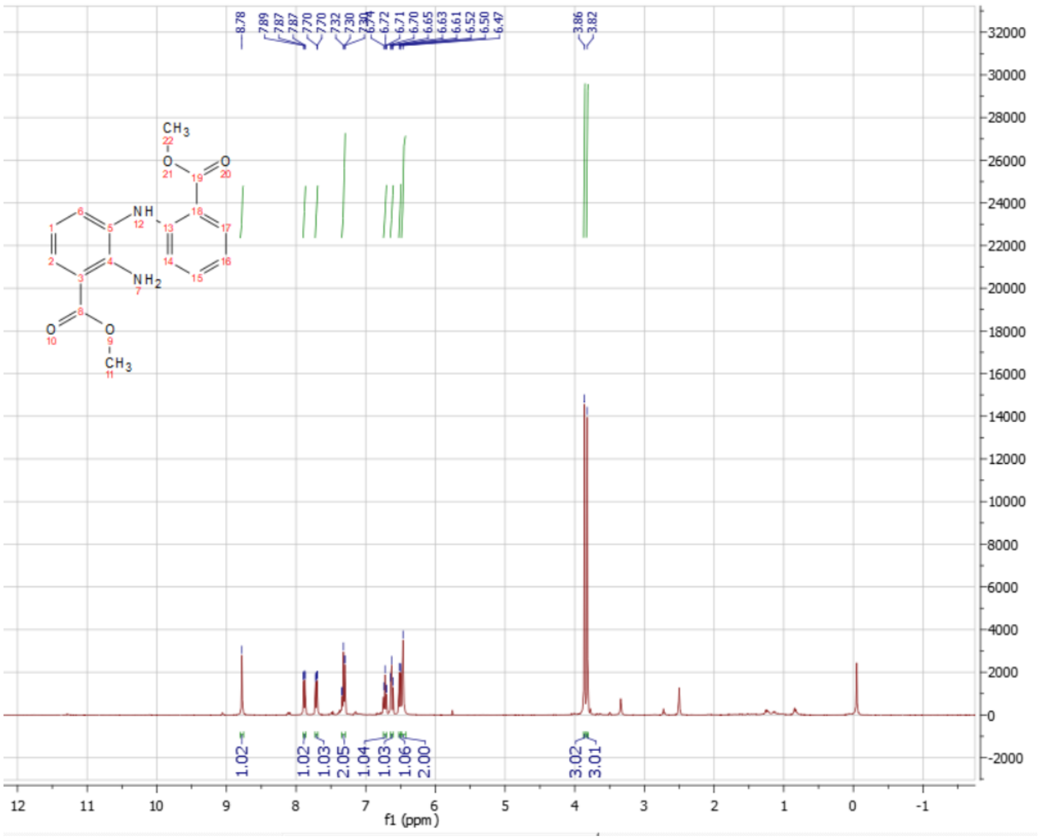

^{13}C NMR (101 MHz, DMSO) spectrum of compound **S20**

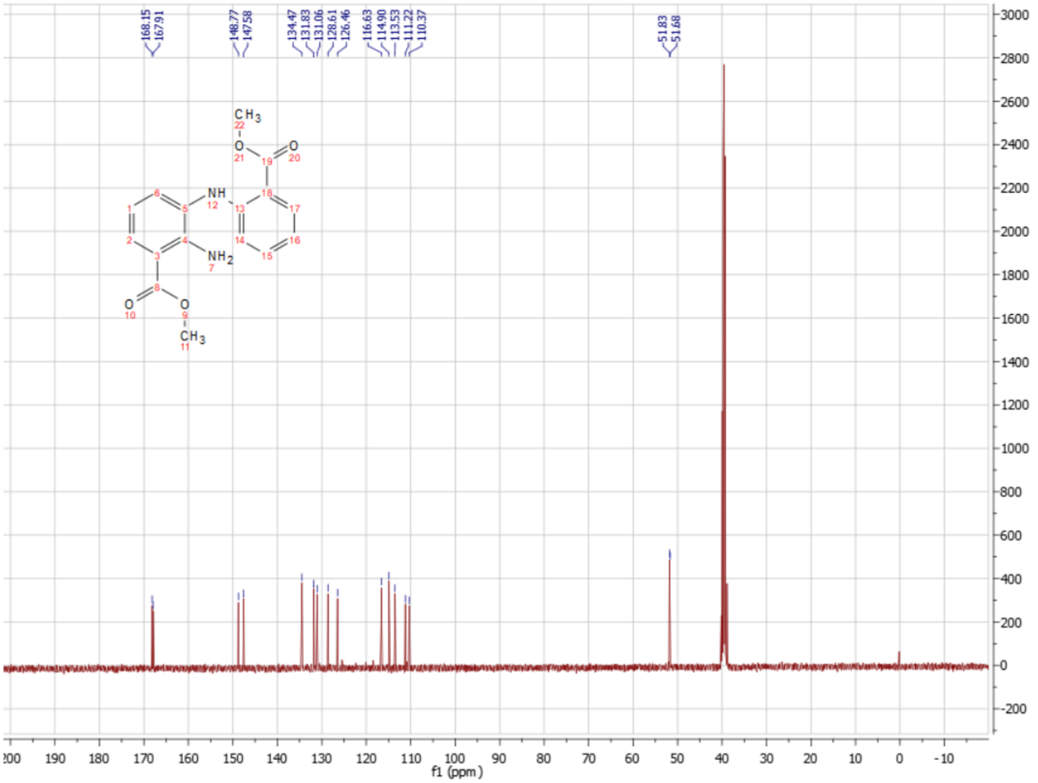

^{1}H NMR (400 MHz, DMSO) spectrum of compound **S21**

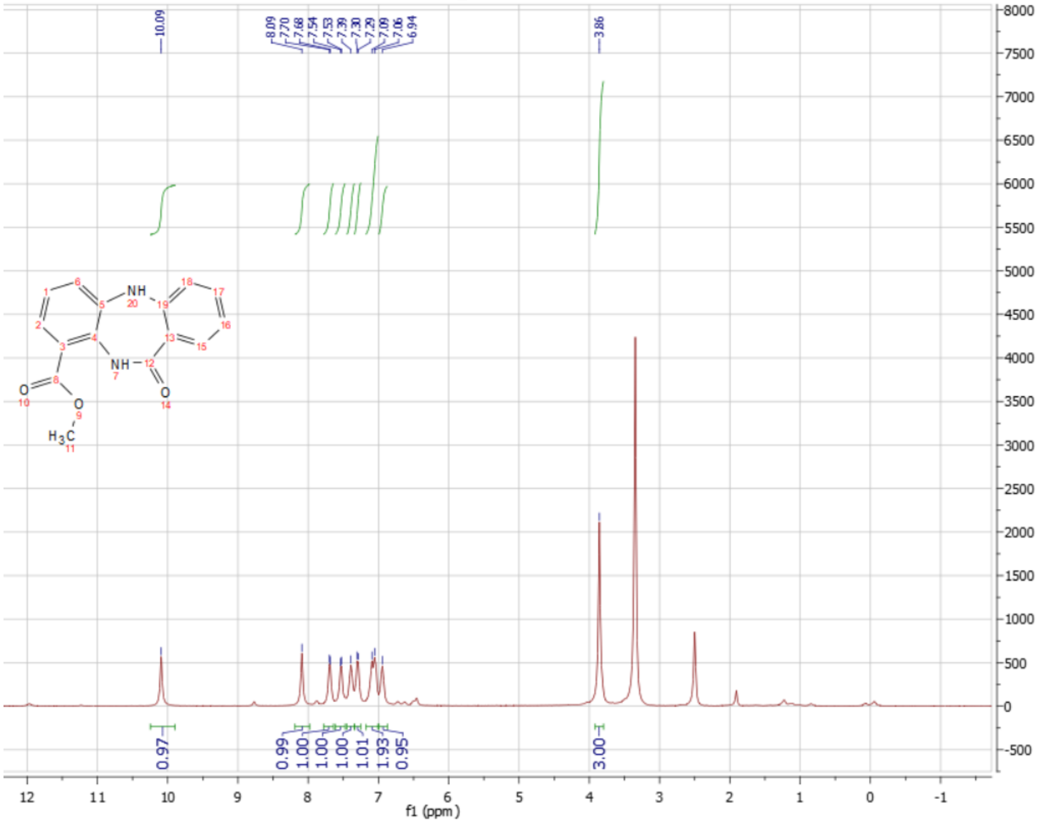

^{13}C NMR (101 MHz, DMSO) spectrum of compound **S21**

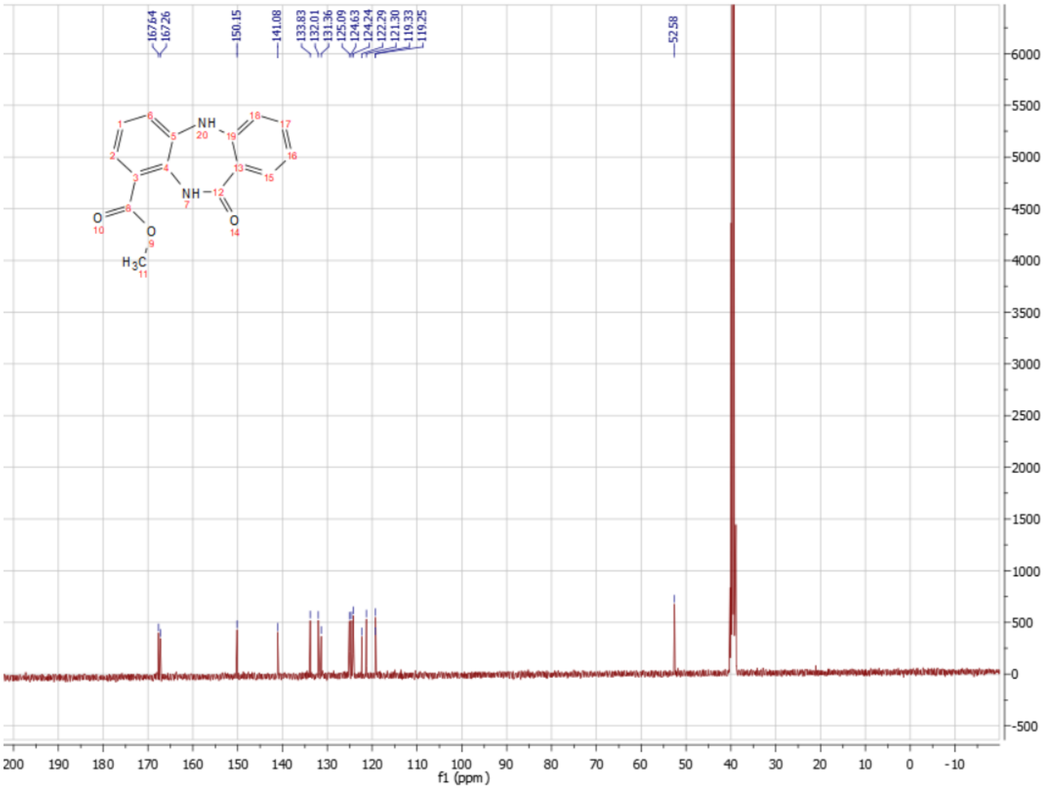

^{1}H NMR (400 MHz, DMSO) spectrum of compound **S22**

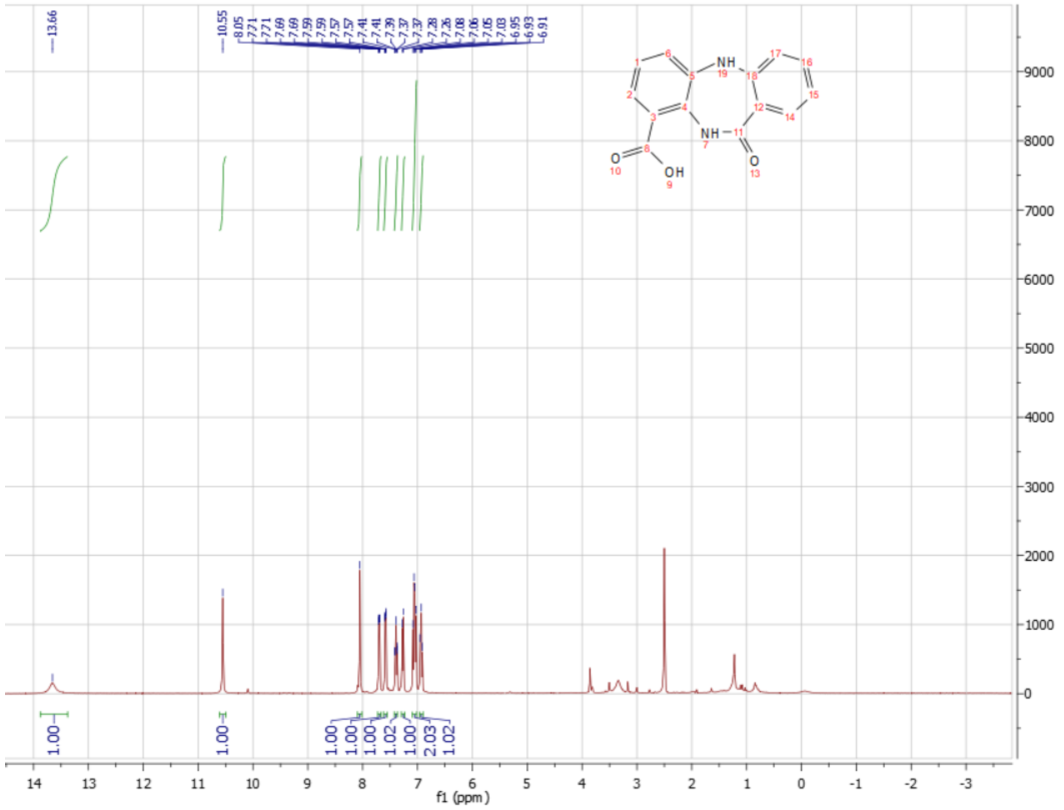

^{13}C NMR (101 MHz, DMSO) spectrum of compound **S22**

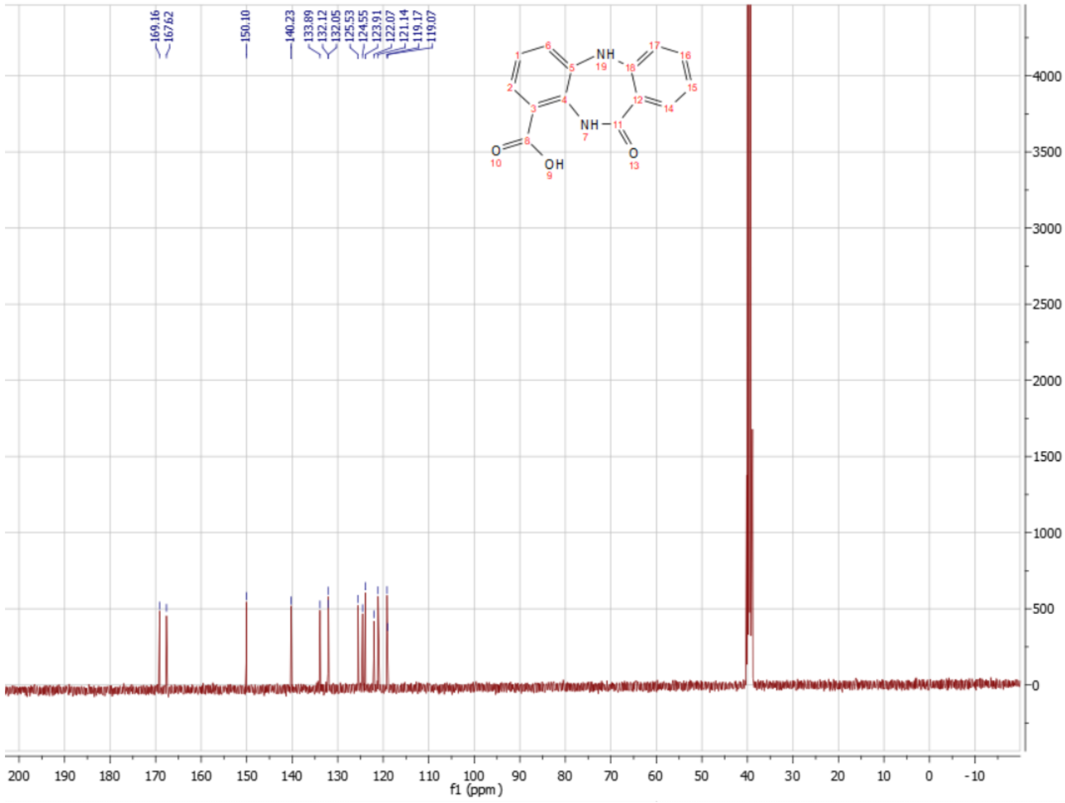

^{1}H NMR (400 MHz, DMSO) spectrum of compound **S24**

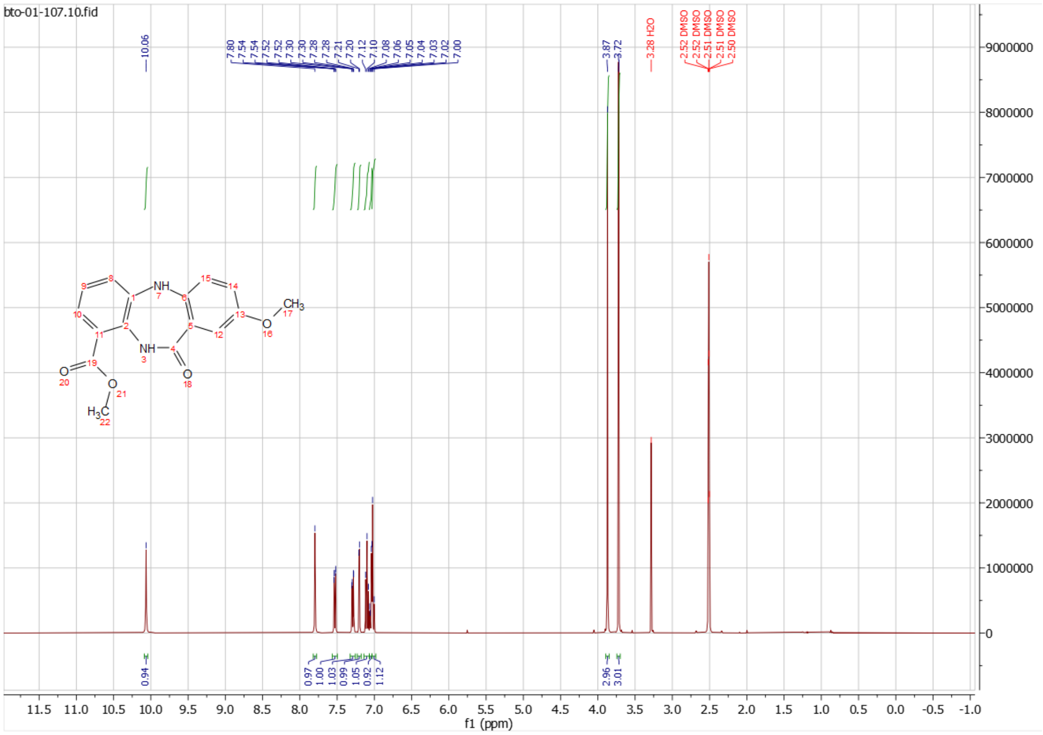

^{13}C NMR (126 MHz, DMSO) spectrum of compound **S24**

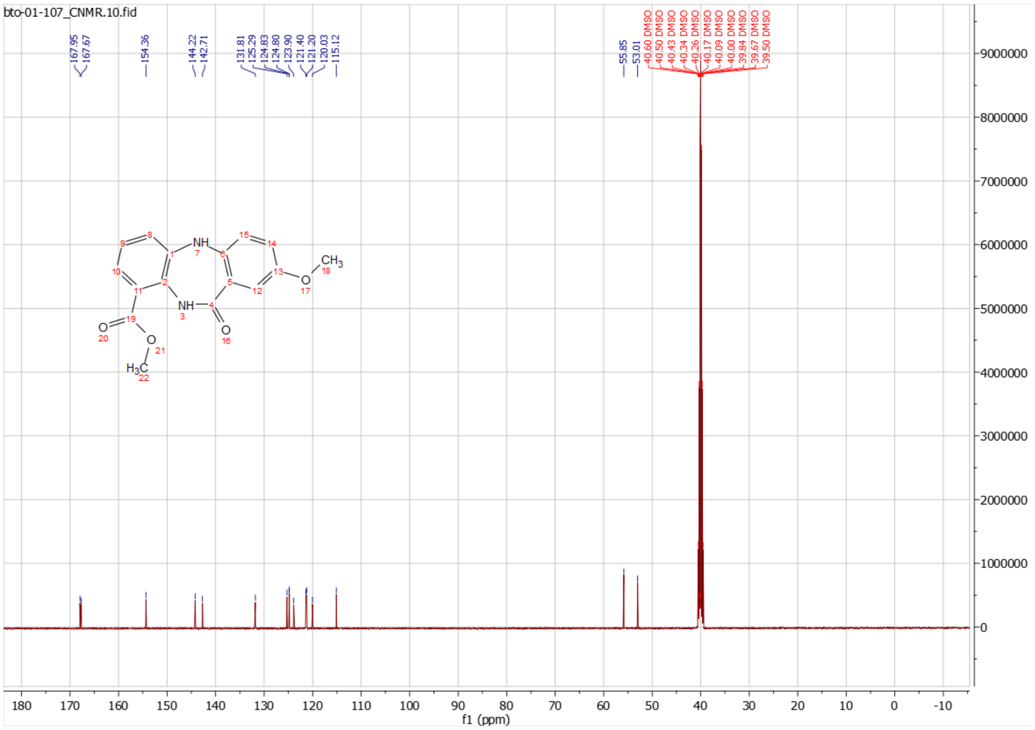

^{1}H NMR (400 MHz, DMSO) spectrum of compound **S25**

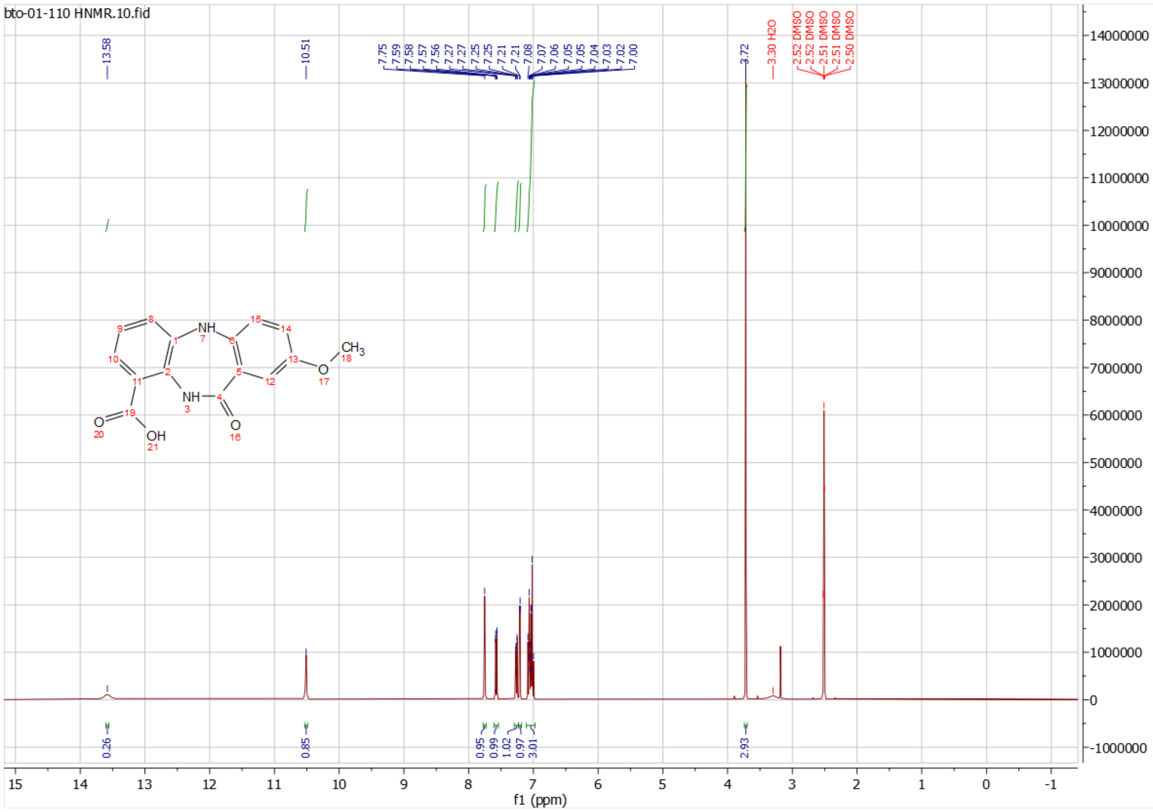

^{13}C NMR (126 MHz, DMSO) spectrum of compound **S25**

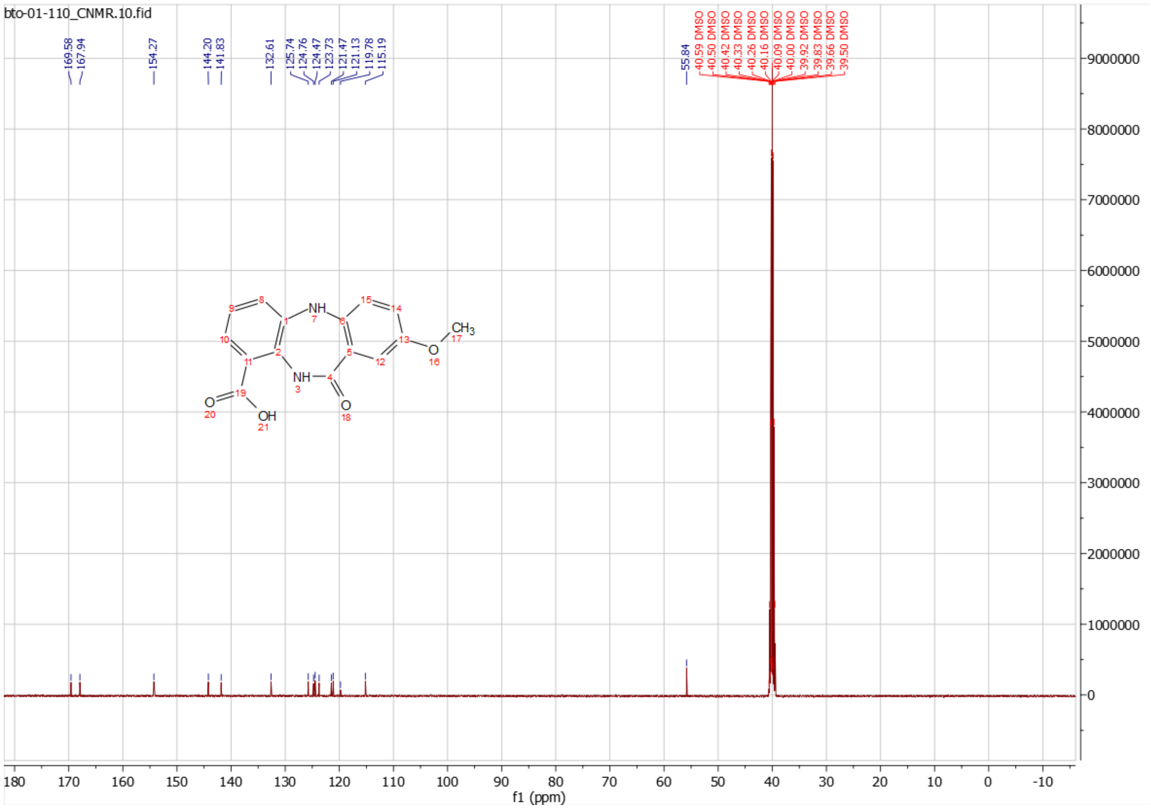

^{1}H NMR (400 MHz, DMSO) spectrum of compound **1**

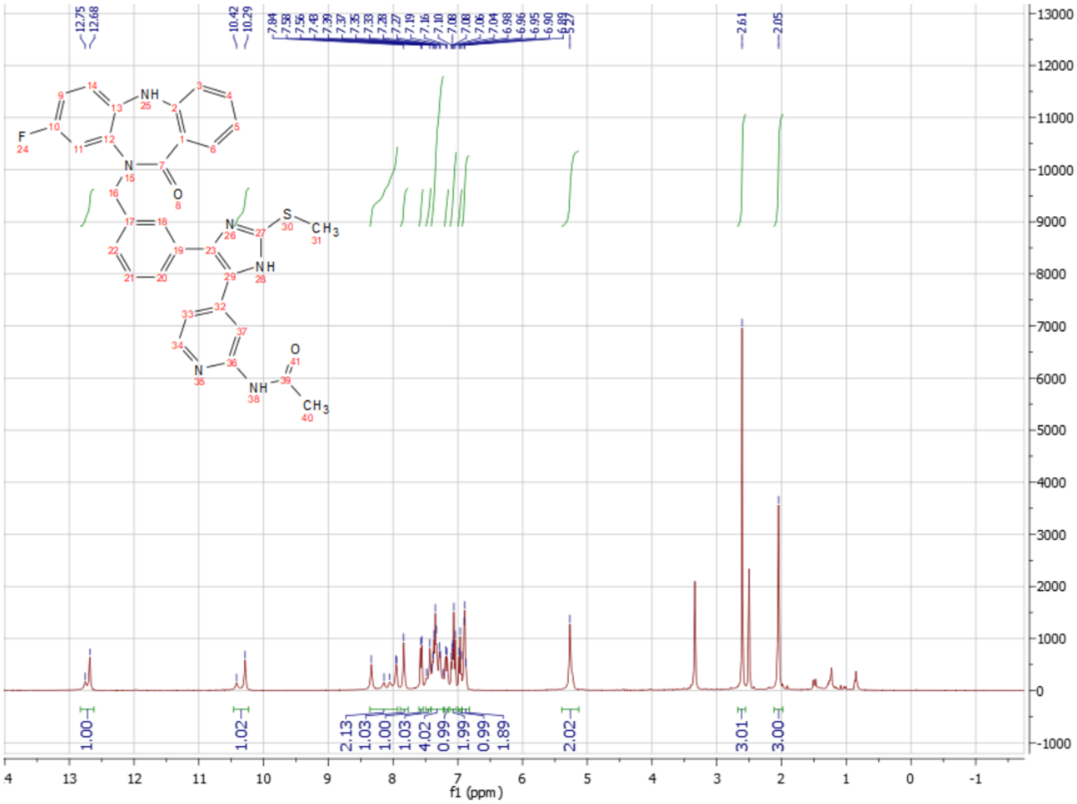

^{1}H NMR (400 MHz, DMSO) spectrum of compound **2**

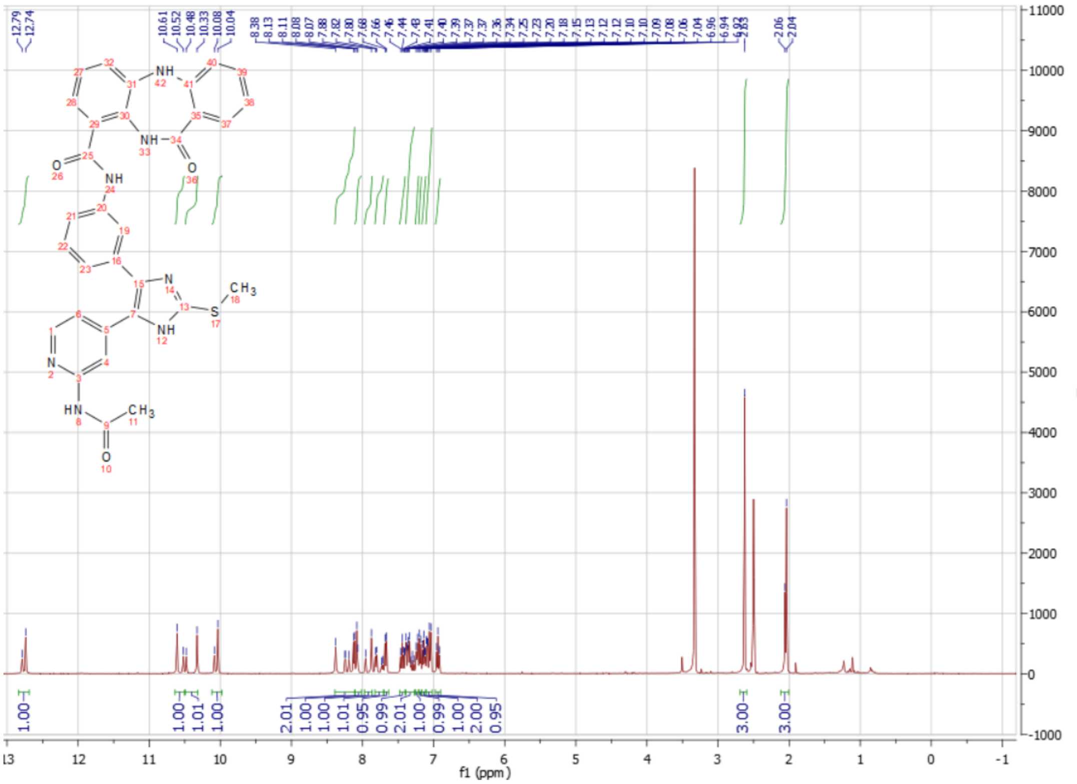

^{1}H NMR (400 MHz, DMSO) spectrum of compound **3**

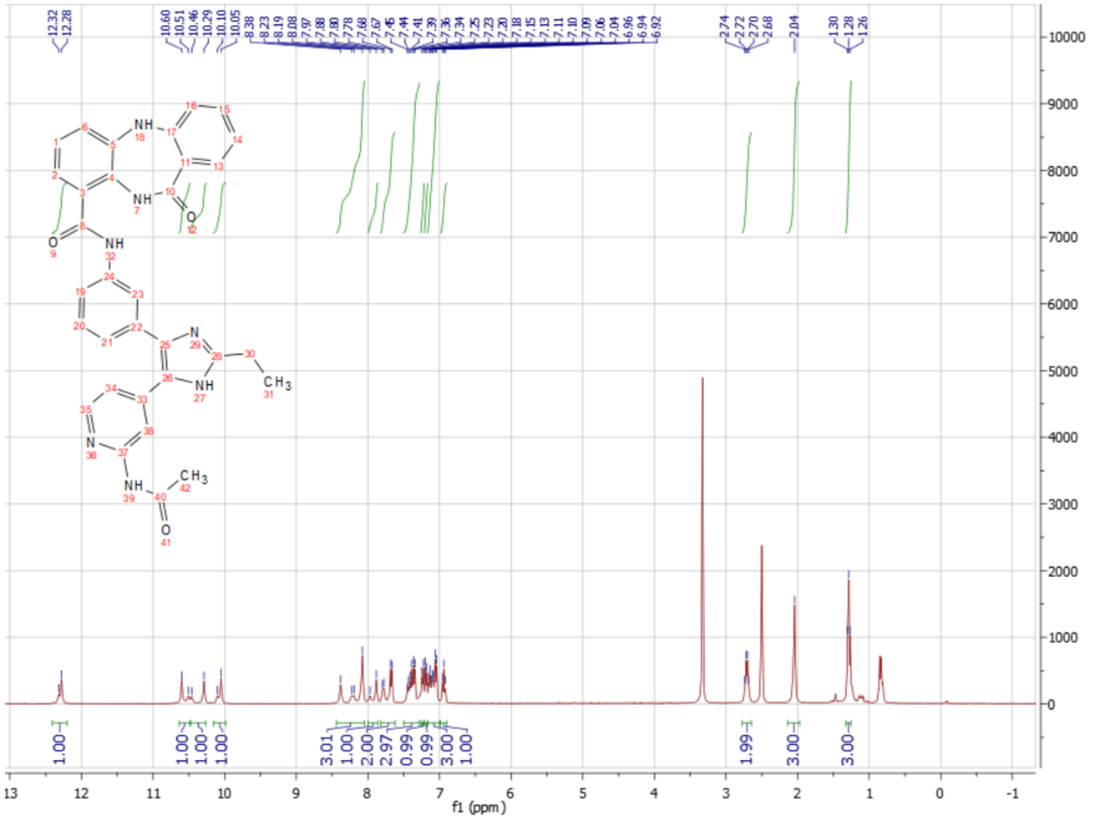

^{1}H NMR (400 MHz, DMSO) spectrum of compound **4**

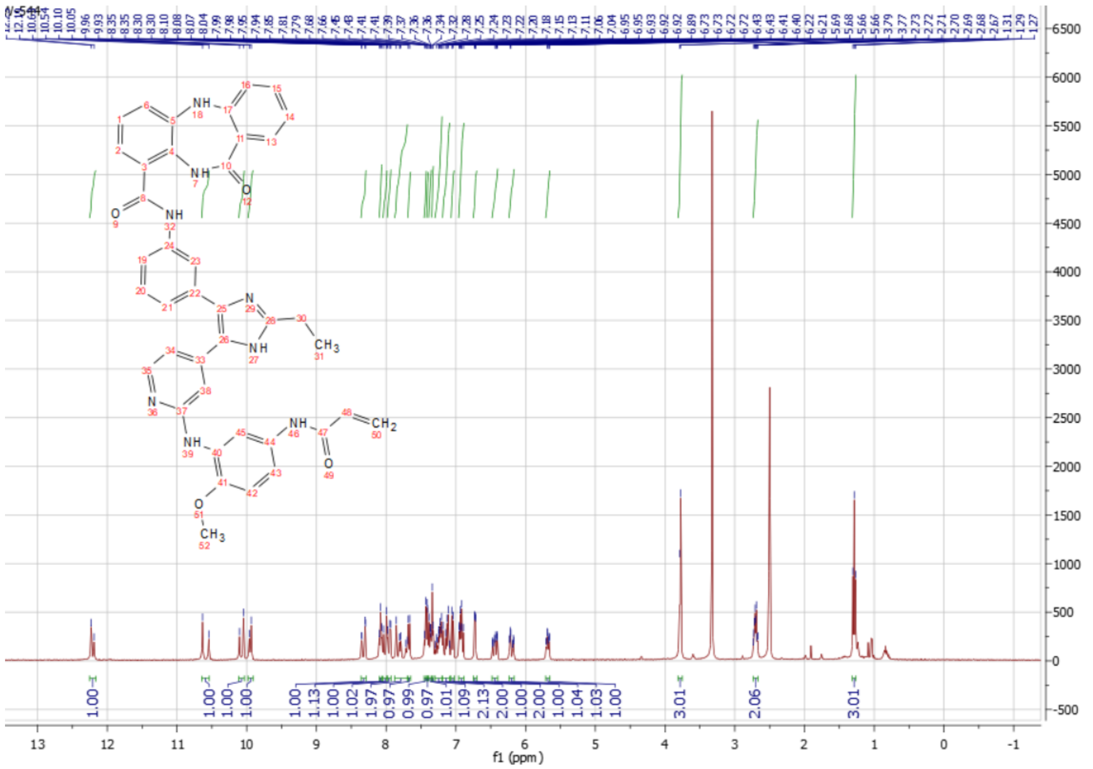

^{1}H NMR (400 MHz, DMSO) spectrum of compound **6**

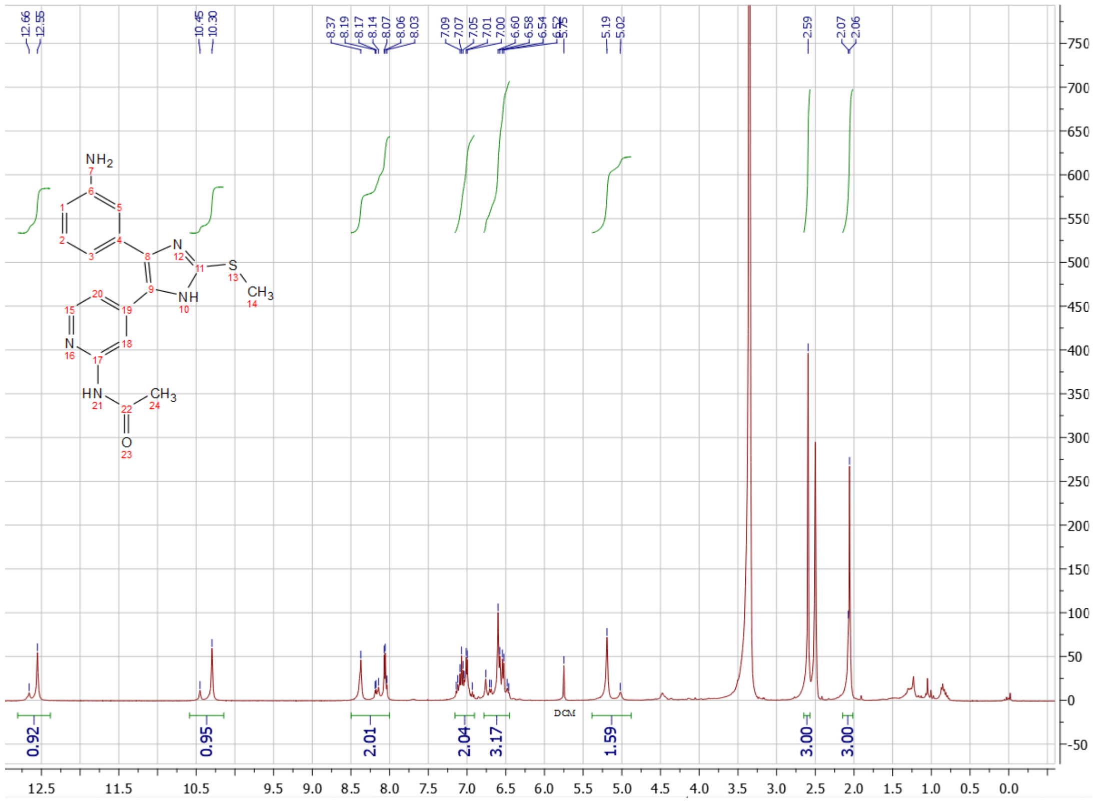

^{1}H NMR (400 MHz, DMSO) spectrum of compound **10**

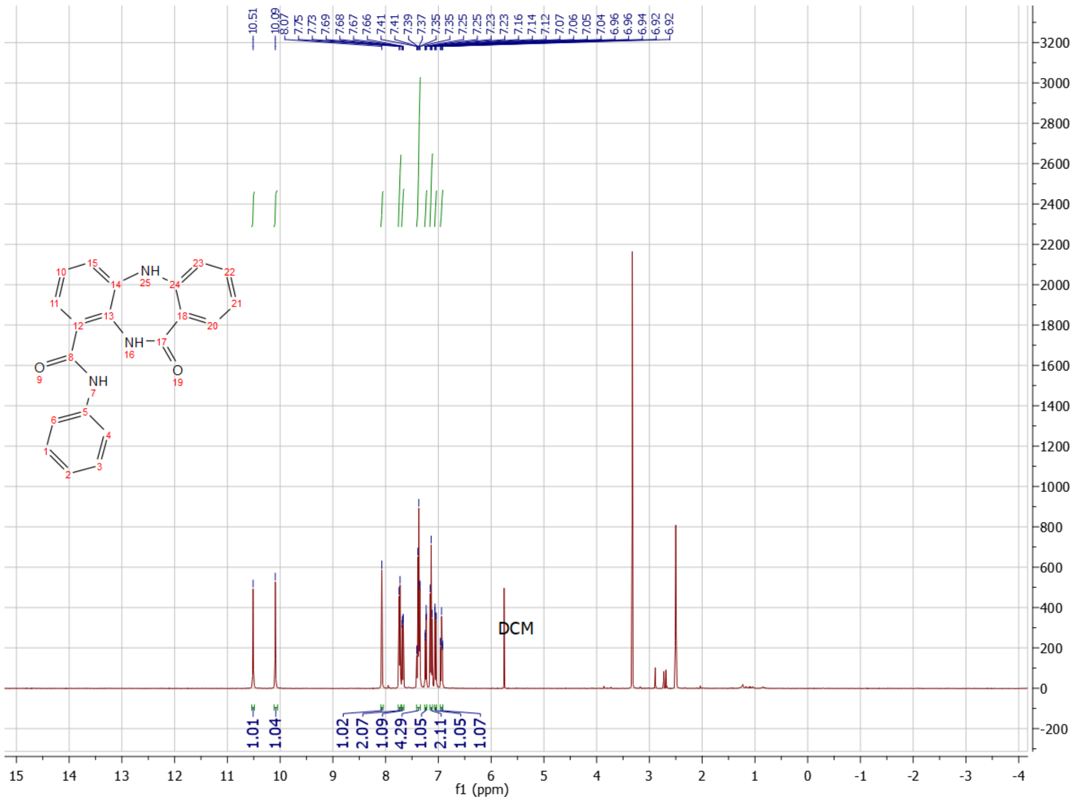

^{1}H NMR (400 MHz, DMSO) spectrum of compound **11**

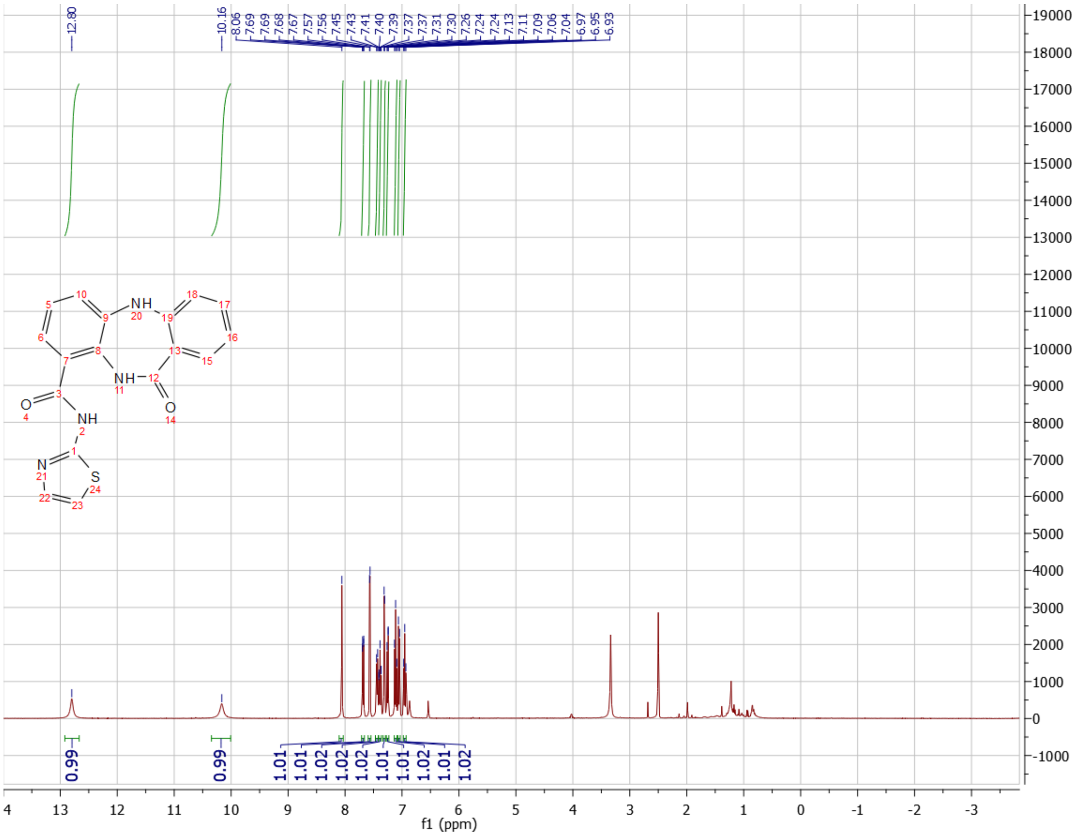

^{1}H NMR (500 MHz, DMSO) spectrum of compound **12**

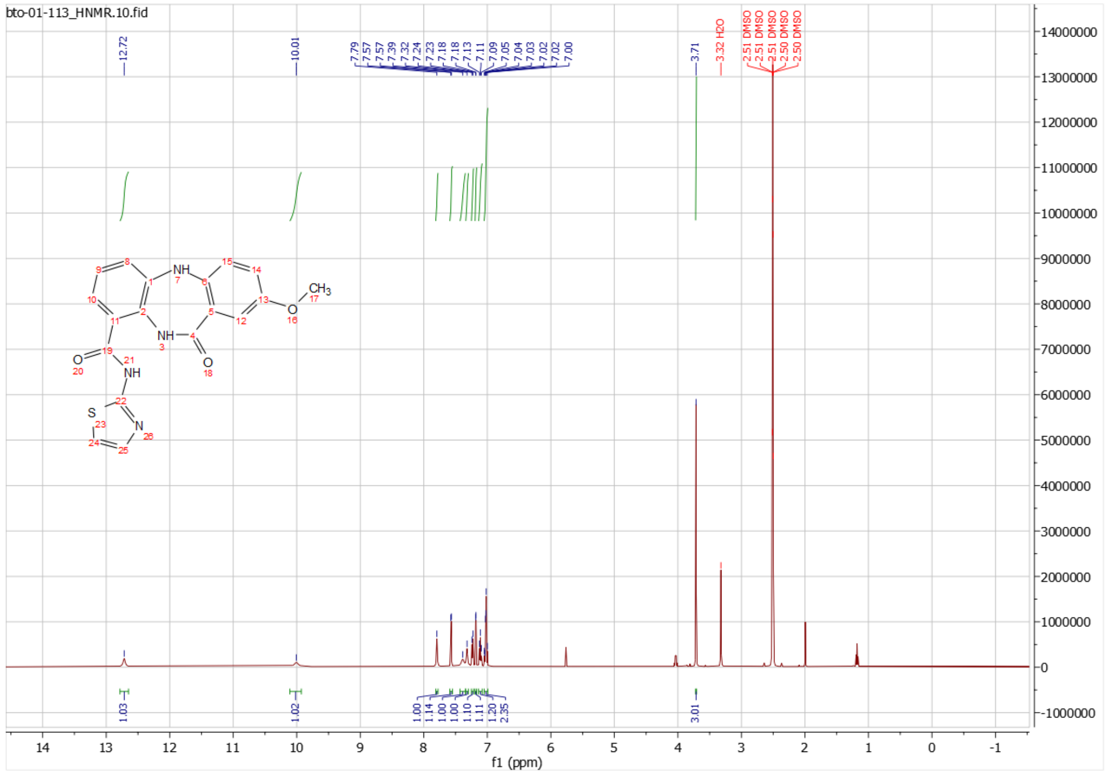

Supplement: Supplementary file 4 — Supplementary Data 1 [file 42004_2024_1108_MOESM4_ESM.pdf]
