# Supplementary material for: Linking ATP and allosteric sites to achieve superadditive binding with bivalent EGFR kinase inhibitors
Source: Commun Chem. 2024 Feb 20;7:38. doi: 10.1038/s42004-024-01108-3 (PMC10879502; doi:10.1038/s42004-024-01108-3)
Supplement: Supplementary file 8 — Reporting Summary [file 42004_2024_1108_MOESM8_ESM.pdf]

nature portfolio

Corresponding author(s): DBPR COMMSCHEM-23-0465-T

Last updated by author(s): Dec 5, 2023

Reporting Summary

Nature Portfolio wishes to improve the reproducibility of the work that we publish. This form provides structure for consistency and transparency in reporting. For further information on Nature Portfolio policies, see our Editorial Policies and the Editorial Policy Checklist.

Statistics

For all statistical analyses, confirm that the following items are present in the figure legend, table legend, main text, or Methods section.

n/a	Confirmed	
☐	☒	The exact sample size (n) for each experimental group/condition, given as a discrete number and unit of measurement
☐	☒	A statement on whether measurements were taken from distinct samples or whether the same sample was measured repeatedly
☒	☐	The statistical test(s) used AND whether they are one- or two-sided<br>*Only common tests should be described solely by name; describe more complex techniques in the Methods section.*
☐	☒	A description of all covariates tested
☒	☐	A description of any assumptions or corrections, such as tests of normality and adjustment for multiple comparisons
☐	☒	A full description of the statistical parameters including central tendency (e.g. means) or other basic estimates (e.g. regression coefficient) AND variation (e.g. standard deviation) or associated estimates of uncertainty (e.g. confidence intervals)
☒	☐	For null hypothesis testing, the test statistic (e.g. F, t, r) with confidence intervals, effect sizes, degrees of freedom and P value noted<br>*Give P values as exact values whenever suitable.*
☒	☐	For Bayesian analysis, information on the choice of priors and Markov chain Monte Carlo settings
☒	☐	For hierarchical and complex designs, identification of the appropriate level for tests and full reporting of outcomes
☒	☐	Estimates of effect sizes (e.g. Cohen's d, Pearson's r), indicating how they were calculated

Our web collection on statistics for biologists contains articles on many of the points above.

Software and code

Policy information about availability of computer code

Data collection	X-ray diffraction data was obtained on a Dectris EIGER2 X 16M detector. HTRF data was collected with a BMG Labtech Pherastar microplate reader. Time-dependent fluorescence was collected with the PerkinElmer ProxiPlate-384 Plus microplate reader. Western blots were visualized with the ChemiDoc MP imager (Bio-Rad) utilizing the Image Lab Touch Software (version 2.4.0.03). All NMR spectra were obtained with Bruker Avance 200 MHz and Bruker Avance 400 MHz spectrometers or with a Bruker Avance 600 MHz spectrometer (NMR Department, Institute of Organic Chemistry, Eberhard-Karls-Universität Tübingen) or Bruker Ascend 400 MHz and Bruker Ascend 500 MHz (Magnetic Resonance Center, Department of Chemistry, SUNY at Buffalo). Mass spectra were obtained by Advion TLC-MS (ESI) and from the MASS Spectrometry Department (ESI-HRMS), Institute of Organic Chemistry, Eberhard-Karls-Universität Tübingen or by Thermo Scientific LTQ XL Linear Ion Trap Mass Spectrometer (Small Instrument Center, Department of Chemistry, SUNY at Buffalo). HPLC analysis was performed on an Agilent 1100 Series Liquid chromatograph.
Data analysis	Graphpad Prism 9. Dials (v.1.11.2), xia2 (v.0.5.653), Phenix (v.1.20.1-4487) WinCoot (v.0.9.6.EL), eLBOW in Phenix (v.1.20.1-4487), PyMOL Molecular Graphics System (2.5.2), GLIDE (2021-2), Maestro (12.8.117)

For manuscripts utilizing custom algorithms or software that are central to the research but not yet described in published literature, software must be made available to editors and reviewers. We strongly encourage code deposition in a community repository (e.g. GitHub). See the Nature Portfolio guidelines for submitting code & software for further information.

Data

Policy information about availability of data

All manuscripts must include a data availability statement. This statement should provide the following information, where applicable:

- Accession codes, unique identifiers, or web links for publicly available datasets
- A description of any restrictions on data availability
- For clinical datasets or third party data, please ensure that the statement adheres to our policy

All source data were provided with this paper. All crystallography data are available in the PDB via the accession code 8FV3 and 8FV4

Human research participants

Policy information about studies involving human research participants and Sex and Gender in Research.

Reporting on sex and gender	N/A
Population characteristics	*Describe the covariate-relevant population characteristics of the human research participants (e.g. age, genotypic information, past and current diagnosis and treatment categories). If you filled out the behavioural & social sciences study design questions and have nothing to add here, write "See above."*
Recruitment	*Describe how participants were recruited. Outline any potential self-selection bias or other biases that may be present and how these are likely to impact results.*
Ethics oversight	*Identify the organization(s) that approved the study protocol.*

Note that full information on the approval of the study protocol must also be provided in the manuscript.

Field-specific reporting

Please select the one below that is the best fit for your research. If you are not sure, read the appropriate sections before making your selection.

☒ Life sciences ☐ Behavioural & social sciences ☐ Ecological, evolutionary & environmental sciences

For a reference copy of the document with all sections, see nature.com/documents/nr-reporting-summary-flat.pdf

Life sciences study design

All studies must disclose on these points even when the disclosure is negative.

Sample size	Three independent experiments were performed for all cellular assays.
Data exclusions	No data was excluded from any experiment
Replication	All attempts at replication were successful. Biochemical assays were performed n=2. This is consistent with prior published works. (Wittlinger et al. J. Med. Chem. 2022, Jia et al. Nature 2016, Beyett et al. Nature comm. 2022)
Randomization	Experiments were carried out with the number of replicates indicated in the manuscript. All replications were successful.
Blinding	No binding experiments were performed.

Reporting for specific materials, systems and methods

We require information from authors about some types of materials, experimental systems and methods used in many studies. Here, indicate whether each material, system or method listed is relevant to your study. If you are not sure if a list item applies to your research, read the appropriate section before selecting a response.

Materials & experimental systems

n/a	Involved in the study	
☐	☒	Antibodies
☐	☒	Eukaryotic cell lines
☒	☐	Palaeontology and archaeology
☒	☐	Animals and other organisms
☒	☐	Clinical data
☒	☐	Dual use research of concern

Methods

n/a	Involved in the study	
☒	☐	ChIP-seq
☒	☐	Flow cytometry
☒	☐	MRI-based neuroimaging

Antibodies

Antibodies used

Primary antibodies used; phospho-EGFR (Tyr1068; #2234, 1:1,000), EGFR (#4267; 1:1,000), phospho-AKT (Ser473; #4060, 1:1,000), AKT (#9272, 1:1,000), phospho-ERK1/2 (Thr202/Tyr204; #4370, 1:1,000), and ERK1/2 (#4695, 1:1,000) antibodies; were purchased from Cell Signaling Technology. Secondary Goat anti-rabbit IgG starbright blue 700 (Biorad, 64484700) and Anti-tubulin hFAB Rhodamine Tubulin (Bio-Rad, 64512248).

Validation

Each primary antibody has been validated to cross react with the species from which the cell lines and tumors were derived, as done in earlier studies of mutant EGFR drug characterization. Detailed validation data, relevant citation and pertinent information regarding the antibody are provided on the manufacturer's websites.

Eukaryotic cell lines

Policy information about cell lines and Sex and Gender in Research

Cell line source(s)

H1975, H3255, H3255GR and HCC827 lung adenocarcinoma cells are from human origin and were obtained from Dr. Pasi Jänne in Dana-Farber Cancer Institute (2022) and previously purchased from ATCC. Parental Ba/F3 cells from mouse was a generous gift from the laboratory of Dr. David Weinstock (in 2014), Dr. Pasi Jänne (2020) both of the Dana-Farber Cancer Institute and was used to generate the wildtype EGFR, L858R, and L858R/T790M EGFR mutant Ba/F3 cells

Authentication

All cell lines are not authenticated at this time

Mycoplasma contamination

All human cell lines were tested negative for Mycoplasma using Myco-Sniff-Rapid™ Mycoplasma Luciferase Detection Kit Plus PCR Primer Set (MP biomedicals, 0930504-CF). The Ba/F3 All cell lines were tested negative for Mycoplasma using Mycoplasma Plus PCR Primer Set (Agilent). All cell lines were passaged and/or used for no longer than 4 weeks for all experiments.

Commonly misidentified lines
(See ICLAC register)

No commonly misidentified lines were used in our studies.
